# Lipidomic profiling of non-mineralized dental plaque and biofilm by untargeted UHPLC-QTOF-MS/MS and SWATH acquisition

**DOI:** 10.1007/s00216-019-02364-2

**Published:** 2020-01-15

**Authors:** Bernhard Drotleff, Simon R. Roth, Kerstin Henkel, Carlos Calderón, Jörg Schlotterbeck, Merja A. Neukamm, Michael Lämmerhofer

**Affiliations:** 1 Institute of Pharmaceutical Sciences, Pharmaceutical (Bio-)Analysis, University of Tübingen, Auf der Morgenstelle 8, 72076 Tübingen, Germany; 2 Institute of Forensic Medicine, Medical Center-University of Freiburg, Faculty of Medicine, University of Freiburg, Albertstraße 9, 79104 Freiburg, Germany

**Keywords:** Dental plaque, Biofilm, Untargeted lipidomics, Data-independent acquisition, SWATH

## Abstract

Dental plaque is a structurally organized biofilm which consists of diverse microbial colonies and extracellular matrix. Its composition may change when pathogenic microorganisms become dominating. Therefore, dental biofilm or plaque has been frequently investigated in the context of oral health and disease. Furthermore, its potential as an alternative matrix for analytical purposes has also been recognized in other disciplines like archeology, food sciences, and forensics. Thus, a careful in-depth characterization of dental plaque is worthwhile. Most of the conducted studies focused on the screening of microbial populations in dental plaque. Their lipid membranes, on the other hand, may significantly impact substance (metabolite) exchange within microbial colonies as well as xenobiotics uptake and incorporation into teeth. Under this umbrella, a comprehensive lipidomic profiling for determination of lipid compositions of in vivo dental plaque samples and of in vitro cultivated biofilm as surrogate matrix to be used for analytical purposes has been performed in this work. An untargeted lipidomics workflow utilizing a ultra-high-performance liquid chromatography (UHPLC)-quadrupole-time-of-flight (QTOF) platform together with comprehensive SWATH (sequential window acquisition of all theoretical fragment ion mass spectra) acquisition and compatible software (MS-DIAL) that comprises a vast lipid library has been adopted to establish an extensive lipidomic fingerprint of dental plaque. The main lipid components in dental plaque were identified as triacylglycerols, followed by cholesterol, cholesteryl esters as well as diacylglycerols, and various phospholipid classes. In vivo plaque is a rare matrix which is usually available in very low amounts. When higher quantities for specific research assays are required, efficient ways to produce an appropriate surrogate matrix are mandatory. A potential surrogate matrix substituting dental plaque was prepared by cultivation of in vitro biofilm from saliva and similarities and differences in the lipidomics profile to in vivo plaque were mapped by statistical evaluation post-analysis. It was discovered that most lipid classes were highly elevated in the in vitro biofilm samples, in particular diacylglycerols, phosphatidylglycerols, and phosphatidylethanolamines (PEs). Furthermore, an overall shift from even-chain lipid species to odd-chain lipids was observed in the cultivated biofilms. On the other hand, even-chain phosphatidylcholines (PCs), lysoPCs, cholesteryl esters, and cholesterol-sulfate were shown to be specifically increased in plaque samples.

**Electronic supplementary material:**

The online version of this article (10.1007/s00216-019-02364-2) contains supplementary material, which is available to authorized users.

**Figure:**
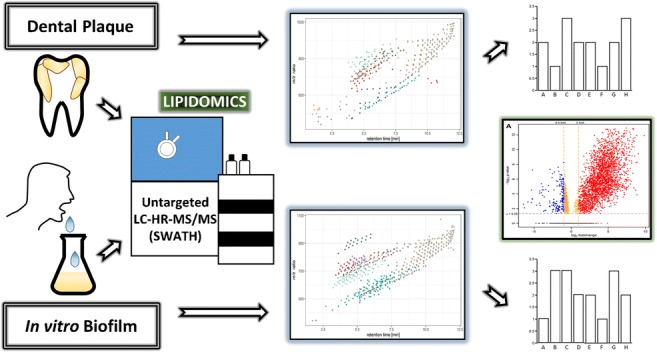
Graphical abstract

## Introduction

Non-mineralized dental biofilm (plaque) consists of a community of diverse microorganisms, which is embedded in a matrix of extracellular polymeric substances (EPS). The EPS matrix comprises macromolecules of microbial origin such as (lipo)polysaccharides, (glycol)proteins, and lipids [1]. The lipid profile of (dental) biofilms is of particular interest as it allows drawing some conclusions on the bacterial composition and its possible alteration from symbiosis under healthy conditions to dysbiosis (biofilm dominated by pathogenic bacteria) [2]. Lipids constitute the bacterial cell membrane and may be characteristic for different bacterial species. For example, they may differ in their fatty acid profiles constituting the membrane lipids. In general, besides saturated and unsaturated fatty acids, bacteria can contain fatty acids with additional hydroxyl, methyl substituents, or even cyclic ring structure.

Currently, there are two major approaches to characterize microbial communities with regard to their lipid profiles: phospholipid fatty acid analysis (PLFA) [3–6] and direct analysis of all esterified fatty acids [7, 8]. PLFA requires fractionation into neutral and glyco- and phospholipid classes beforehand, and has the advantage of providing good estimates on viable biomass because it relies on the rapid turnover of phospholipids after cell death. In the case of direct analysis without prior fractionation, all esterified lipids are analyzed, which can slightly bias the results of viability estimates and is less suitable to distinguish bacterial and fungal community structures [9]. Both methods are mainly used to characterize microbial communities in ecosystem studies with some exceptions [10]. Recently, a direct fatty acid analysis method was successfully adopted to characterize microbial communities in dental plaque [11]. All the methods above rely on the analysis of previously identified fatty acids associated with certain microbial species. This targeted approach can be useful, when applied to strictly defined microbial cultures [12] or when only viability estimates or changes in community structure, following certain environmental changes, are to be monitored [4]. GC-MS profiling via fatty acid methyl esters (FAME), however, leads to loss of the information to which head group (lipid class) the fatty acids were associated.

For a more comprehensive and detailed characterization of lipid profiles in dental plaque, untargeted lipidomics techniques, which typically utilize high-resolution mass spectrometry (MS), e.g., by quadrupole time-of-flight (QTOF) instruments or Q-orbitrap, are convenient and powerful analytical tools [13]. These methods capture lipid analytes across a great variety of lipid classes and allow retrospective (re-)analysis of the data. Post-acquisition identification of the lipid signals is based on availability of MS/MS spectra. This can be achieved by data-dependent acquisition (DDA, also termed information-dependent acquisition (IDA)) [14] in which MS/MS spectra are triggered in dependence on an MS scan for the most abundant signals. More informative, better reproducible, and more robust are modern data-independent acquisition (DIA) strategies like SWATH (sequential window acquisition of all theoretical fragment ion mass spectra) [15, 16] which aims to maximize the extractable sample information and yield an enhanced number of identified features compared with DDA [17]. As these assays produce large amounts of data, sophisticated bioinformatic tools for data mining have become indispensable for the full exploitation of the potential of untargeted lipidomics. Additional bioinformatic efforts are required for spectral deconvolution, as in DIA spectral quality may be compromised by contaminating ions due to co-isolation of various co-eluting precursors in the same Q1 precursor isolation window.

In this work, we employed an untargeted lipidomic profiling workflow via ultra-high-performance liquid chromatography (UHPLC)-QTOF-MS/MS and SWATH acquisition for the determination of the lipid profile of non-mineralized dental plaque that was grown in vivo. As dental plaque is a scarce matrix that can only be collected in small quantities under conditions of regular dental care [18], several procedures to obtain artificial plaque for further scientific experimentation have been proposed [19–23]. Along this line, we compared the lipid composition of in vitro biofilm, which had been cultivated from saliva samples [18], to in vivo dental plaque and, moreover, investigated the effects of short-term plaque aging in a time range of 72 h.

## Materials and methods

### Materials

Acetonitrile (MeCN, Ultra LC-MS grade), 2-propanol (IPrOH, Ultra LC-MS grade), and formic acid (98%, *w*/*v*, ACS grade) were purchased from Carl Roth (Karlsruhe, Germany). Type I purity water was obtained from a Purelab Ultra purification system (ELGA LabWater, Celle, Germany). Ammonium formate was supplied by Sigma-Aldrich (St. Louis, MO, USA). SPLASH LipidoMIX (Lipidomix) was supplied by Avanti Polar Lipids (Alabaster, AL, USA). Oxoid Tryptone Soya Broth (TSB medium) was supplied by Thermo Fisher Scientific (Dreieich, Germany) and sterile isotonic sodium chloride (NaCl) solution was purchased from Braun (Melsungen, Germany).

### Lipid nomenclature and abbreviations

In order to simplify the discussion of the results, common abbreviations and nomenclature rules [24–26] are used to describe the detected lipid species. The following lipid classes were identified: CE, cholesteryl ester; Cer-AP, ceramide alpha-hydroxy fatty acid-phytospingosine; Cer-BDS, ceramide beta-hydroxy fatty acid-dihydrosphingosine; Cer-BS, ceramide beta-hydroxy fatty acid-sphingosine; Cer-NDS, ceramide non-hydroxy fatty acid-dihydrosphingosine; Cer-NS, ceramide non-hydroxy fatty acid-sphingosine; DAG, diacylglycerol; DGDG, digalactosyldiacylglycerol; FA, free fatty acid; HBMP, hemibismonoacylglycerophosphate; HexCer, hexosylceramide; HexCer-NDS, hexosylceramide non-hydroxy fatty acid-dihydrosphingosine; HexHexCer, dihexosylceramide; LPC, lysophosphatidylcholine; LPE, lysophosphatidylethanolamine; MGDG, monogalactosyldiacylglycerol; PC, phosphatidylcholine; PE, phosphatidylethanolamine; PE-Cer, ceramide phosphoethanolamine; PI, phosphatidylinositol; SHexCer, sulfatide; SM, sphingomyelin; TAG, triacylglycerol. For further specification, a lowercase “e” was used to describe ether-linked lipid species, a “+O” designates an oxidized species, and a lowercase “d” or “t” indicate a dihydroxy or trihydroxy species, respectively. When information about the sn positions of the fatty acyl side chains was available, a “/” was used if the exact position could be proven and a “–” was used if the configuration could not be completely resolved [24–26]. Furthermore, stereospecific numbering (sn) was added to appoint the position of acylation or alkylation of the glycerol group, if known.

### Collection of in vivo dental plaque samples

The formation of plaque on the enamel is initiated immediately after cleaning the teeth [27]. The ensuing development of dental plaque is accompanied by a continuing mineralization or calcification process that is completed after approximately 12 days [28]. Due to the constant changes of dental plaque during aging, this study focused on the investigation of early stages in plaque formation. In vivo plaque samples were collected from one healthy donor on eight different sampling occasions. The plaque was scraped off the teeth of the upper and lower jaw using a micro-scaler after either 24 (*n* = 4) or 72 h (*n* = 4) without tooth brushing. Each individual sample was stripped off on the rim of a 1.5-mL Eppendorf cup and was dried at room temperature for approximately 2 h.

### Cultivation of in vitro biofilm samples

In vitro biofilm was cultivated according to a previously described protocol by Henkel et al. [18]. Saliva samples from ten volunteers were collected. One milliliter of each sample was pipetted in separate tubes containing 20 mL of TSB medium (BF 1–10). Two additional samples (BF 11 and 12) were created by pooling the remaining saliva of volunteers 1 to 5 and 6 to 10, respectively, and these pools were treated accordingly. The test tubes were capped with pierced caps to allow air exchange and were placed into a 37 °C water bath. Daily, 10 mL of the medium was replaced by 10 mL of fresh TSB medium. On the third day, the biofilm cultures were centrifuged, the supernatant was removed, and the biofilm pellets were washed three times with 2 mL of sterile isotonic NaCl solution. The washed pellets were then dried at 40 °C under a gentle stream of nitrogen and were milled to a fine powder in a Retsch MM400 ball mill (Retsch, Haan, Germany) for 30 s at 20 Hz. Subjects providing samples (dental plaque and saliva) gave informed consent to the study that was positively reviewed by the University Clinic of Freiburg ethics committee (DRKS ID no.: DRKS00011148).

### Sample preparation

The dried samples were stored at − 20 °C until analysis. To ensure the extraction of a broad spectrum of lipids, protein precipitation and extraction by IPrOH was chosen as sample preparation strategy [29, 30]. Prior to protein precipitation and extraction, respectively, the solvent was spiked with 1% of Lipidomix, a quantitative standard mixture of deuterated lipids of various lipid classes (see Electronic Supplementary Material (ESM) Table S1), to enable additional options for internal standard (IS)-based normalization post-acquisition. Moreover, these stable isotope-labeled lipids were employed for class-specific lipid quantification via one-point calibration. After addition of 100 μL of spiked IPrOH, the samples were thoroughly vortexed and subjected to ultrasonication (Sonorex RK 510s; Bandelin, Berlin, Germany) for 10 min at a frequency of 35 kHz and a power density of 32 W/L. Subsequently, the extracts were centrifuged at 4 °C and 15,000×*g* for 10 min with a 5415R microcentrifuge (Eppendorf, Hamburg, Germany) and each supernatant was transferred to a 250-μL conical glass insert in a 1.5-mL glass vial. Quality control (QC) samples for normalization via linear weighted scatter plot smoothing [31] (LOWESS) and monitoring of instrument performance were created by pooling equal amounts of processed sample supernatants. The QCs were embedded and distributed in dense intervals into the analytical sequence of study samples. Subsequently, sample vials were sealed with a crimp cap and stored at 4 °C in the autosampler for the time of analysis. The sample sequence (see ESM Table S2) was started within 2 h after preparation.

### LC method

Lipid species separation was performed according to the method reported by Tsugawa et al. [32]. The chromatographic system consisted of a 1290 Infinity UHPLC system (Agilent Technologies, Waldbronn, Germany) and was operated with an Acquity UPLC CSH C18 column (100 mm × 2.1 mm, 1.7 μm, 130 Å) and a VanGuard Acquity UPLC CSH C18 pre-column (5 mm × 2.1 mm, 1.7 μm, 130 Å) (Waters, Milford, MA, USA). Mobile phase A was a mixture of 60:40 MeCN:H_2_O (v/v) with 0.1% formic acid (v/v) and 10 mM ammonium formate and mobile phase B contained 90:9:1 IPrOH:MeCN:H_2_O (v/v/v) with 0.1% formic acid (v/v) and 10 mM ammonium formate. The following gradient was run with a total flow rate of 0.6 mL min^−1^: 0.0 min, 15% B; 2.0 min, 30% B; 2.5 min, 48% B; 11.00 min, 82% B; 11.50 min, 99% B; 12.00 min, 99% B; 12.10 min, 15% B, 15.00 min, 15% B. Sample injection was conducted by a PAL HTC-xt autosampler (CTC Analytics, Zwingen, Switzerland) and injection volume was set to 3 μL in positive and 5 μL in negative ionization modes. This way, the typically decreased sensitivity in negative mode was anticipated to be partially compensated.

### MS method

The analytical system was connected to a TripleTOF 5600+ mass spectrometer, which was operated with the electrospray ionization (ESI) probe of a DuoSpray ion source (Sciex, Framingham, MA, USA). The following source parameters were used for untargeted lipid detection: curtain gas (N_2_), 35 psi; nebulizer gas (N_2_), 60 psi; heater gas (N_2_), 60 psi; ion source voltage floating, +5500 V (positive mode) and − 4500 V (negative mode); declustering potential,: ±80 V; source temperature, 350 °C. The experimental MS setup consisted of a TOF-MS experiment for precursor detection in the mass range of *m/z* 50–1250 with an accumulation time of 200 ms. For comprehensive recording of MS/MS spectra, SWATH acquisition [15] was utilized with a collision energy of ± 45 V and a spread of ± 15 V. For each ionization mode, 20 SWATH-MS/MS experiments were created (see ESM Table S3). Ionization mode-dependent selection of SWATH window widths was achieved by swathTUNER [33]. The data input for the SWATH design was acquired from preliminary measurements of aliquots from pooled in vitro biofilm QC samples (BF11 and BF12) using IDA and the resultant TOF-MS data. SWATH settings were chosen to achieve an optimized distribution of the precursor density per SWATH window. Accumulation time for each SWATH-MS/MS experiment was set to 25 ms. Total cycle time summed up to 750 ms, which yielded a minimum of 10 points per peak with an average peak width at base of 8 s. Resolving power of the instrument was verified to reach the specified values of > 30,000 (FWHM @ *m*/*z* 829.5393) on TOF-MS level and > 30,000 (FWHM @ *m*/*z* 397.2122) on SWATH-MS/MS level using the high resolution mode. The sample sequence was firstly analyzed in negative and subsequently in positive ionization mode. Mass calibration was performed by automated infusion of sodium acetate (0.1 mg mL^−1^ in MeCN:H_2_O, 1:1, v/v) after every tenth sample via the formed clusters. The analytical system was controlled by the Analyst 1.7 TF software (Sciex).

### Data processing

The used LC-MS setup enables enhanced and reliable lipid identification via a well-established data processing workflow [34], which is utilizing the SWATH-compatible MS-DIAL software tool [32] (version 3.96). The software covers the data processing steps of peak finding, blank subtraction, feature alignment, normalization, MS/MS spectral deconvolution, and score-based lipid identification, which is relying on retention time (*t*_R_), accurate mass, and isotopic pattern similarity, as well as MS/MS similarity of the analyte data to the heuristically modeled in silico LipidBlast library [35] and other internal MS-DIAL databases (Lipidomics DB VS54, unpublished). A total similarity score of 80% together with a *t*_R_ deviation tolerance of 1.0 min was deemed acceptable as thresholds for initial lipid identification. Exemplary, deconvoluted MS/MS spectra of one representative of each identified lipid class, together with the corresponding reference spectra from the library, are shown in ESM Table S14. In order to enhance the yield of identified lipids, data processing via MS-DIAL was executed with a low intensity threshold (100 counts per second (cps)) for extensive feature collection in lipidomic profiling projects (see ESM Table S4). Three blank samples (IPrOH) were analyzed in each mode for blank subtraction. Here, features detected in blank samples were automatically excluded from alignment in each MS-DIAL project unless they showed a fold change > 5 in the average corresponding real samples. Missing data was filled by compulsion via feature detection within ± 5 data points even if no local maximum was observed. Persisting missing data points were imputed by replacing them with a value representing 10% of the minimum peak height over all samples (default setting of MS-DIAL). Identified lipid species were only considered if detected in at least 50% of samples of respective groups; otherwise, these features were considered as unknown for further analysis. Subsequently, to control for misannotations of the automated lipid identification procedure of MS-DIAL, regularly ordered intra-class elution patterns, which result in reversed-phase (RP)-chromatography due to homologous series of lipids and are dependent on carbon chain length as well as degree of saturation of lipid species, were visually checked for validation of structural annotations [36] (see ESM Table S15). Previously (by MS-DIAL) identified lipids that did not match the respective elution pattern were annotated as unknown features. For comparison of two experimental groups or for the evaluation of general lipidomic profiles, data was processed covering only the respective groups of interest. Thus, it was avoided to introduce bias in feature coverage that could have derived from other experimental groups and consequential gap filling or missing value imputation. The detailed processing parameters are listed in ESM Table S4.

Prior to further processing steps, aligned peak height raw data was exported from MS-DIAL and normalized to sample weight (see ESM Table S2) (IS data was not normalized). For each comparison of experimental groups and each ionization mode, IS-based normalization by different methods (CCMN [37], NOMIS [38], B-MIS [39], RUVrandom [40]) was performed via the signal intensities of the deuterated lipid standards of the added Lipidomix (see ESM Table S1). In addition, LOWESS normalization was conducted by using QC samples. The performances of the individual normalization methods were evaluated according to published guidelines on the selection of normalization strategies [41] (relying on the reduction of metrics of intra-group variance like coefficient of variation (CV), median absolute deviation (MAD), and variance (Var) in QCs and experimental groups) and the best-performing dataset was chosen for further statistical analysis. Moreover, CV distributions in QC samples for both polarity modes are shown in ESM Fig. S10.

Fold changes of features between experimental groups were calculated based on median values. For hypothesis testing, two-tailed, unpaired Mann-Whitney *U* tests were computed for the log-transformed data matrix if samples were independent. The obtained *p* values were adjusted for type I errors via the sequential goodness of fit metatest (SGoF) [42] or the false discovery rate [43] (FDR) procedure to yield true positive findings. The significance level *α* was set to 0.05 for the adjusted *p* and *q* values.

In order to estimate absolute lipid contents in experimental groups, deuterium-labeled lipids in the Lipidomix were utilized as class-specific one-point calibrants. Accordingly, concentrations of identified lipid species were calculated via the corresponding deuterated lipid for each sample. Peak areas were extracted via MultiQuant 3.0, as they more accurately describe the signal response (in the absence of interferences) and since chromatographic effects that lead to differences in peak width/height are more likely to be compensated. Moreover, manual control of the alignment as well as reintegration of peaks was conducted. However, one-point calibration is not the most accurate quantification method, mainly due to differences in the detector response between the target lipid and the surrogate calibrant [44] (deriving from non-co-elution in RP-LC and other factors) and because it is not certified that the calibration function intersects the *x*/*y*-origin (i.e., it has not been validated that the *y*-intercept can be neglected). The given concentrations should thus be treated as approximations of the true lipid levels.

All data processing and evaluations were executed with MS-DIAL [32], PeakView 2.2 (Sciex), MultiQuant 3.0 (Sciex), Excel 2019 (Microsoft, Redmond, WA, USA), Origin 2019 (OriginLab, Northampton, MA, USA), and R Studio 1.1.383 (R Foundation for Statistical Computing, Vienna, Austria).

## Results

### Lipidomic profile of in vivo dental plaque

Lipidomic profiling of biofilms was reported for a few model microorganisms only, e.g., *Candida albicans* biofilm [45, 46] or *Pseudomonas aeruginosa* biofilm [47]. In a recent study, GC-MS and UHPLC-MS/MS-based metabolomics profiling was performed on mineralized dental plaque samples which covered also lipids [48]. A detailed lipid profiling of dental plaque was carried out in a recent study in which fatty acid profiles have been determined by GC-MS [11]. However, in this approach, the association of the fatty acids to the head group and thus lipid class is lost. Therefore, we here employ a different approach: an in-depth lipidomics profiling of intact lipids by UHPLC separation hyphenated with high-resolution mass spectrometry. In this endeavor, the first critical step is the identification of the detected molecular features. By the described identification strategy, using MS-DIAL and lipid pattern recognition [36] (see ESM Table S15), structural assignment of about 5% of all detected molecular features in positive mode and around 2% in negative mode (Table 1) was achieved. In general, this typically led to an identification at the lipid species level (e.g., PC 34:1). If molecular lipid species-specific fragments (MLFs [24]) were detected in SWATH-MS/MS, additional information about the side chain composition of the primary lipid species for each identified lipid peak could be extracted. This way, the amount and ratio of lipids that carry hydrocarbon side chains with an even or odd carbon number could be examined. If no MLFs were present and the total number of carbons in hydrocarbon moieties was even, a classification into even- or odd-chain lipids was not possible. The results of this general lipid screening for both polarities are listed in Table 1.

**Table 1:** Lipidomic profiling of in vivo plaque and cultivated in vitro biofilm^a^

	Positive mode		Negative mode	
	In vivo plaque	In vitro biofilm	In vivo plaque	In vitro biofilm
Aligned features	4586	7564	1502	7144
Identified lipids*	243	350	33	157
Identification rate	5.3%	4.6%	2.2%	2.2%
Even-chain lipids	115	180	24	78
Odd-chain lipids	52	120	1	69
Even-chain/odd-chain lipid ratio	2.2	1.5	24	1.1
Lipids with unresolved side chain configuration	75	49	7	9
Annotated lipids without MS/MS verification	1060	1400	566	1640

^a^Data processing was executed with identical parameters but in separate runs for each experimental group

*With MS/MS spectra, lipids that were identified via more than one adduct were counted as one hit

It becomes evident that an adequate number of lipids could be identified in positive mode (ca. 5%), while the identification rate (2%) as well as the absolute number of aligned features is significantly lower in negative mode (note, identification means here structural annotation supported by MS/MS spectra). This can be explained by the overall decreased sensitivity and the inaccessibility of neutral lipids like TAGs and CEs in the negative polarity mode. In general, bacterial cell membranes are composed of lipids that may contain odd-chain fatty acids. However, for both modes, a clear trend toward even-chain lipids is observed (even-chain/odd-chain lipid ratio ≥ 2.2). It is also noteworthy that the number of additionally annotated lipids without verification by specific MS/MS fragments is relatively high (note, they may provide additional information but the structual assignments are less reliable). This may be due to insufficient fragmentation or more likely low sensitivity in the high-resolution SWATH-MS/MS mode.

Regarding the distribution of all identified lipid species according to their lipid classes, it can be seen that a majority of lipids belong to TAGs and PCs, which together cover 51.4% of all identified lipids (both polarities combined). Other frequently identified lipid classes were SMs (9.5%), ceramides (9.1%), PEs (8.3%), DAGs (7.5%), and LPCs (4.3%). Moreover, a small number of MGDGs and DGDGs were identified. These galactolipids are typically found in plant membranes [49] as secondary metabolites and were most probably incorporated into the plaque via food intake.

**Fig. 1:**
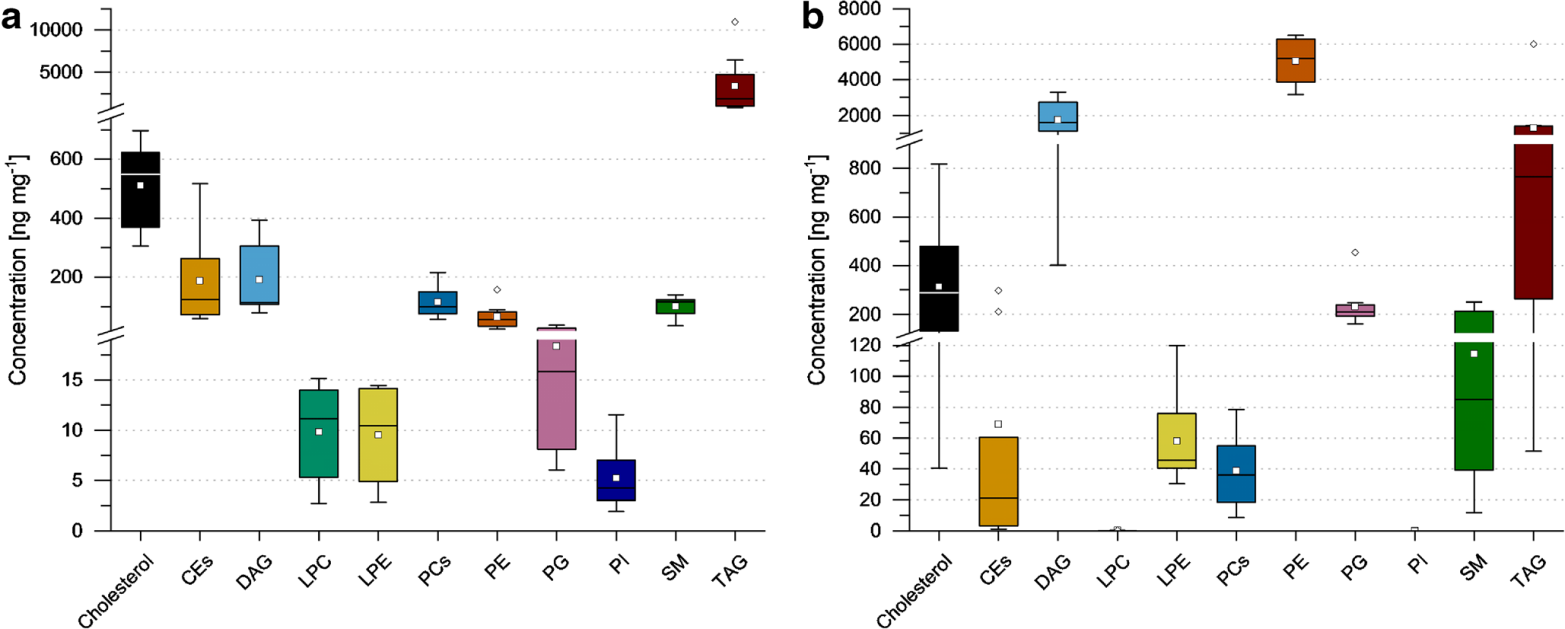
Box-whisker plots of concentrations for summed lipid class species in ng mg^−1^ (sum of both polarity modes; if lipids were detected in both modes the average value was considered). Ether-linked species were not considered as a distinct class and were calculated via the corresponding surrogate calibrant of the main lipid class. **a** In vivo plaque samples; **b** in vitro biofilm samples (here, no PI species were detected; LPC species were only detected at negligible levels < 2.0 ng mg^−1^)

Eventually, to estimate lipid class levels in the dried plaque samples as described in the previous section, concentrations of lipid species were approximated via one-point calibration using Lipidomix lipids as class-specific surrogate calibrants. Results for summed lipid class species are presented in Fig. 1a. Detailed results for (semi-)quantification in in vivo plaque are presented in ESM Table S5. Among all estimated lipid classes, TAGs are the most abundant (ca. 4 μg mg^−1^ plaque sample) and are followed by cholesterol (ca. 500 ng mL^−1^). CEs, DAGs, PCs, PEs, and SMs are largely in the same range and still far more abundant than PGs, LPCs, LPEs, and PIs (which are present in the range of 5–40 ng mg^−1^).

### Lipidomic profiling of cultivated in vitro biofilm (BF) and comparison with in vivo dental plaque (PL)

In vitro cultivated biofilm samples could be a readily available surrogate matrix for dental plaque which is limited in its quantities. In general, the number of molecular features detected in the cultivated in vitro biofilm samples greatly exceeded that of in vivo dental plaque samples, in particular in the negative ionization mode (partly due to a higher original sample weight, e.g., around 2 mg vs 0.8 to 1.6 mg in the case of plaque samples) (see Table 1). As identification rates for both experimental groups were similar in the respective modes, also the total number of identified lipids was significantly higher in BF samples (350 in positive and 157 in negative modes supported by matched MS/MS spectra). Regarding the even-chain/odd-chain lipid ratio in the BF group (≤ 1.5), the number of odd-chain lipid species is significantly higher than that in the PL group. This finding could imply enhanced bacterial activity in the cultivated medium [50].

A more detailed look into the distribution of lipids according to their lipid classes reveals significant differences compared with the PL samples. The percentage of DAGs and PEs is roughly doubled (13.3% and 14.6%, respectively), whereas that of PCs and LPCs was greatly reduced (5.8% and < 1%, respectively) in comparison with the PL samples. Moreover, elevated numbers of MGDGs (7.5%) and DGDGs (5.5%) are detected. The presence of galactolipids in the BF samples could originate from the TSB medium either by incorporation into the biofilm or as residuals from the TSB medium that were not efficiently removed in the washing step (mono- and digalactosyldiacylglycerols are typically membrane lipids that are found in plants).

The estimated concentration levels of summed lipid class species in BF samples (see Fig. 1b) underpin the above discussed shifts in lipid class frequencies. In particular, PEs show an up to 90-fold increase in concentration (medians) compared with the PL group and PE levels even outweigh that of TAGs. Moreover, the median concentration of DAGs is 15-fold increased. On the other hand, CE levels are up to 6-fold decreased compared with PL samples and also PCs and cholesterol show a slight decrease (2- to 3-fold) when comparing median values. More detailed results for (semi-)quantification of lipids in in vitro biofilm are provided in ESM Table S6.

**Fig. 2:**
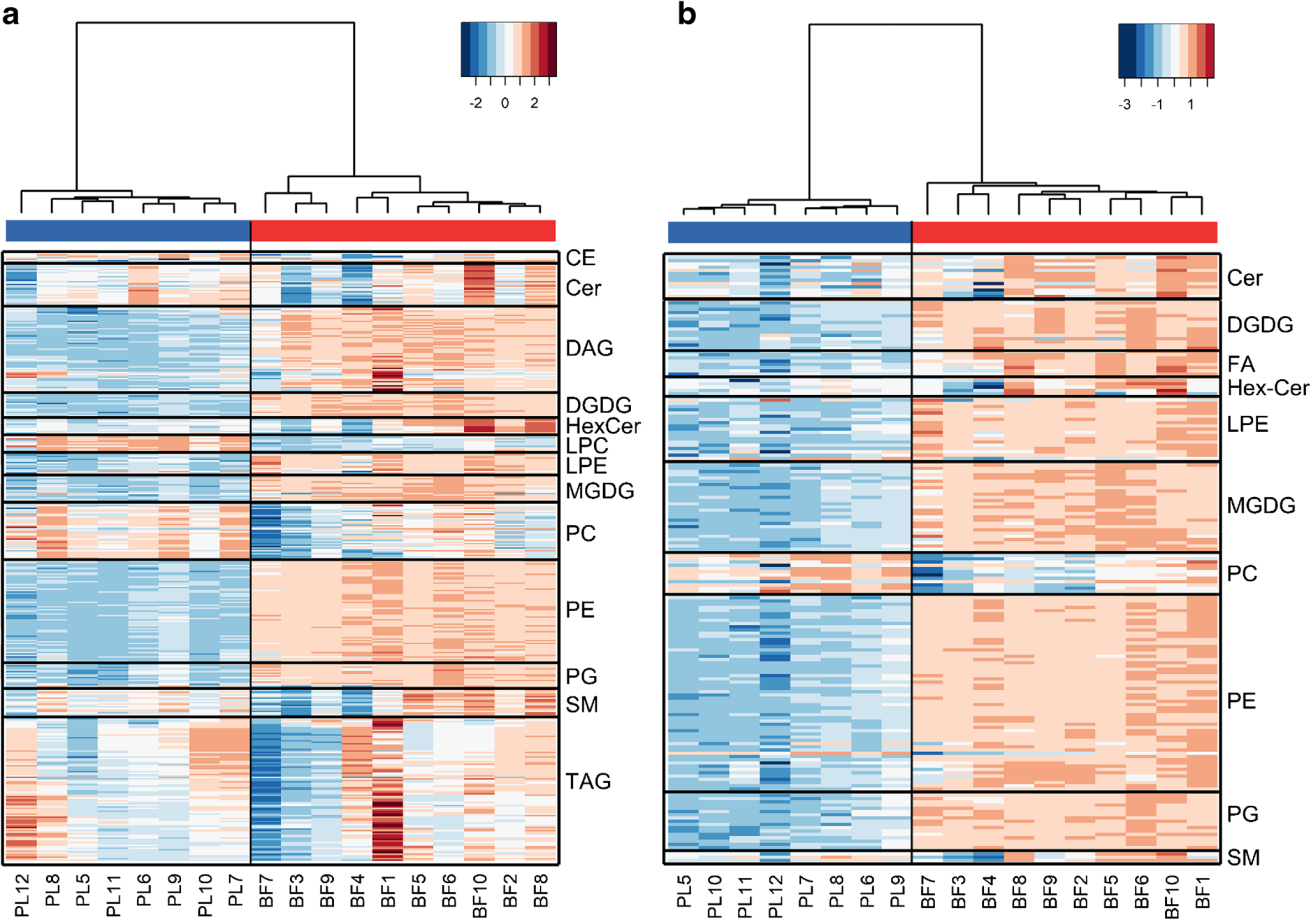
Heatmap for identified lipids in in vitro biofilm and in vivo plaque samples. Data is based on *z*-scores for the log-transformed data. Clustering was calculated using Ward’s method as agglomeration method and the Canberra method as distance method. **a** Positive mode data (raw height); **b** negative mode results (LOWESS normalized). *z*-Score is indicated by colors in legend

For a more comprehensive overview of differences between BF and PL samples, all samples of the analytical sequence (see ESM Table S2) were processed via MS-DIAL in a merged project to obtain a unified feature aligment file. After evaluation of several normalization methods [41], raw data (see ESM Fig. 1) and LOWESS-normalized data (see ESM Fig. S2) were chosen for further evaluation of positive mode and negative mode results, respectively, and heatmaps covering all identfied lipids were created (see Fig. 2). Here, trends in lipid abundances are clearly visualized. It can be further investigated if whole lipid classes show differences between experimental groups or if single lipid species or samples are responsible for an overall shift. The respective dendrograms above the heatmaps reveal an ideal clustering of experimental groups and display potential subgroups, e.g., in positive mode (Fig. 2a) the samples BF7, BF3, and BF9 are clustered due to diminished abundances of HexCers, PCs, SMs, and TAGs compared with the other BF samples.

In conclusion, most of the observed differences between BF and PL samples are caused by concentration shifts of all species among a lipid class (higher abundance in BF: ceramides, DAGs, DGDGs, HexCer, LPEs, MGDGs, PEs, and PGs; higher abundance in PL: LPCs and CEs). The class of PCs shows a mixed pattern, which indicates that only a few species are responsible for the elevated levels in PL samples (see Fig. 1). This finding will be further discussed in the following section in more detail. The heatmap for the positive mode dataset (Fig. 2a) also shows that TAG levels are sample specific and no clear, class-wide trend for a comparison can be claimed.

**Fig. 3:**
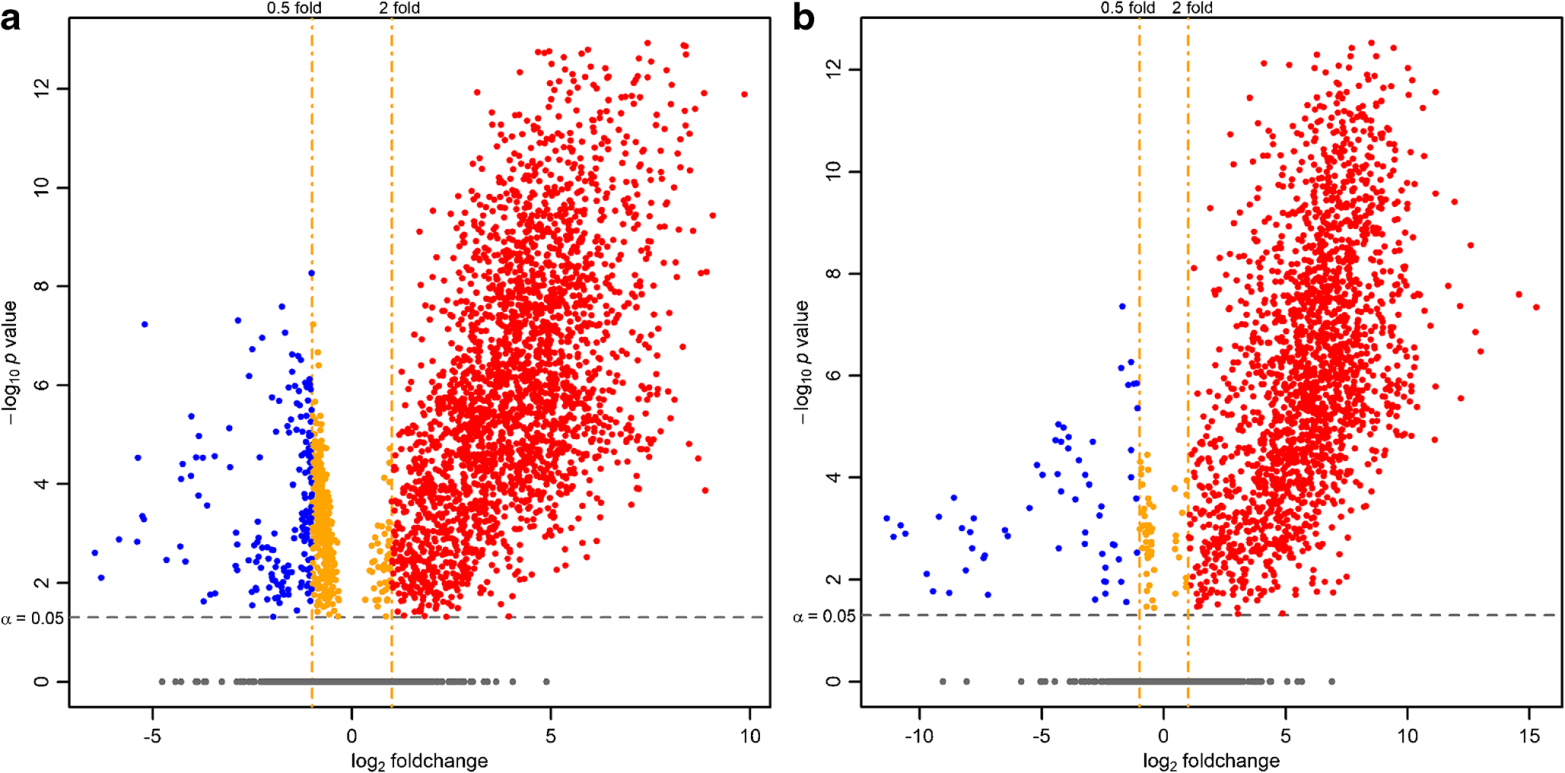
Volcano plots for detected features in in vitro biofilm (BF) versus in vivo plaque (PL) samples. Results are based on SGoF-adjusted *p* values (for both datasets more strict than FDR correction) and median fold changes. A significance level of *α* = 0.05 was chosen to evaluate true positive findings with significant differences between experimental groups. **a** Positive mode data (raw height), **b** negative mode results (LOWESS normalized)

After further statistical analysis (see data processing section) of the entire datasets including unknown features, *p* value histograms (ESM Fig. S3), principal component analysis (PCA, ESM Fig. S4), and relative log abundance (RLA) plots [51] (ESM Fig. S5) were computed. They all indicate the presence of many features with significantly different abundance between the two experimental groups, BF and PL, which are visualized in volcano plots (Fig. 3). Here, it can be readily derived that most of the aligned features are elevated in BF samples (positive mode: 4890 aligned features, 49.7% of all features show a > 2-fold higher abundance in BF samples with an SGoF-adjusted *p* value < 0.05; negative mode: 2687 aligned features, 69.6% of all features show a > 2-fold higher abundance in BF samples with an SGoF-adjusted *p* value < 0.05). Detailed tables that are summarizing significantly different identified lipids and their corresponding computed (adjusted) *p* values as well as fold changes are provided in the ESM (Table S7–S10).

**Fig. 4:**
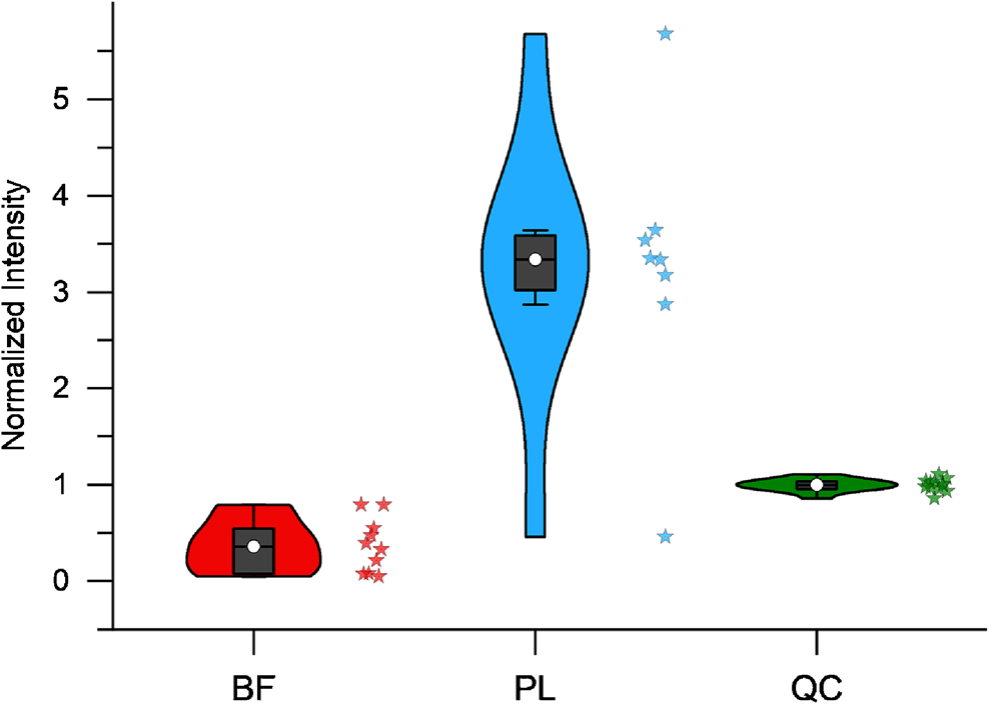
Violin plots of cholesterol-sulfate signal response (negative mode, LOWESS-normalized) in study groups. The stars next to the plots resemble the individual samples of the respective study group

Although most features are higher in abundance in BF samples, some distinct lipids or entire lipid classes (LPCs and HexHexCers) are specifically enhanced in the PL group. The most significantly elevated identified lipid in the PL group was cholesterol-sulfate (see Fig. 4). Like cholesterol, it is an integral part of eukaryotic cell membranes [52, 53] and the appearance of both analytes in the sample groups can be probably traced back to epithelial, buccal, or other cells present in the oral cavity or saliva. However, the (median) fold change of cholesterol-sulfate (0.11, *p* value = 3.8E−5) is much more pronounced than that of cholesterol (0.51, *p* value = 3.8E−4; peak height data) when comparing BF versus PL samples, which could imply a directed accumulation of cholesterol-sulfate in dental plaque.

Ultimately, the results indicate that cultivated in vitro biofilms generally contain higher lipid levels than dental plaque and that these two sample groups show vast differences as matrices. It can also be concluded that cultivation of saliva in TSB medium provides more vital growing conditions to bacteria than the environment in the oral cavity based on the enhanced formation of odd-chain lipid species.

### Investigation of even-chain PC species via SWATH-MS/MS

**Fig. 5:**
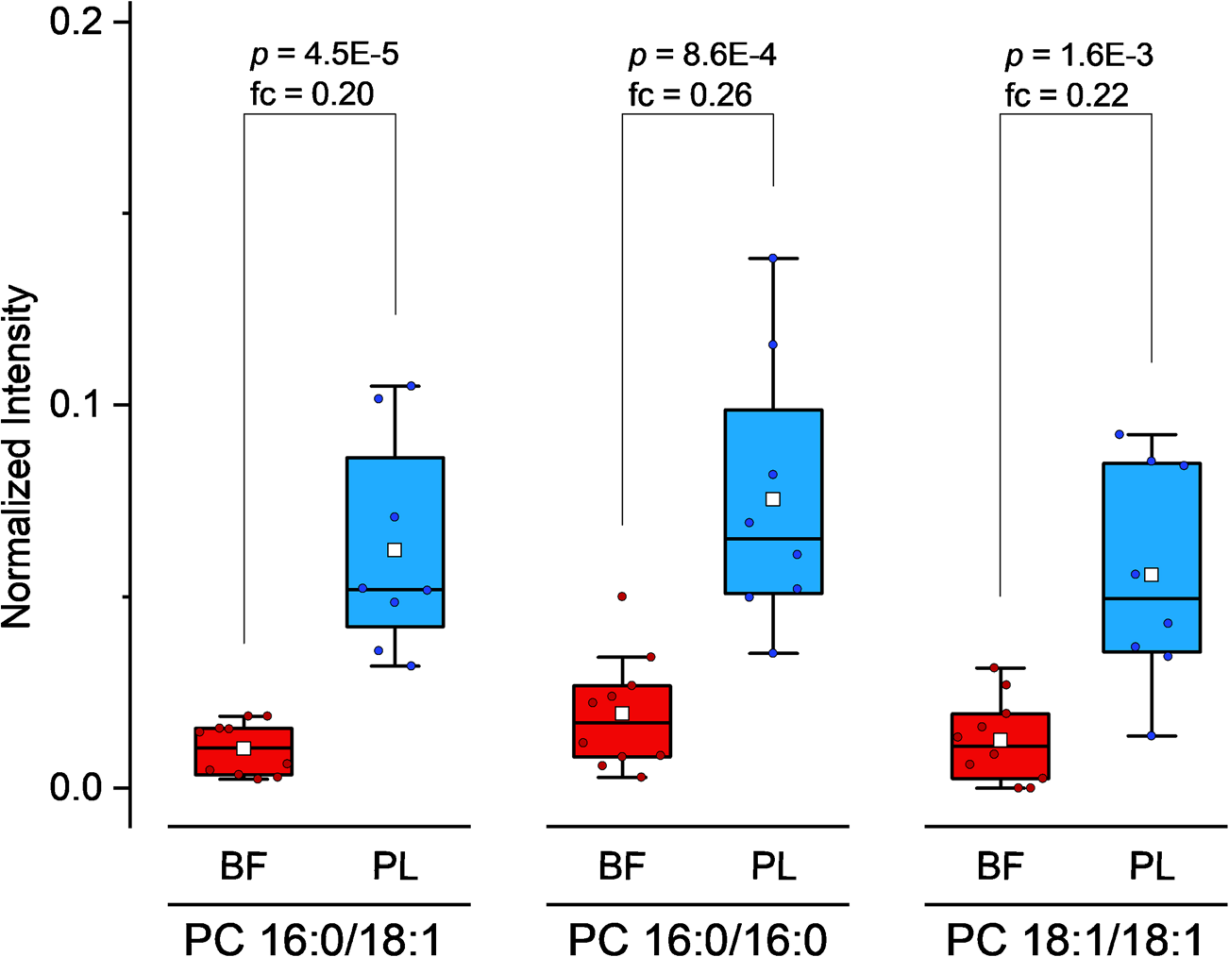
Box-whisker plots for the comparison of even-chain PC species between experimental groups via specific MLF intensities in SWATH-MS/MS. Data was normalized to sample weight (except for IS signals) and subsequently normalized to the corresponding IS intensity (deuterated MLF of 15:0–18:1 (d7) PC in SWATH-MS/MS). *p* values were calculated via Mann-Whitney *U* tests. Fold changes (fc) are based on median values

The observed mixed patterns of PCs in the shown heatmaps (Fig. 2) indicate that distinct PC species are elevated in BF and PL samples, respectively. Although the definite sn positions of the side chains for most PCs could not be resolved, statistical analysis results for the comparison of BF versus PL suggest that PCs with an even (total) number of hydrocarbons in the side chains are enhanced in the PL group (see ESM, Tables S7 and S9). However, as PC species with an identical sum formula but different side chain constitution may be insufficiently resolved chromatographically, TOF-MS results for even total side chain PCs (e.g., PC 32:0) potentially suffer from interference, which could be originating from odd-chain species (e.g., PC 15:0–17:0). To verify the hypothesis of elevated even-chain PCs in PL samples, the specific properties of SWATH acquisition can be beneficially utilized. As, in contrast to DDA techniques, comprehensive MS/MS spectra are recorded, SWATH is able to perform quantification with molecular lipid species-specific fragments (MLFs) on the MS/MS level. Accordingly, MLF-based extracted ion chromatograms (EICs) of three even-chain PCs (for which fatty acyls at sn1/2 were assigned by MS-DIAL; see ESM Table S7) were generated in the respective SWATH experiment at the retention time of the corresponding precursor. The quantitative results based on MS/MS of the interference-free MLFs confirm the assumption that even-chain PCs are of higher abundance in the PL group (Fig. 5).

### Comparison of in vivo plaque (PL) samples grown for different time periods

PL samples were divided into two groups, which covered samples that were collected after 24 h (PL 24 h) and after 72 h (PL 72 h) (see ESM Table S2) without tooth brushing. This comparison was devised in order to investigate possible compositional changes related to aging of the plaques. All relevant samples were processed via MS-DIAL with elevated intensity thresholds for feature detection (see ESM Table S4) to minimize the coverage of features that suffer from extensive noise. As the four samples per experimental group originated from one donor, the paired Mann-Whitney *U* test (Wilcoxon signed-rank tests) was used for hypothesis testing of the dependent samples. Investigation of normalization methods [41] again led to the usage of raw height data for positive mode (see ESM Fig. S6) and LOWESS-normalized data for negative mode (ESM Fig. S7). The histograms of the *p* value distributions (ESM Fig. S8) show that significant differences are present in the positive mode data matrix, whereas the almost evenly distributed *p* values for negative mode suggest no or only few true positive findings. However, for both datasets, no *q* values < 0.05 were computed after FDR adjustment. Only with non-conservative SGoF adjustment were *p* values below the significance level *α* of 0.05 obtained (see volcano plot in ESM Fig. S9). Detailed results for identified lipids with significantly different abundances in the distinct sample groups are listed in ESM Tables S11–S13. Overall, the data indicates a rather high similarity between the two experimental groups. Nevertheless, it can be observed that during in vivo dental plaque aging over 72 h LPCs, LPEs and in particular PEs are slightly accumulating (see ESM, Tables S11 and S13). On the other hand, PCs as well as TAGs are depleted in the aged samples (see ESM Table S12). Altogether, no radical changes in plaque lipid composition were observed during a period of 72 h and severe differences might only occur after further maturation of the plaque or follwing a shift from early- to late-colonizing bacteria, which would be expected after longer periods of undisturbed growth [1].

## Conclusion

The presented analytical workflow, consisting of a simple extraction protocol with broad lipid coverage, an effective RP-UHPLC lipid species separation method, a comprehensive QTOF-MS/MS acquisition strategy with SWATH, and several software tools for extensive data processing, was demonstrated to be an efficient tool for a detailed lipidomic characterization of biological samples. Besides the identification of TAGs, cholesterol, CEs, DAGs, and various phospholipids as the main lipid components in in vivo dental plaque, it could be shown that the early-stage aging of the plaque during 72 h does not drastically alter its lipidomic profile.

In addition, also the lipidome of in vitro biofilm, which was cultivated from saliva and is considered a potential surrogate candidate for plaque, was investigated. As comprehensively shown, the cultivated biofilm contains higher lipid contents for most of the observed lipid classes, with exceptionally large alterations in PE and LPE species. Moreover, the enhanced percentages of odd-chain lipids in the cultivated biofilms could be interpreted as an indicator of increased bacterial activity. Apart from these trends, LPCs, even-chain PCs, and cholesterol-derived species, like CEs and cholesterol-sulfate, were shown to be specifically elevated in dental plaque.

Regarding the observed differences in lipid composition (Fig. 1) and the pronounced differences in the majority of detected features (Fig. 3), it can be concluded that the investigated in vitro biofilm is not an appropriate surrogate matrix when the lipidome is targeted. However, since simple protein/buffer mixtures have been demonstrated to function as adequate surrogate matrices [54], the cultivated biofilm might yet be valid for matrix-matched calibration and preparation of QC samples in quantitative studies as a substitute for the scarce dental plaque, as long as its suitability for the targeted purpose can be demonstrated, e.g., by proving parallelism of calibration curves [55]. The proposed methodology can also be used in future studies to evaluate similarities and differences of in vivo plaque and other potential surrogate matrices or for optimization of the dental plaque surrogate matrix. Furthermore, the presented lipdomics profiling can be quite useful to monitor the biofilm phenotype in dental plaques to find biomarkers, which indicate when the microorganism colonization converts from microbial homeostasis under healthy state into disbiosys, i.e., shifts in composition of the biofilm, under diseased state (e.g., dental caries and periodontal diseases).

## Electronic supplementary material

ESM 1 (PDF 2032 kb)
