# Supplementary material for: Lipidomic profiling of non-mineralized dental plaque and biofilm by untargeted UHPLC-QTOF-MS/MS and SWATH acquisition
Source: Anal Bioanal Chem. 2020 Jan 15;412(10):2303–14. doi: 10.1007/s00216-019-02364-2 (PMC7118048; doi:10.1007/s00216-019-02364-2)
Supplement: Supplementary file 1 — (PDF 2032 kb) [file 216_2019_2364_MOESM1_ESM.pdf]

Analytical and Bioanalytical Chemistry

Electronic Supplementary Material

Lipidomic profiling of non-mineralized dental plaque and biofilm by untargeted UHPLC-QTOF-MS/MS and SWATH acquisition

Bernhard Drotleff, Simon R. Roth, Kerstin Henkel, Carlos Calderón, Jörg Schlotterbeck, Merja A. Neukamm, Michael Lämmerhofer

Table S1 Deuterated lipid standards (Lipidomix) used for normalization and one-point calibration

Compound name	Sum formula	Conc. [ng mL^{-1}]	t_R [min]	Positive polarity mode		Negative polarity mode	
				Adduct type	TOF extraction mass	Adduct type	TOF extraction mass
18:1(d7) LPC	$C_{26}H_{45}D_7NO_7P$	252.5	1.52 ± 0.01	$[M+H]^+$	529.3994	$[M+HCOO]^-$	573.3903
18:1(d7) LPE	$C_{23}H_{39}D_7NO_7P$	52.5	1.56 ± 0.00	$[M+H]^+$	487.3524	$[M-H]^-$	485.3379
15:0-18:1(d7) PI	$C_{42}H_{72}D_7O_{13}P$	88.3	4.27 ± 0.02	$[M+NH_4]^+$	847.6036	$[M-H]^-$	828.5625
15:0-18:1(d7) PG	$C_{39}H_{68}D_7O_{10}P$	279.8	4.43 ± 0.04	$[M+NH_4]^+$	759.5876	$[M-H]^-$	740.5465
d18:1-18:1(d9) SM	$C_{41}H_{72}D_9N_2O_6P$	305.9	4.66 ± 0.00	$[M+H]^+$	738.6470	$[M+HCOO]^-$	782.6379
Cholesterol(d7)	$C_{27}H_{39}D_7O$	974.3	4.68 ± 0.01	$[M-H_2O+H]^+$	376.3955	-	-
15:0-18:1(d7) PC	$C_{41}H_{73}D_7NO_8P$	1591.1	5.01 ± 0.01	$[M+H]^+$	753.6134	$[M+HCOO]^-$	797.6043
15:0-18:1(d7) PE	$C_{38}H_{67}D_7NO_8P$	56.4	5.19 ± 0.00	$[M+H]^+$	711.5664	$[M-H]^-$	709.5519
15:0-18:1(d7) DAG	$C_{36}H_{61}D_7O_5$	93.1	6.40 ± 0.01	$[M+NH_4]^+$	605.5844	$[M+HCOO]^-$	632.5488
15:0-18:1(d7)-15:0 TAG	$C_{51}H_{89}D_7O_6$	567.3	10.32 ± 0.01	$[M+NH_4]^+$	829.7985	-	-
18:1(d7) CE	$C_{45}H_{71}D_7O_2$	3525.7	10.74 ± 0.04	$[M+NH_4]^+$	675.6780	-	-

Given concentrations reflect the final concentrations in the analyzed supernatant. In addition, 15:0-18:1(d7) phosphatidic acid, 18:1(d7) monoacylglycerol, and 15:0-18:1(d7) phosphatidylserine are covered in the Lipidomix but were not considered as no species of these classes were detected

Table S2 Sample description and analytical properties

Sample	Sample description	Analytical order	Sample dry weight[mg]
BF1	*in vitro* biofilm (BF)	4	2.06
BF2	*in vitro* biofilm (BF)	13	2.05
BF3	*in vitro* biofilm (BF)	26	2.07
BF4	*in vitro* biofilm (BF)	6	1.99
BF5	*in vitro* biofilm (BF)	24	2.03
BF6	*in vitro* biofilm (BF)	14	2.05
BF7	*in vitro* biofilm (BF)	16	2.05
BF8	*in vitro* biofilm (BF)	21	2.03
BF9	*in vitro* biofilm (BF)	19	2.04
BF10	*in vitro* biofilm (BF)	11	1.99
BF11*	*in vitro* biofilm (pool BF1 - 5)	10	1.96
BF12*	*in vitro* biofilm (pool BF6 - 10)	9	1.92
PL5	*in vivo* dental plaque (PL, 24 h growth)	18	1.03
PL6	*in vivo* dental plaque (PL, 72 h growth)	7	1.57
PL7	*in vivo* dental plaque (PL, 24 h growth)	28	1.15
PL8	*in vivo* dental plaque (PL, 24 h growth)	5	1.11
PL9	*in vivo* dental plaque (PL, 72 h growth)	29	1.23
PL10	*in vivo* dental plaque (PL, 72 h growth)	25	1.25
PL11	*in vivo* dental plaque (PL, 72 h growth)	22	1.45
PL12	*in vivo* dental plaque (PL, 24 h growth)	17	0.80
QC1	QC (pool of all samples)	1	-
QC2	QC (pool of all samples)	2	-
QC3	QC (pool of all samples)	3	-
QC4	QC (pool of all samples)	8	-
QC5	QC (pool of all samples)	12	-
QC6	QC (pool of all samples)	15	-
QC7	QC (pool of all samples)	20	-
QC8	QC (pool of all samples)	23	-
QC9	QC (pool of all samples)	27	-
QC10	QC (pool of all samples)	30	-
QC11	QC (pool of all samples)	31	-
QC12	QC (pool of all samples)	32	-

Samples were normalized to sample weight by dividing feature signal intensities through the respective weight. *BF11 and BF12 were pooled *in vitro* biofilm samples of residual saliva. Ultimately, these pools were not considered for data evaluation

Table S3 MS experiment design

Experiment	*m/z* range	
	**Positive mode**	**Negative mode**
TOF-MS	50 - 1250	50 - 1250
SWATH-MS/MS 1	50 - 251.7	50 - 137.4
SWATH-MS/MS 2	250.7 - 287.8	136.4 - 198.5
SWATH-MS/MS 3	286.8 - 326.8	197.5 - 255.7
SWATH-MS/MS 4	325.8 - 362.3	254.7 - 301.6
SWATH-MS/MS 5	361.3 - 398.8	300.6 - 359.4
SWATH-MS/MS 6	397.8 - 439.4	358.4 - 385.4
SWATH-MS/MS 7	438.4 - 508.8	384.4 - 437.4
SWATH-MS/MS 8	507.8 - 560	436.4 - 491.9
SWATH-MS/MS 9	559 - 590.5	490.9 - 573.3
SWATH-MS/MS 10	589.5 - 620	572.3 - 625
SWATH-MS/MS 11	619 - 640	624 - 659.9
SWATH-MS/MS 12	639 - 662	658.9 - 688
SWATH-MS/MS 13	661 - 683.5	687 - 713.1
SWATH-MS/MS 14	682.5 - 706.1	712.1 - 730.8
SWATH-MS/MS 15	705.1 - 733.1	729.8 - 751.9
SWATH-MS/MS 16	732.1 - 767	750.9 - 775
SWATH-MS/MS 17	766 - 826.2	774 - 805.4
SWATH-MS/MS 18	825.2 - 887.7	804.4 - 844.9
SWATH-MS/MS 19	886.7 - 925.3	843.9 - 909.6
SWATH-MS/MS 20	924.3 - 1250	908.6 - 1250

Table S4 MS-DIAL processing parameters

Parameter		Setting	
		**Positive mode**	**Negative mode**
Data collection range		0.5 – 13.0 min	0.5 – 13.0 min
Mass range		50 – 1250	50 – 1250
MS tolerance		0.01 Da	0.01 Da
MS/MS tolerance		0.025 Da	0.025 Da
Smoothing level		2	2
Minimum peak width[data points]		5	5
Minimum peak height for feature detection [cps]	lipid profiling of *in vivo* plaque	100	100
	lipid profiling of *in vitro* biofilm	100	100
	in vitro vs. *in vivo* samples	750	500
	comparison of formation times	500	250
ID retention time tolerance		1.0 min	1.0 min
ID score		80%	80%
Alignment t_R tolerance		0.15 min	0.15 min
Alignment MS tolerance		0.015 Da	0.015 Da

Table S5 Identified lipids and estimated concentration levels in *in vivo* plaque samples

Identified lipid; primary species; [adduct type]	Sum formula	t_R [min]	Measured precursor *m/z*	Calculated precursor *m/z*	Mean calculated concentration [ng*mg^{-1}] pos. mode	Mean calculated concentration [ng*mg^{-1}] neg. mode	ID in both polarity modes
CE 18:1; $[M+NH_4]^+$	$C_{45}H_{78}O_2$	10.79	668.6395	668.6345	14.3 ± 7.3	-	-
CE 18:2; $[M+NH_4]^+$	$C_{45}H_{76}O_2$	10.31	666.6197	666.6189	76.7 ± 82.2	-	-
CE 19:2; $[M+NH_4]^+$	$C_{46}H_{78}O_2$	10.73	680.6274	680.6345	39.2 ± 10.7	-	-
CE 20:4; $[M+NH_4]^+$	$C_{47}H_{76}O_2$	10.04	690.6265	690.6189	56.9 ± 73.6	-	-
Cer-NDS d34:0; Cer-NDS d18:0/16:0; $[M+H]^+$	$C_{34}H_{69}NO_3$	5.67	540.5422	540.5355	-	-	-
Cer-NDS d36:0; Cer-NDS d18:0/18:0; $[M+H]^+$	$C_{36}H_{73}NO_3$	6.38	568.5665	568.5668	-	-	-
Cer-NDS d40:0; Cer-NDS d18:0/22:0; $[M+H]^+$	$C_{40}H_{81}NO_3$	7.75	624.6334	624.6294	-	-	-
Cer-NDS d42:0; Cer-NDS d18:0/24:0; $[M+H]^+$	$C_{42}H_{85}NO_3$	8.39	652.6659	652.6607	-	-	-
Cer-NDS d44:0; Cer-NDS d18:0/26:0; $[M+H]^+$	$C_{44}H_{89}NO_3$	8.96	680.6953	680.6920	-	-	-
Cer-NS d33:1; Cer-NS d18:1/15:0; $[M+H]^+$	$C_{33}H_{65}NO_3$	5.10	524.5062	524.5042	-	-	-
Cer-NS d34:1; Cer-NS d18:1/16:0; $[M+H]^+$	$C_{34}H_{67}NO_3$	5.44	538.5241	538.5199	-	-	-
Cer-NS d34:2; Cer-NS d18:1/16:1; $[M+H]^+$	$C_{34}H_{65}NO_3$	5.13	536.5032	536.5042	-	-	-
Cer-NS d35:1; Cer-NS d18:1/17:0; $[M+H]^+$	$C_{35}H_{69}NO_3$	5.79	552.5313	552.5355	-	-	-
Cer-NS d36:1; Cer-NS d18:1/18:0; $[M+H]^+$	$C_{36}H_{71}NO_3$	6.14	566.5538	566.5512	-	-	-
Cer-NS d36:4; Cer-NS d18:1/18:3; $[M+H]^+$	$C_{36}H_{65}NO_3$	5.44	560.5035	560.5042	-	-	-
Cer-NS d38:1; Cer-NS d18:1/20:0; $[M+H]^+$	$C_{38}H_{75}NO_3$	6.84	594.5858	594.5825	-	-	-
Cer-NS d38:4; Cer-NS d18:1/20:3; $[M+H]^+$	$C_{38}H_{69}NO_3$	6.14	588.5378	588.5355	-	-	-
Cer-NS d39:1; Cer-NS d17:1/22:0; $[M+H]^+$	$C_{39}H_{77}NO_3$	7.18	608.6068	608.5981	-	-	-
Cer-NS d40:1; Cer-NS d18:1/22:0; $[M+H]^+$	$C_{40}H_{79}NO_3$	7.52	622.6152	622.6138	-		-
Cer-NS d40:4; Cer-NS d18:1/22:3; $[M+H]^+$	$C_{40}H_{73}NO_3$	6.83	616.5660	616.5668	-	-	-
Cer-NS d41:1; Cer-NS d17:1/24:0; $[M+H]^+$	$C_{41}H_{81}NO_3$	7.85	636.6335	636.6294	-	-	-
Cer-NS d42:1; Cer-NS d18:1/24:0; $[M+H]^+$	$C_{42}H_{83}NO_3$	8.17	650.6460	650.6451	-	-	-
Cer-NS d42:4; Cer-NS d18:1/24:3; $[M+H]^+$	$C_{42}H_{77}NO_3$	7.51	644.5979	644.5981	-	-	-
Cer-NS d43:1; Cer-NS d18:1/25:0; $[M+H]^+$	$C_{43}H_{85}NO_3$	8.47	664.6619	664.6607	-	-	-
Cer-NS d44:1; Cer-NS d18:1/26:0; $[M+H]^+$	$C_{44}H_{87}NO_3$	8.77	678.6802	678.6764	-		-
Cer-NS d45:1; Cer-NS d18:1/27:0; $[M+H]^+$	$C_{45}H_{89}NO_3$	9.04	692.6926	692.6920	-	-	-
Cer-NS d46:1; Cer-NS d18:1/28:0; $[M+H]^+$	$C_{46}H_{91}NO_3$	9.32	706.7094	706.7077	-	-	-
Cholesterol; $[M-H_2O+H]^+$	$C_{27}H_{46}O$	4.66	369.3512	369.3516	510.5 ± 147.1	-	-

Cholesterol-sulfate; [M-H]$^-$	$C_{27}H_{46}O_4S$	2.95	465.3035	465.3044	-	-	-
DAG 25:1e; DAG 14:1e/11:0; [M+NH$_4$]$^+$	$C_{28}H_{54}O_4$	4.05	472.4355	472.4366	0.44 ± 0.34	-	-
DAG 26:0; DAG 10:0-16:0; [M+NH$_4$]$^+$	$C_{29}H_{56}O_5$	4.68	502.4448	502.4471	3.83 ± 4.36	-	-
DAG 28:0; DAG 12:0-16:0; [M+NH$_4$]$^+$	$C_{31}H_{60}O_5$	5.32	530.4770	530.4784	1.77 ± 1.57	-	-
DAG 28:1; DAG 10:0-18:1; [M+NH$_4$]$^+$	$C_{31}H_{58}O_5$	4.75	528.4602	528.4628	1.49 ± 1.87	-	-
DAG 30:0; DAG 14:0-16:0; [M+NH$_4$]$^+$	$C_{33}H_{64}O_5$	6.00	558.5085	558.5097	3.83 ± 2.99	-	-
DAG 31:0; DAG 15:0-16:0; [M+NH$_4$]$^+$	$C_{34}H_{66}O_5$	6.24	572.5280	572.5254	0.91 ± 0.53	-	-
DAG 32:0; DAG 16:0-16:0; [M+NH$_4$]$^+$	$C_{35}H_{68}O_5$	6.70	586.5376	586.5410	8.13 ± 4.45	-	-
DAG 32:1; DAG 16:0-16:1; [M+NH$_4$]$^+$	$C_{35}H_{66}O_5$	6.07	584.5239	584.5254	6.34 ± 4	-	-
DAG 34:0; DAG 16:0-18:0; [M+NH$_4$]$^+$	$C_{37}H_{72}O_5$	7.36	614.5710	614.5723	6.35 ± 2.48	-	-
DAG 34:1; DAG 16:0-18:1; [M+NH$_4$]$^+$	$C_{37}H_{70}O_5$	6.74	612.5590	612.5567	28.9 ± 13.3	-	-
DAG 34:2; DAG 16:0-18:2; [M+NH$_4$]$^+$	$C_{37}H_{68}O_5$	6.20	610.5415	610.5410	14.2 ± 12.2	-	-
DAG 36:0; DAG 18:0-18:0; [M+NH$_4$]$^+$	$C_{39}H_{76}O_5$	8.01	642.6008	642.6036	4.58 ± 3.4	-	-
DAG 36:1; DAG 18:0-18:1; [M+NH$_4$]$^+$	$C_{39}H_{74}O_5$	7.42	640.5863	640.5880	11.5 ± 7.5	-	-
DAG 36:2; DAG 18:1-18:1; [M+NH$_4$]$^+$	$C_{39}H_{72}O_5$	6.81	638.5745	638.5723	36.8 ± 41.4	-	-
DAG 36:3; DAG 18:1-18:2; [M+NH$_4$]$^+$	$C_{39}H_{70}O_5$	6.26	636.5588	636.5567	31.6 ± 38	-	-
DAG 36:4; DAG 18:2-18:2; [M+NH$_4$]$^+$	$C_{39}H_{68}O_5$	5.73	634.5406	634.5410	22.5 ± 27.4	-	-
DAG 39:2e; DAG 18:2e/21:0; [M+NH$_4$]$^+$	$C_{42}H_{80}O_4$	7.86	666.6451	666.6400	3.48 ± 2.04	-	-
DAG 41:2e; DAG 18:2e/23:0; [M+NH$_4$]$^+$	$C_{44}H_{84}O_4$	8.47	694.6726	694.6713	3.43 ± 2.36	-	-
DAG 45:7e; DAG 17:0e/28:7; [M+NH$_4$]$^+$	$C_{48}H_{82}O_4$	7.26	740.6431	740.6556	0.51 ± 0.27	-	-
DGDG 30:0; DGDG 14:0-16:0; [M+NH$_4$]$^+$	$C_{45}H_{84}O_{15}$	4.67	882.6169	882.6154	-	-	-
DGDG 32:1; DGDG 16:0-16:1; [M+NH$_4$]$^+$	$C_{47}H_{86}O_{15}$	4.70	908.6263	908.6310	-	-	-
HexHexCer d42:1; HexHexCer d18:1/24:0; [M+H]$^+$	$C_{54}H_{103}NO_{13}$	7.20	974.7528	974.7507	-	-	-
HexHexCer d42:2; HexHexCer d18:1/24:1; [M+H]$^+$	$C_{54}H_{101}NO_{13}$	6.54	972.7360	972.7351	-	-	-
LPC 16:0; [M+H]$^+$	$C_{24}H_{50}NO_7P$	1.28	496.3427	496.3403	0.93 ± 0.43	-	-
LPC 16:1; [M+H]$^+$	$C_{24}H_{48}NO_7P$	0.97	494.3275	494.3246	0.18 ± 0.13	-	-
LPC 16:1e; [M+H]$^+$	$C_{24}H_{50}NO_6P$	1.58	480.3440	480.3454	0.42 ± 0.23	-	-
LPC 18:0; [M+H]$^+$	$C_{26}H_{54}NO_7P$	2.09	524.3699	524.3716	1.16 ± 0.47	-	-
LPC 18:1; [M+H]$^+$	$C_{26}H_{52}NO_7P$	1.40	522.3560	522.3559	2.54 ± 1.61	-	-
LPC 18:2; [M+H]$^+$	$C_{26}H_{50}NO_7P$	1.09	520.3403	520.3403	1.35 ± 1.92	-	-
LPC 20:0e; [M+H]$^+$	$C_{28}H_{60}NO_6P$	3.12	538.4218	538.4236	0.47 ± 0.29	-	-
LPC 20:4; [M+H]$^+$	$C_{28}H_{50}NO_7P$	1.06	544.3384	544.3403	0.12 ± 0.11	-	-
LPC 22:1e; [M+H]$^+$	$C_{30}H_{62}NO_6P$	3.16	564.4395	564.4393	0.97 ± 0.58	-	-
LPC 24:1e; [M+H]$^+$	$C_{32}H_{66}NO_6P$	3.63	592.4710	592.4706	1.45 ± 0.88	-	-
LPC 26:0; [M+H]$^+$	$C_{34}H_{70}NO_7P$	4.50	636.4937	636.4968	0.30 ± 0.05	-	-

LPE 16:0; $[M+H]^+$	$C_{21}H_{44}NO_7P$	1.42	454.2929	454.2933	0.77 ± 0.68	0.71 ± 0.69	✓
LPE 16:1e; $[M+H]^+$	$C_{21}H_{44}NO_6P$	1.63	438.2985	438.2984	1.97 ± 1.10	-	-
LPE 18:0; $[M+H]^+$	$C_{23}H_{48}NO_7P$	2.15	482.3243	482.3246	0.80 ± 0.49	0.75 ± 0.41	✓
LPE 18:1; $[M+H]^+$	$C_{23}H_{46}NO_7P$	1.44	480.3104	480.3090	0.88 ± 0.38	0.79 ± 0.43	✓
LPE 18:1e; $[M+H]^+$	$C_{23}H_{48}NO_6P$	2.44	466.3304	466.3297	4.01 ± 2.49	4.40 ± 2.72	✓
LPE 20:4; $[M-H]^-$	$C_{25}H_{44}NO_7P$	1.10	500.2748	500.2783	-	0.22 ± 0.13	-
LPE 22:2e; $[M+H]^+$	$C_{27}H_{54}NO_6P$	3.17	520.3790	520.3767	0.57 ± 0.28	-	-
LPE 24:2e; $[M+H]^+$	$C_{29}H_{58}NO_6P$	3.65	548.4068	548.4080	0.53 ± 0.31	-	-
MGDG 32:0; MGDG 16:0-16:0; $[M+HCOO]^-$	$C_{41}H_{78}O_{10}$	5.36	775.5482	775.5577	-	-	-
MGDG 34:1; MGDG 16:0-18:1; $[M+HCOO]^-$	$C_{43}H_{80}O_{10}$	5.87	801.5682	801.5734	-	-	-
PC 30:0; $[M+H]^+$	$C_{38}H_{76}NO_8P$	4.65	706.5388	706.5387	1.63 ± 0.51	-	-
PC 30:0e; $[M+H]^+$	$C_{38}H_{78}NO_7P$	5.01	692.5590	692.5594	0.57 ± 0.37	-	-
PC 31:0; $[M+H]^+$	$C_{39}H_{78}NO_8P$	4.94	720.5540	720.5543	0.55 ± 0.32	-	-
PC 31:0e; $[M+H]^+$	$C_{39}H_{80}NO_7P$	5.34	706.5773	706.5750	0.28 ± 0.17	-	-
PC 32:0; PC 16:0-16:0; $[M+H]^+$	$C_{40}H_{80}NO_8P$	5.26	734.5690	734.5699	8.01 ± 3.83	8.04 ± 3.70	✓
PC 32:0e; $[M+H]^+$	$C_{40}H_{82}NO_7P$	5.67	720.5922	720.5907	6.85 ± 3.70	5.78 ± 3.22	✓
PC 32:1; $[M+H]^+$	$C_{40}H_{78}NO_8P$	4.72	732.5584	732.5543	3.46 ± 1.33	-	-
PC 32:1e; $[M+H]^+$	$C_{40}H_{80}NO_7P$	5.11	718.5736	718.5750	0.70 ± 0.38	-	-
PC 32:2; $[M+H]^+$	$C_{40}H_{76}NO_8P$	4.25	730.5383	730.5387	0.38 ± 0.53	-	-
PC 33:1; PC 15:0-18:1; $[M+H]^+$	$C_{41}H_{80}NO_8P$	5.02	746.5739	746.5699	1.15 ± 0.42	-	-
PC 34:0; $[M+H]^+$	$C_{42}H_{84}NO_8P$	5.93	762.6024	762.6012	1.88 ± 0.89	1.71 ± 0.93	✓
PC 34:0e; $[M+H]^+$	$C_{42}H_{86}NO_7P$	6.34	748.6256	748.6220	1.43 ± 0.79	-	-
PC 34:1; PC 16:0-18:1; $[M+H]^+$	$C_{42}H_{82}NO_8P$	5.34	760.5861	760.5856	24.1 ± 11.9	24.0 ± 11.6	✓
PC 34:1e; $[M+H]^+$	$C_{42}H_{84}NO_7P$	5.73	746.6086	746.6063	8.16 ± 4.27	-	-
PC 34:2; $[M+H]^+$	$C_{42}H_{80}NO_8P$	4.85	758.5661	758.5699	6.88 ± 4.72	6.59 ± 4.84	✓
PC 34:2e; $[M+H]^+$	$C_{42}H_{82}NO_7P$	5.23	744.5893	744.5907	1.84 ± 1.06	-	-
PC 34:3; $[M+H]^+$	$C_{42}H_{78}NO_8P$	4.36	756.5524	756.5543	0.85 ± 1.50	-	-
PC 34:4e; $[M+H]^+$	$C_{42}H_{78}NO_7P$	4.67	740.5411	740.5594	0.16 ± 0.09	-	-
PC 35:1; $[M+H]^+$	$C_{43}H_{84}NO_8P$	5.65	774.5990	774.6012	0.71 ± 0.45	-	-
PC 35:2; $[M+H]^+$	$C_{43}H_{82}NO_8P$	5.09	772.5905	772.5856	0.46 ± 0.33	-	-
PC 36:0e; $[M+H]^+$	$C_{44}H_{90}NO_7P$	7.02	776.6555	776.6533	0.26 ± 0.15	-	-
PC 36:1; $[M+H]^+$	$C_{44}H_{86}NO_8P$	6.00	788.6210	788.6169	10.1 ± 4.9	9.07 ± 4.58	✓
PC 36:1e; $[M+H]^+$	$C_{44}H_{88}NO_7P$	6.41	774.6422	774.6376	3.36 ± 20	-	-
PC 36:2; PC 18:1-18:1; $[M+H]^+$	$C_{44}H_{84}NO_8P$	5.41	786.6022	786.6012	10.8 ± 6.6	10.3 ± 5.8	✓
PC 36:2e; $[M+H]^+$	$C_{44}H_{86}NO_7P$	5.79	772.6197	772.6220	4.18 ± 2.94	-	-

PC 36:3; $[M+H]^+$	$C_{44}H_{82}NO_8P$	4.92	784.5867	784.5856	4.23 ± 4.61	3.71 ± 4.61	✓
PC 36:3e; $[M+H]^+$	$C_{44}H_{84}NO_7P$	5.28	770.6056	770.6063	1.40 ± 0.79	0.97 ± 0.73	✓
PC 36:4e; $[M+H]^+$	$C_{44}H_{82}NO_7P$	5.13	768.5929	768.5907	1.33 ± 0.87	-	-
PC 38:1; $[M+H]^+$	$C_{46}H_{90}NO_8P$	6.60	816.6573	816.6482	0.22 ± 0.10	-	-
PC 38:1e; $[M+H]^+$	$C_{46}H_{92}NO_7P$	7.04	802.6679	802.6689	1.02 ± 0.60	-	-
PC 38:2; $[M+H]^+$	$C_{46}H_{88}NO_8P$	6.02	814.6332	814.6325	0.81 ± 0.45	0.49 ± 0.37	✓
PC 38:2e; $[M+H]^+$	$C_{46}H_{90}NO_7P$	6.44	800.6641	800.6533	0.95 ± 0.66	-	-
PC 38:3; $[M+H]^+$	$C_{46}H_{86}NO_8P$	5.65	812.6176	812.6169	0.28 ± 0.16	-	-
PC 38:4; $[M+HCOO]^-$	$C_{46}H_{84}NO_8P$	5.43	854.5866	854.5917	-	0.42 ± 0.42	-
PC 38:4e; $[M+H]^+$	$C_{46}H_{86}NO_7P$	5.78	796.6263	796.6220	1.09 ± 0.79	-	-
PC 38:5; $[M+H]^+$	$C_{46}H_{82}NO_8P$	4.81	808.5938	808.5856	0.41 ± 0.32	-	-
PC 38:5e; $[M+H]^+$	$C_{46}H_{84}NO_7P$	5.17	794.6076	794.6063	1.25 ± 0.89	-	-
PC 38:6; $[M+H]^+$	$C_{46}H_{80}NO_8P$	4.38	806.5674	806.5699	0.08 ± 0.06	-	-
PC 40:1e; $[M+H]^+$	$C_{48}H_{96}NO_7P$	7.63	830.6990	830.7002	0.66 ± 0.41	-	-
PC 40:2e; $[M+H]^+$	$C_{48}H_{94}NO_7P$	7.06	828.6843	828.6846	0.76 ± 0.44	-	-
PC 40:3e; $[M+H]^+$	$C_{48}H_{92}NO_7P$	6.55	826.6676	826.6689	0.62 ± 0.38	-	-
PC 40:4e; $[M+H]^+$	$C_{48}H_{90}NO_7P$	6.45	824.6555	824.6533	0.25 ± 0.15	-	-
PC 40:5e; $[M+H]^+$	$C_{48}H_{88}NO_7P$	5.63	822.6381	822.6376	0.32 ± 0.21	-	-
PC 42:2e; $[M+H]^+$	$C_{50}H_{98}NO_7P$	7.68	856.7202	856.7159	0.82 ± 0.48	-	-
PC 42:3e; $[M+H]^+$	$C_{50}H_{96}NO_7P$	7.17	854.7003	854.7002	0.82 ± 0.50	-	-
PC 42:5e; $[M+H]^+$	$C_{50}H_{92}NO_7P$	6.44	850.6746	850.6689	0.38 ± 0.25	-	-
PC 44:5e; $[M+H]^+$	$C_{52}H_{96}NO_7P$	7.07	878.7053	878.7002	0.49 ± 0.35	-	-
PC 44:6e; $[M+H]^+$	$C_{52}H_{94}NO_7P$	6.51	876.6816	876.6846	0.15 ± 0.11	-	-
PE 28:0; $[M+H]^+$	$C_{33}H_{66}NO_8P$	4.05	636.4584	636.4604	1.13 ± 1.48	1.02 ± 0.83	✓
PE 28:1e; PE 16:1e/12:0; $[M+H]^+$	$C_{33}H_{66}NO_7P$	4.48	620.4620	620.4655	0.84 ± 0.50	-	-
PE 29:0; $[M+H]^+$	$C_{34}H_{68}NO_8P$	4.41	650.4722	650.4761	1.04 ± 1.50	-	-
PE 30:0; $[M+H]^+$	$C_{35}H_{70}NO_8P$	4.66	664.4858	664.4917	12.7 ± 12.6		✓
PE 30:1; $[M+H]^+$	$C_{35}H_{68}NO_8P$	4.29	662.4731	662.4761	1.41 ± 1.56	-	-
PE 31:0; $[M+H]^+$	$C_{36}H_{72}NO_8P$	5.01	678.5065	678.5074	3.63 ± 3.44	3.37 ± 3.04	✓
PE 32:0; $[M+H]^+$	$C_{37}H_{74}NO_8P$	5.44	692.5203	692.5230	1.04 ± 0.92		-
PE 32:1; PE 16:0-16:1; $[M+H]^+$	$C_{37}H_{72}NO_8P$	4.89	690.5102	690.5074	11.1 ± 10.9	11.0 ± 10.0	✓
PE 32:2; PE 16:1-16:1; $[M-H]^-$	$C_{37}H_{70}NO_8P$	4.41	686.4703	686.4766	-	0.72 ± 0.89	-
PE 33:1; $[M+H]^+$	$C_{38}H_{74}NO_8P$	5.10	704.5194	704.5230	0.94 ± 0.92	-	-
PE 33:2; $[M+H]^+$	$C_{38}H_{72}NO_8P$	4.68	702.5068	702.5074	2.68 ± 2.40	-	-
PE 34:1; PE 16:0-18:1; $[M-H]^-$	$C_{39}H_{76}NO_8P$	5.54	716.5219	716.5236	-	5.68 ± 3.3	-

PE 34:2; [M+H]$^+$	$C_{39}H_{74}NO_8P$	5.01	716.5208	716.5230	4.03 ± 2.85	-	-
PE 34:2e; PE 16:1e/18:1; [M-H]$^-$	$C_{39}H_{76}NO_7P$	5.88	700.5268	700.5287	-	8.74 ± 3.43	-
PE 36:1; [M+H]$^+$	$C_{41}H_{80}NO_8P$	6.18	746.5713	746.5699	8.40 ± 4.62	8.00 ± 4.70	✓
PE 36:2; PE 18:1-18:1; [M-H]$^-$	$C_{41}H_{78}NO_8P$	5.63	742.5357	742.5392	-	1.87 ± 1.17	-
PE 36:3e; [M+H]$^+$	$C_{41}H_{78}NO_7P$	5.98	728.5593	728.5594	4.85 ± 2.84	-	-
PE 36:5e; PE 16:1e/20:4; [M-H]$^-$	$C_{41}H_{74}NO_7P$	5.24	722.5116	722.5130	-	7.22 ± 3.86	-
PE 38:4; [M+H]$^+$	$C_{43}H_{78}NO_8P$	5.56	768.5538	768.5543	1.14 ± 0.77	-	-
PE 38:5e; PE 18:1e/20:4; [M+H]$^+$	$C_{43}H_{78}NO_7P$	5.87	752.5632	752.5594	9.99 ± 5.43	10.41 ± 6.63	✓
PE 40:5e; PE 18:1e/22:4; [M+H]$^+$	$C_{45}H_{82}NO_7P$	6.35	780.5873	780.5907	1.13 ± 0.59	-	-
PG 32:1; [M+NH$_4$]$^+$	$C_{38}H_{73}O_{10}P$	4.20	738.5277	738.5285	2.76 ± 2.74	-	-
PG 34:1; PG 16:0-18:1; [M+NH$_4$]$^+$	$C_{40}H_{77}O_{10}P$	4.70	766.5609	766.5598	22.5 ± 16.4	-	-
PG 36:2; [M+NH$_4$]$^+$	$C_{42}H_{79}O_{10}P$	4.73	792.5718	792.5754	2.68 ± 2.06	-	-
PI 36:2; [M+NH$_4$]$^+$	$C_{45}H_{83}O_{13}P$	4.68	880.5850	880.5915	2.17 ± 1.25	-	-
PI 38:4; [M+NH$_4$]$^+$	$C_{47}H_{83}O_{13}P$	4.63	904.5952	904.5915	3.07 ± 2.38	-	-
SM d32:0; [M+H]$^+$	$C_{37}H_{77}N_2O_6P$	4.18	677.5546	677.5597	0.21 ± 0.06	-	-
SM d32:1; [M+H]$^+$	$C_{37}H_{75}N_2O_6P$	3.98	675.5446	675.5441	1.60 ± 0.74	-	-
SM d33:0; [M+H]$^+$	$C_{38}H_{79}N_2O_6P$	4.48	691.5831	691.5754	0.14 ± 0.05	-	-
SM d33:1; [M+H]$^+$	$C_{38}H_{77}N_2O_6P$	4.26	689.5631	689.5597	1.09 ± 0.45	-	-
SM d34:0; [M+H]$^+$	$C_{39}H_{81}N_2O_6P$	4.79	705.5962	705.5910	4.83 ± 1.57	-	-
SM d34:1; [M+H]$^+$	$C_{39}H_{79}N_2O_6P$	4.56	703.5783	703.5754	31.5 ± 13.8	93.01 ± 45.03	✓
SM d34:2; [M+H]$^+$	$C_{39}H_{77}N_2O_6P$	4.07	701.5584	701.5597	0.93 ± 0.47	-	-
SM d35:1; [M+H]$^+$	$C_{40}H_{81}N_2O_6P$	4.88	717.5947	717.5910	0.44 ± 0.18	-	-
SM d36:0; [M+H]$^+$	$C_{41}H_{85}N_2O_6P$	5.45	733.6315	733.6223	0.94 ± 0.29	-	-
SM d36:1; [M+H]$^+$	$C_{41}H_{83}N_2O_6P$	5.21	731.6081	731.6067	1.91 ± 0.48	-	-
SM d36:2; [M+H]$^+$	$C_{41}H_{81}N_2O_6P$	4.67	729.5952	729.5910	0.71 ± 0.31	-	-
SM d38:0; [M+H]$^+$	$C_{43}H_{89}N_2O_6P$	6.14	761.6523	761.6536	0.53 ± 0.13	-	-
SM d38:1; [M+H]$^+$	$C_{43}H_{87}N_2O_6P$	5.89	759.6450	759.6380	1.03 ± 0.23	-	-
SM d40:0; [M+H]$^+$	$C_{45}H_{93}N_2O_6P$	6.82	789.6868	789.6849	0.45 ± 0.11	-	-
SM d40:1; [M+H]$^+$	$C_{45}H_{91}N_2O_6P$	6.58	787.6707	787.6693	1.90 ± 0.50	-	-
SM d40:2; [M+H]$^+$	$C_{45}H_{89}N_2O_6P$	5.89	785.6514	785.6536	0.54 ± 0.30	-	-
SM d41:1; [M+H]$^+$	$C_{46}H_{93}N_2O_6P$	6.91	801.6835	801.6849	0.46 ± 0.16	-	-
SM d42:0; [M+H]$^+$	$C_{47}H_{97}N_2O_6P$	7.49	817.7238	817.7162	0.58 ± 0.19	-	-
SM d42:1; [M+H]$^+$	$C_{47}H_{95}N_2O_6P$	7.25	815.7084	815.7006	3.05 ± 0.82	-	-
SM d42:2; [M+H]$^+$	$C_{47}H_{93}N_2O_6P$	6.55	813.6857	813.6849	8.53 ± 3.65	21.11 ± 11.04	✓
SM d42:3; [M+H]$^+$	$C_{47}H_{91}N_2O_6P$	5.98	811.6752	811.6693	1.48 ± 0.68	-	-

SM d44:1; $[M+H]^+$	$C_{49}H_{99}N_2O_6P$	7.90	843.7291	843.7319	0.46 ± 0.19	-	-
SM d44:2; $[M+H]^+$	$C_{49}H_{97}N_2O_6P$	7.21	841.7139	841.7162	0.42 ± 0.13	-	-
SM d44:3; $[M+H]^+$	$C_{49}H_{95}N_2O_6P$	6.63	839.6998	839.7006	0.10 ± 0.06	-	-
TAG 24:0; TAG 8:0-8:0-8:0; $[M+NH_4]^+$	$C_{27}H_{50}O_6$	3.86	488.3964	488.3951	1.05 ± 0.78	-	-
TAG 24:0e; TAG 8:0e-8:0-8:0; $[M+NH_4]^+$	$C_{27}H_{52}O_5$	3.66	474.4125	474.4158	0.61 ± 0.86	-	-
TAG 26:0; TAG 8:0-8:0-10:0; $[M+NH_4]^+$	$C_{29}H_{54}O_6$	4.25	516.4261	516.4264	0.92 ± 0.75	-	-
TAG 28:0; TAG 8:0-10:0-10:0; $[M+NH_4]^+$	$C_{31}H_{58}O_6$	5.01	544.4542	544.4577	14.0 ± 13.6	-	-
TAG 38:0; TAG 10:0-14:0-14:0; $[M+NH_4]^+$	$C_{41}H_{78}O_6$	8.27	684.6138	684.6142	100.5 ± 95.5	-	-
TAG 39:0; TAG 10:0-14:0-15:0; $[M+NH_4]^+$	$C_{42}H_{80}O_6$	8.53	698.6290	698.6298	8.82 ± 8.42	-	-
TAG 40:0; TAG 10:0-14:0-16:0; $[M+NH_4]^+$	$C_{43}H_{82}O_6$	8.80	712.6452	712.6455	55.3 ± 52.6	-	-
TAG 40:1; TAG 8:0-14:0-18:1; $[M+NH_4]^+$	$C_{43}H_{80}O_6$	8.32	710.6303	710.6298	66.6 ± 64.1	-	-
TAG 41:0; TAG 10:0-15:0-16:0; $[M+NH_4]^+$	$C_{44}H_{84}O_6$	9.06	726.6583	726.6611	5.97 ± 5.42	-	-
TAG 41:1; TAG 8:0-15:0-18:1; $[M+NH_4]^+$	$C_{44}H_{82}O_6$	8.56	724.6418	724.6455	4.28 ± 4.18	-	-
TAG 42:0; TAG 12:0-14:0-16:0; $[M+NH_4]^+$	$C_{45}H_{86}O_6$	9.34	740.6755	740.6768	43.7 ± 41.9	-	-
TAG 42:1; TAG 8:0-16:0-18:1; $[M+NH_4]^+$	$C_{45}H_{84}O_6$	8.85	738.6611	738.6611	38.6 ± 37.1	-	-
TAG 42:2; TAG 8:0-16:1-18:1; $[M+NH_4]^+$	$C_{45}H_{82}O_6$	8.37	736.6420	736.6455	15.5 ± 15.2	-	-
TAG 42:3; TAG 8:0-16:0-18:3; $[M+NH_4]^+$	$C_{45}H_{80}O_6$	7.88	734.6263	734.6298	3.54 ± 3.58	-	-
TAG 43:0; TAG 12:0-15:0-16:0; $[M+NH_4]^+$	$C_{46}H_{88}O_6$	9.56	754.6940	754.6924	5.79 ± 4.60	-	-
TAG 43:1; TAG 10:0-15:0-18:1; $[M+NH_4]^+$	$C_{46}H_{86}O_6$	9.10	752.6772	752.6768	3.83 ± 3.67	-	-
TAG 44:0; TAG 12:0-14:0-18:0; $[M+NH_4]^+$	$C_{47}H_{90}O_6$	9.84	768.7082	768.7081	35.1 ± 32.5	-	-
TAG 44:1; TAG 10:0-14:0-20:1; $[M+NH_4]^+$	$C_{47}H_{88}O_6$	9.36	766.6953	766.6924	36.9 ± 35.5	-	-
TAG 44:2; TAG 10:0-16:0-18:2; $[M+NH_4]^+$	$C_{47}H_{86}O_6$	8.91	764.6737	764.6768	12.2 ± 11.5	-	-
TAG 44:2; TAG 8:0-16:0-20:2; $[M+NH_4]^+$	$C_{47}H_{86}O_6$	8.37	764.6727	764.6768	5.69 ± 5.52	-	-
TAG 44:3; TAG 10:0-16:0-18:3; $[M+NH_4]^+$	$C_{47}H_{84}O_6$	8.45	762.6616	762.6611	2.56 ± 2.35	-	-
TAG 45:0; TAG 14:0-15:0-16:0; $[M+NH_4]^+$	$C_{48}H_{92}O_6$	10.04	782.7236	782.7237	7.67 ± 4.68	-	-
TAG 45:1; TAG 11:0-16:0-18:1; $[M+NH_4]^+$	$C_{48}H_{90}O_6$	9.62	780.7122	780.7081	4.43 ± 3.72	-	-
TAG 46:0; TAG 14:0-16:0-16:0; $[M+NH_4]^+$	$C_{49}H_{94}O_6$	10.31	796.7393	796.7394	33.9 ± 27.3	-	-
TAG 46:1; TAG 12:0-16:0-18:1; $[M+NH_4]^+$	$C_{49}H_{92}O_6$	9.87	794.7217	794.7237	40.6 ± 37.9	-	-
TAG 46:2; TAG 10:0-18:1-18:1; $[M+NH_4]^+$	$C_{49}H_{90}O_6$	9.41	792.7098	792.7081	14.6 ± 13.3	-	-
TAG 46:3; TAG 10:0-18:1-18:2; $[M+NH_4]^+$	$C_{49}H_{88}O_6$	8.99	790.6907	790.6924	3.62 ± 3.34	-	-
TAG 46:4; TAG 10:0-18:1-18:3; $[M+NH_4]^+$	$C_{49}H_{86}O_6$	8.51	788.6785	788.6768	0.54 ± 0.63	-	-
TAG 47:0; TAG 14:0-16:0-17:0; $[M+NH_4]^+$	$C_{50}H_{96}O_6$	10.49	810.7529	810.7550	7.99 ± 4.49	-	-
TAG 47:1; TAG 14:0-15:0-18:1; $[M+NH_4]^+$	$C_{50}H_{94}O_6$	10.08	808.7389	808.7394	6.62 ± 5.18	-	-
TAG 48:0; TAG 14:0-16:0-18:0; $[M+NH_4]^+$	$C_{51}H_{98}O_6$	10.74	824.7735	824.7707	38.7 ± 24.5	-	-
TAG 48:1; TAG 14:0-16:0-18:1; $[M+NH_4]^+$	$C_{51}H_{96}O_6$	10.32	822.7526	822.7550	65.0 ± 55.9	-	-

TAG 48:2; TAG 14:0-16:1-18:1; $[M+NH_4]^+$	$C_{51}H_{94}O_6$	9.90	820.7402	820.7394	23.0 ± 18.7	-	-
TAG 48:3; TAG 14:1-16:1-18:1; $[M+NH_4]^+$	$C_{51}H_{92}O_6$	9.49	818.7248	818.7237	4.73 ± 3.65	-	-
TAG 48:4; TAG 16:0-14:1-18:3; $[M+NH_4]^+$	$C_{51}H_{90}O_6$	9.05	816.7084	816.7081	0.64 ± 0.58	-	-
TAG 49:1; TAG 15:0-16:0-18:1; $[M+NH_4]^+$	$C_{52}H_{98}O_6$	10.51	836.7684	836.7707	9.91 ± 7.97	-	-
TAG 49:2; TAG 18:0-15:1-16:1; $[M+NH_4]^+$	$C_{52}H_{96}O_6$	10.11	834.7556	834.7550	16.8 ± 4.3	-	-
TAG 50:0; TAG 16:0-16:0-18:0; $[M+NH_4]^+$	$C_{53}H_{102}O_6$	11.139	852.7974	852.8020	17.6 ± 11.6	-	-
TAG 50:1; TAG 16:0-18:0-16:1; $[M+NH_4]^+$	$C_{53}H_{100}O_6$	10.742	850.7852	850.7863	177 ± 153.3	-	-
TAG 50:2; TAG 16:0-16:1-18:1; $[M+NH_4]^+$	$C_{53}H_{98}O_6$	10.36	848.7692	848.7707	76.8 ± 62.2	-	-
TAG 50:3; TAG 16:0-16:1-18:2; $[M+NH_4]^+$	$C_{53}H_{96}O_6$	9.96	846.7558	846.7550	15.1 ± 9.6	-	-
TAG 50:4; TAG 14:0-18:1-18:3; $[M+NH_4]^+$	$C_{53}H_{94}O_6$	9.55	844.7330	844.7394	2.75 ± 1.81	-	-
TAG 51:0; TAG 16:0-17:0-18:0; $[M+NH_4]^+$	$C_{54}H_{104}O_6$	11.29	866.8231	866.8176	1.76 ± 1.14	-	-
TAG 51:2; TAG 16:0-17:0-18:2; $[M+NH_4]^+$	$C_{54}H_{100}O_6$	10.54	862.7831	862.7863	7.03 ± 4.37	-	-
TAG 51:3; TAG 15:1-18:1-18:1; $[M+NH_4]^+$	$C_{54}H_{98}O_6$	10.17	860.7748	860.7707	2.60 ± 1.31	-	-
TAG 51:4; TAG 15:0-18:2-18:2; $[M+NH_4]^+$	$C_{54}H_{96}O_6$	9.79	858.7528	858.7550	0.63 ± 0.52	-	-
TAG 52:0; TAG 16:0-18:0-18:0; $[M+NH_4]^+$	$C_{55}H_{106}O_6$	11.51	880.8361	880.8333	7.05 ± 5.25	-	-
TAG 52:1; TAG 16:0-18:0-18:1; $[M+NH_4]^+$	$C_{55}H_{104}O_6$	11.14	878.8201	878.8176	177.2 ± 246.4	-	-
TAG 52:2; TAG 16:0-18:0-18:2; $[M+NH_4]^+$	$C_{55}H_{102}O_6$	10.77	876.8047	876.8020	270.9 ± 274.2	-	-
TAG 52:3; TAG 16:0-18:1-18:2; $[M+NH_4]^+$	$C_{55}H_{100}O_6$	10.39	874.7915	874.7863	208.4 ± 280.4	-	-
TAG 52:4; TAG 16:0-18:2-18:2; $[M+NH_4]^+$	$C_{55}H_{98}O_6$	10.01	872.7692	872.7707	107 ± 163.3	-	-
TAG 52:5; TAG 16:0-18:2-18:3; $[M+NH_4]^+$	$C_{55}H_{96}O_6$	9.62	870.7573	870.7550	8.18 ± 10.20	-	-
TAG 53:0; TAG 16:0-16:0-21:0; $[M+NH_4]^+$	$C_{56}H_{108}O_6$	11.67	894.8473	894.8489	0.89 ± 0.41	-	-
TAG 53:1; TAG 16:0-18:0-19:1; $[M+NH_4]^+$	$C_{56}H_{106}O_6$	11.31	892.8369	892.8333	2.89 ± 1.99	-	-
TAG 53:2; TAG 18:0-17:1-18:1; $[M+NH_4]^+$	$C_{56}H_{104}O_6$	10.95	890.8143	890.8176	4.59 ± 2.67	-	-
TAG 53:3; TAG 16:0-19:1-18:2; $[M+NH_4]^+$	$C_{56}H_{102}O_6$	10.58	888.8076	888.8020	3.64 ± 2.63	-	-
TAG 53:4; TAG 17:0-18:2-18:2; $[M+NH_4]^+$	$C_{56}H_{100}O_6$	10.22	886.7903	886.7863	1.65 ± 1.41	-	-
TAG 54:0; TAG 14:0-18:0-22:0; $[M+NH_4]^+$	$C_{57}H_{110}O_6$	11.78	908.8678	908.8646	4.43 ± 4.33	-	-
TAG 54:1; TAG 18:0-18:0-18:1; $[M+NH_4]^+$	$C_{57}H_{108}O_6$	11.51	906.8496	906.8489	86.5 ± 144.3	-	-
TAG 54:2; TAG 18:0-18:1-18:1; $[M+NH_4]^+$	$C_{57}H_{106}O_6$	11.16	904.8344	904.8333	93.0 ± 127.5	-	-
TAG 54:3; TAG 18:0-18:1-18:2; $[M+NH_4]^+$	$C_{57}H_{104}O_6$	10.78	902.8228	902.8176	497.4 ± 722.1	-	-
TAG 54:4; TAG 18:1-18:1-18:2; $[M+NH_4]^+$	$C_{57}H_{102}O_6$	10.41	900.8036	900.8020	399.8 ± 656.4	-	-
TAG 54:5; TAG 18:1-18:2-18:2; $[M+NH_4]^+$	$C_{57}H_{100}O_6$	10.03	898.7903	898.7863	297.1 ± 512.4	-	-
TAG 54:6; TAG 18:1-18:2-18:3; $[M+NH_4]^+$	$C_{57}H_{98}O_6$	9.63	896.7699	896.7707	126.3 ± 233.7	-	-
TAG 54:7; TAG 18:2-18:2-18:3; $[M+NH_4]^+$	$C_{57}H_{96}O_6$	9.26	894.7510	894.7550	7.85 ± 14.05	-	-
TAG 55:0; TAG 15:0-16:0-24:0; $[M+NH_4]^+$	$C_{58}H_{112}O_6$	11.86	922.8812	922.8802	1.21 ± 0.36	-	-
TAG 55:1; TAG 16:0-21:0-18:1; $[M+NH_4]^+$	$C_{58}H_{110}O_6$	11.66	920.8672	920.8646	0.68 ± 0.49	-	-

TAG 56:0; TAG 14:0-16:0-26:0; $[M+NH_4]^+$	$C_{59}H_{114}O_6$	11.92	936.9025	936.8959	1.95 ± 0.69	-	-
TAG 56:1; TAG 18:0-20:0-18:1; $[M+NH_4]^+$	$C_{59}H_{112}O_6$	11.78	934.8783	934.8802	7.32 ± 12.24	-	-
TAG 56:2; TAG 20:0-18:1-18:1; $[M+NH_4]^+$	$C_{59}H_{110}O_6$	11.53	932.8666	932.8646	9.53 ± 17.01	-	-
TAG 57:0; TAG 15:0-16:0-26:0; $[M+NH_4]^+$	$C_{60}H_{116}O_6$	11.98	950.9077	950.9115	1.18 ± 0.32	-	-
TAG 57:1; TAG 16:0-23:0-18:1; $[M+NH_4]^+$	$C_{60}H_{114}O_6$	11.86	948.8961	948.8959	0.56 ± 0.39	-	-
TAG 58:0; TAG 16:0-17:0-25:0; $[M+NH_4]^+$	$C_{61}H_{118}O_6$	12.01	964.9335	964.9272	1.73 ± 0.50	-	-
TAG 58:1; TAG 16:0-24:0-18:1; $[M+NH_4]^+$	$C_{61}H_{116}O_6$	11.92	962.9102	962.9115	2.86 ± 4.04	-	-
TAG 58:2; TAG 16:0-20:1-22:1; $[M+NH_4]^+$	$C_{61}H_{114}O_6$	11.79	960.8931	960.8959	6.41 ± 10.05	-	-
TAG 58:3; TAG 22:0-18:1-18:2; $[M+NH_4]^+$	$C_{61}H_{112}O_6$	11.54	958.8799	958.8802	5.09 ± 10.83	-	-
TAG 58:4; TAG 22:0-18:2-18:2; $[M+NH_4]^+$	$C_{61}H_{110}O_6$	11.26	956.8632	956.8646	3.07 ± 6.53	-	-
TAG 59:0; TAG 16:0-17:0-26:0; $[M+NH_4]^+$	$C_{62}H_{120}O_6$	12.03	978.9441	978.9428	0.79 ± 0.29	-	-
TAG 59:2; TAG 19:0-19:0-21:2; $[M+NH_4]^+$	$C_{62}H_{116}O_6$	11.86	974.9102	974.9115	0.54 ± 0.43	-	-
TAG 60:1; TAG 16:0-26:0-18:1; $[M+NH_4]^+$	$C_{63}H_{120}O_6$	12.01	990.9482	990.9428	1.28 ± 1.43	-	-
TAG 60:2; TAG 24:0-18:1-18:1; $[M+NH_4]^+$	$C_{63}H_{118}O_6$	11.92	988.9281	988.9272	2.66 ± 3.20	-	-
TAG 60:3; TAG 24:0-18:1-18:2; $[M+NH_4]^+$	$C_{63}H_{116}O_6$	11.79	986.9155	986.9115	2.70 ± 4.35	-	-

The method does not chromatographically resolve lipid species with identical total number of side chain carbons. The primary species that was identified by MS-DIAL is given if sufficient MS/MS information was present to reach the set identification threshold. Concentration values are given in ng per mg of dried sample. Although PE species were detected in negative mode, no quantification was conducted due to insufficient sensitivity of the corresponding surrogate calibrant in negative mode. 60.6% of all features that could be identified in negative mode were also detected in positive mode

Table S6 Identified lipids and estimated concentration levels in cultivated *in vitro* biofilm samples

**Identified lipid; primary species; [adduct type]**	**Sum formula**	**t_R [min]**	**Measured precursor *m/z***	**Calculated precursor *m/z***	**Mean calculated concentration [ng*mg^{-1}] pos. mode**	**Mean calculated concentration [ng*mg^{-1}] neg. mode**	**ID in both polarity modes**
CE 18:2; $[M+NH_4]^+$	$C_{45}H_{76}O_2$	10.32	666.6193	666.6189	35.5 ± 44.6	-	-
CE 20:4; $[M+NH_4]^+$	$C_{47}H_{76}O_2$	10.02	690.6177	690.6189	33.4 ± 61.1	-	-
Cer-BDS d33:0+O; Cer-BDS d16:0/17:0+O; $[M+HCOO]^-$	$C_{33}H_{67}NO_4$	4.62	586.5012	586.5052	-	-	-
Cer-BDS d34:0+O; Cer-BDS d17:0/17:0+O; $[M+HCOO]^-$	$C_{34}H_{69}NO_4$	4.89	600.5175	600.5209	-	-	-
Cer-BDS d35:0+O; Cer-BDS d18:0/17:0+O; $[M+HCOO]^-$	$C_{35}H_{71}NO_4$	5.25	614.5360	614.5365	-	-	-
Cer-BDS d36:0+O; Cer-BDS d19:0/17:0+O; $[M+HCOO]^-$	$C_{36}H_{73}NO_4$	5.50	628.5465	628.5522	-	-	-
Cer-NDS d20:0; Cer-NDS d18:0/2:0; $[M+H]^+$	$C_{20}H_{41}NO_3$	1.98	344.3154	344.3165	-	-	-
Cer-NDS d34:0; Cer-NDS d18:0/16:0; $[M+H]^+$	$C_{34}H_{69}NO_3$	5.68	540.5331	540.5355	-	-	-
Cer-NDS d38:0; Cer-NDS d20:0/18:0; $[M+H]^+$	$C_{38}H_{77}NO_3$	7.08	596.5954	596.5981	-	-	-
Cer-NDS d40:0; Cer-NDS d18:0/22:0; $[M+H]^+$	$C_{40}H_{81}NO_3$	7.76	624.6298	624.6294	-	-	-
Cer-NDS d41:0; Cer-NDS d18:0/23:0; $[M+H]^+$	$C_{41}H_{83}NO_3$	8.08	638.6414	638.6451	-	-	-
Cer-NDS d42:0; Cer-NDS d18:0/24:0; $[M+H]^+$	$C_{42}H_{85}NO_3$	8.39	652.6617	652.6607	-	-	-
Cer-NDS d44:0; Cer-NDS d18:0/26:0; $[M+H]^+$	$C_{44}H_{89}NO_3$	8.99	680.6941	680.6920	-	-	-
Cer-NDS d44:1; Cer-NDS d18:0/26:1; $[M+H]^+$	$C_{44}H_{87}NO_3$	8.34	678.6771	678.6764	-	-	-
Cer-NDS d46:0; Cer-NDS d20:0/26:0; $[M+H]^+$	$C_{46}H_{93}NO_3$	9.51	708.7200	708.7233	-	-	-
Cer-NDS d46:1; Cer-NDS d20:0/26:1; $[M+H]^+$	$C_{46}H_{91}NO_3$	8.93	706.7001	706.7077	-	-	-
Cer-NS d33:1; Cer-NS d17:1/16:0; $[M+H]^+$	$C_{33}H_{65}NO_3$	5.11	524.5064	524.5042	-	-	-
Cer-NS d34:1; Cer-NS d18:1/16:0; $[M+H]^+$	$C_{34}H_{67}NO_3$	5.45	538.5209	538.5199	-	-	-
Cer-NS d34:2; Cer-NS d18:1/16:1; $[M+H]^+$	$C_{34}H_{65}NO_3$	5.15	536.5040	536.5042	-	-	-
Cer-NS d34:4; Cer-NS d18:1/16:3; $[M+H]^+$	$C_{34}H_{61}NO_3$	4.78	532.4699	532.4729	-	-	-
Cer-NS d35:4; Cer-NS d17:1/18:3; $[M+H]^+$	$C_{35}H_{63}NO_3$	5.10	546.4841	546.4886	-	-	-
Cer-NS d36:4; Cer-NS d18:1/18:3; $[M+H]^+$	$C_{36}H_{65}NO_3$	5.46	560.5035	560.5042	-	-	-
Cer-NS d40:1; Cer-NS d18:1/22:0; $[M+H]^+$	$C_{40}H_{79}NO_3$	7.55	622.6108	622.6138	-	-	-
Cer-NS d41:1; Cer-NS d16:1/25:0; $[M+H]^+$	$C_{41}H_{81}NO_3$	7.86	636.6232	636.6294	-	-	-
Cer-NS d42:1; Cer-NS d18:1/24:0; $[M+H]^+$	$C_{42}H_{83}NO_3$	8.17	650.6423	650.6451	-	-	-
Cer-NS d42:4; Cer-NS d18:1/24:3; $[M+H]^+$	$C_{42}H_{77}NO_3$	7.53	644.5900	644.5981	-	-	-
Cer-NS d43:1; Cer-NS d18:1/25:0; $[M+H]^+$	$C_{43}H_{85}NO_3$	8.48	664.6579	664.6607	-	-	-
Cer-NS d44:1; Cer-NS d18:1/26:0; $[M+H]^+$	$C_{44}H_{87}NO_3$	8.78	678.6757	678.6764	-	-	-

Cer-NS d44:4; Cer-NS d18:1/26:3; [M+H]$^+$	$C_{44}H_{81}NO_3$	8.17	672.6234	672.6294	-	-	-
Cer-NS d45:1; Cer-NS d20:1/25:0; [M+H]$^+$	$C_{45}H_{89}NO_3$	9.05	692.6926	692.6920	-	-	-
Cer-NS d46:1; Cer-NS d18:1/28:0; [M+H]$^+$	$C_{46}H_{91}NO_3$	9.33	706.7056	706.7077	-	-	-
Cholesterol; [M-H_2O+H]$^+$	$C_{27}H_{46}O$	4.66	369.3512	369.3516	314.0 ± 242.1	-	-
Cholesterol-sulfate; [M-H]$^-$	$C_{27}H_{46}O_4S$	2.95	465.3035	465.3044	-	-	-
DAG 20:0; DAG 8:0-12:0; [M+NH_4]$^+$	$C_{23}H_{44}O_5$	3.17	418.3568	418.3532	1.01 ± 0.59	-	-
DAG 24:0; DAG 10:0-14:0; [M+NH_4]$^+$	$C_{27}H_{52}O_5$	4.11	474.4153	474.4158	1.42 ± 1.74	-	-
DAG 25:0; DAG 12:0-13:0; [M+NH_4]$^+$	$C_{28}H_{54}O_5$	4.39	488.4340	488.4315	0.65 ± 0.26	-	-
DAG 26:0; DAG 12:0-14:0; [M+NH_4]$^+$	$C_{29}H_{56}O_5$	4.68	502.4481	502.4471	13.25 ± 5.65	-	-
DAG 26:1e; DAG 13:1e/13:0; [M+NH_4]$^+$	$C_{29}H_{56}O_4$	5.20	486.4505	486.4522	0.72 ± 0.28	-	-
DAG 27:0; DAG 12:0-15:0; [M+NH_4]$^+$	$C_{30}H_{58}O_5$	4.99	516.4616	516.4628	6.97 ± 2.63	-	-
DAG 27:1e; DAG 15:1e/12:0; [M+NH_4]$^+$	$C_{30}H_{58}O_4$	5.54	500.4648	500.4679	0.76 ± 0.32	-	-
DAG 28:0; DAG 13:0-15:0; [M+NH_4]$^+$	$C_{31}H_{60}O_5$	5.32	530.4797	530.4784	38.1 ± 19.0	-	-
DAG 28:1; DAG 12:0-16:1; [M+NH_4]$^+$	$C_{31}H_{58}O_5$	4.78	528.4675	528.4628	5.75 ± 2.67	-	-
DAG 28:1e; DAG 15:1e/13:0; [M+NH_4]$^+$	$C_{31}H_{60}O_4$	5.88	514.4822	514.4835	7.65 ± 3.53	-	-
DAG 28:2e; DAG 17:2e/11:0; [M+NH_4]$^+$	$C_{31}H_{58}O_4$	5.30	512.4689	512.4679	2.09 ± 0.65	-	-
DAG 29:0; DAG 13:0-16:0; [M+NH_4]$^+$	$C_{32}H_{62}O_5$	5.66	544.4944	544.4941	7.02 ± 1.52	-	-
DAG 29:0e; DAG 15:0e/14:0; [M+NH_4]$^+$	$C_{32}H_{64}O_4$	6.56	530.5237	530.5148	0.3 ± 0.12	-	-
DAG 29:1; DAG 13:0-16:1; [M+NH_4]$^+$	$C_{32}H_{60}O_5$	5.11	542.4778	542.4784	6.43 ± 2.2	-	-
DAG 29:1e; DAG 16:1e/13:0; [M+NH_4]$^+$	$C_{32}H_{62}O_4$	6.24	528.4965	528.4992	1.53 ± 0.93	-	-
DAG 30:0; DAG 14:0-16:0; [M+NH_4]$^+$	$C_{33}H_{64}O_5$	6.00	558.5118	558.5097	44.4 ± 28.7	-	-
DAG 30:1; DAG 14:0-16:1; [M+NH_4]$^+$	$C_{33}H_{62}O_5$	5.42	556.4970	556.4941	36.5 ± 20.3	-	-
DAG 30:2e; DAG 13:1e/17:1; [M+NH_4]$^+$	$C_{33}H_{62}O_4$	5.98	540.4990	540.4992	14.9 ± 5.1	-	-
DAG 31:0; DAG 15:0-16:0; [M+NH_4]$^+$	$C_{34}H_{66}O_5$	6.24	572.5247	572.5254	8.66 ± 7.47	-	-
DAG 31:1; DAG 15:0-16:1; [M+NH_4]$^+$	$C_{34}H_{64}O_5$	5.75	570.5121	570.5097	24.6 ± 7.1	-	-
DAG 31:2; DAG 15:1-16:1; [M+NH_4]$^+$	$C_{34}H_{62}O_5$	5.27	568.4959	568.4941	6.45 ± 2.68	-	-
DAG 31:2e; DAG 16:2e/15:0; [M+NH_4]$^+$	$C_{34}H_{64}O_4$	6.33	554.5126	554.5148	9.88 ± 3.57	-	-
DAG 32:0; DAG 16:0-16:0; [M+NH_4]$^+$	$C_{35}H_{68}O_5$	6.70	586.5446	586.5410	50.3 ± 28.4	-	-
DAG 32:1; DAG 16:0-16:1; [M+NH_4]$^+$	$C_{35}H_{66}O_5$	6.08	584.5276	584.5254	138.8 ± 103.6	-	-
DAG 32:2; DAG 16:1-16:1; [M+NH_4]$^+$	$C_{35}H_{64}O_5$	5.54	582.5140	582.5097	42.0 ± 19.4	-	-
DAG 32:2e; DAG 16:2e/16:0; [M+NH_4]$^+$	$C_{35}H_{66}O_4$	6.67	568.5280	568.5305	63.6 ± 24.6	-	-
DAG 32:3e; DAG 16:2e/16:1; [M+NH_4]$^+$	$C_{35}H_{64}O_4$	6.09	566.5123	566.5148	26.2 ± 7.8	-	-
DAG 33:0; DAG 16:0-17:0; [M+NH_4]$^+$	$C_{36}H_{70}O_5$	7.04	600.5581	600.5567	2.4 ± 0.77	-	-
DAG 33:1; DAG 15:0-18:1; [M+NH_4]$^+$	$C_{36}H_{68}O_5$	6.41	598.5387	598.5410	42.6 ± 16.4	-	-
DAG 33:2; DAG 16:1-17:1; [M+NH_4]$^+$	$C_{36}H_{66}O_5$	5.85	596.5227	596.5254	42.8 ± 15.4	-	-

DAG 33:3e; DAG 16:2e/17:1; [M+NH4]+	$C_{36}H_{66}O_4$	6.41	580.5305	580.5305	26.3 ± 8.2	-	-
DAG 34:0; DAG 16:0-18:0; [M+NH4]+	$C_{37}H_{72}O_5$	7.37	614.5714	614.5723	32.2 ± 22.9	-	-
DAG 34:1; DAG 16:0-18:1; [M+NH4]+	$C_{37}H_{70}O_5$	6.74	612.5538	612.5567	307.2 ± 188.3	-	-
DAG 34:2; DAG 17:1-17:1; [M+NH4]+	$C_{37}H_{68}O_5$	6.18	610.5465	610.5410	119.7 ± 78.82	-	-
DAG 34:3; DAG 16:1-18:2; [M+NH4]+	$C_{37}H_{66}O_5$	5.74	608.5302	608.5254	4.29 ± 6.74	-	-
DAG 34:3e; DAG 18:3e/16:0; [M+NH4]+	$C_{37}H_{68}O_4$	6.73	594.5430	594.5461	91.0 ± 38.4	-	-
DAG 35:0; DAG 17:0-18:0; [M+NH4]+	$C_{38}H_{74}O_5$	7.69	628.5825	628.5880	1.25 ± 0.88	-	-
DAG 35:1; DAG 17:0-18:1; [M+NH4]+	$C_{38}H_{72}O_5$	7.08	626.5710	626.5723	13.9 ± 6.9	-	-
DAG 35:2; DAG 17:1-18:1; [M+NH4]+	$C_{38}H_{70}O_5$	6.48	624.5538	624.5567	19.9 ± 11.1	-	-
DAG 36:0; DAG 18:0-18:0; [M+NH4]+	$C_{39}H_{76}O_5$	8.02	642.6085	642.6036	53.7 ± 142.4	-	-
DAG 36:1; DAG 16:0-20:1; [M+NH4]+	$C_{39}H_{74}O_5$	7.40	640.5872	640.5880	160.8 ± 148.2	-	-
DAG 36:2; DAG 18:1-18:1; [M+NH4]+	$C_{39}H_{72}O_5$	6.80	638.5745	638.5723	127.3 ± 127.1	-	-
DAG 36:3; DAG 18:1-18:2; [M+NH4]+	$C_{39}H_{70}O_5$	6.28	636.5588	636.5567	36.7 ± 78.3	-	-
DAG 36:3e; DAG 20:3e/16:0; [M+NH4]+	$C_{39}H_{72}O_4$	7.38	622.5732	622.5774	11.6 ± 4.7	-	-
DAG 36:4; DAG 18:1-18:3; [M+NH4]+	$C_{39}H_{68}O_5$	5.76	634.5427	634.5410	27.1 ± 72.9	-	-
DAG 37:1; DAG 17:0-20:1; [M+NH4]+	$C_{40}H_{76}O_5$	7.71	654.6033	654.6036	3.53 ± 2.32	-	-
DAG 37:2; DAG 18:1-19:1; [M+NH4]+	$C_{40}H_{74}O_5$	7.11	652.5902	652.5880	1.78 ± 0.64	-	-
DAG 38:0; DAG 18:0-20:0; [M+NH4]+	$C_{41}H_{80}O_5$	8.63	670.6388	670.6349	3.66 ± 7.15	-	-
DAG 38:1; DAG 18:0-20:1; [M+NH4]+	$C_{41}H_{78}O_5$	8.04	668.6241	668.6193	40.6 ± 36.3	-	-
DAG 38:2; DAG 18:1-20:1; [M+NH4]+	$C_{41}H_{76}O_5$	7.43	666.5981	666.6036	19.9 ± 12.1	-	-
DAG 40:1; DAG 20:0-20:1; [M+NH4]+	$C_{43}H_{82}O_5$	8.64	696.6480	696.6506	4.45 ± 3.14	-	-
DAG 40:2; DAG 20:1-20:1; [M+NH4]+	$C_{43}H_{80}O_5$	8.04	694.6325	694.6349	5.26 ± 3.76	-	-
DAG 40:3; DAG 20:1-20:2; [M+NH4]+	$C_{43}H_{78}O_5$	7.52	692.6221	692.6193	0.33 ± 0.19	-	-
DGDG 26:0; DGDG 12:0-14:0; [M+NH4]+	$C_{41}H_{76}O_{15}$	3.61	826.5479	826.5528	-	-	-
DGDG 28:0; DGDG 12:0-16:0; [M+NH4]+	$C_{43}H_{80}O_{15}$	4.11	854.5868	854.5841	-	-	✓
DGDG 29:0; DGDG 14:0-15:0; [M+NH4]+	$C_{44}H_{82}O_{15}$	4.38	868.6011	868.5997	-	-	✓
DGDG 30:0; DGDG 14:0-16:0; [M+NH4]+	$C_{45}H_{84}O_{15}$	4.68	882.6173	882.6154	-	-	✓
DGDG 30:1; DGDG 16:0-14:1; [M+NH4]+	$C_{45}H_{82}O_{15}$	4.21	880.5978	880.5997	-	-	✓
DGDG 31:0; DGDG 15:0-16:0; [M+NH4]+	$C_{46}H_{86}O_{15}$	4.98	896.6327	896.6310	-	-	✓
DGDG 31:1; DGDG 15:0-16:1; [M+NH4]+	$C_{46}H_{84}O_{15}$	4.48	894.6148	894.6154	-	-	-
DGDG 32:0; DGDG 16:0-16:0; [M+NH4]+	$C_{47}H_{88}O_{15}$	5.30	910.6454	910.6467	-	-	✓
DGDG 32:1; DGDG 16:0-16:1; [M+NH4]+	$C_{47}H_{86}O_{15}$	4.74	908.6252	908.6310	-	-	✓
DGDG 32:2; DGDG 14:0-18:2; [M+NH4]+	$C_{47}H_{84}O_{15}$	4.31	906.6155	906.6154	-	-	-
DGDG 33:0; DGDG 16:0-17:0; [M+NH4]+	$C_{48}H_{90}O_{15}$	5.62	924.6664	924.6623	-	-	-
DGDG 33:1; DGDG 16:0-17:1; [M+NH4]+	$C_{48}H_{88}O_{15}$	5.04	922.6489	922.6467	-	-	-

DGDG 33:2; [M+HCOO]$^{-}$	$C_{48}H_{86}O_{15}$	4.54	947.6114	947.5949	-	-	-
DGDG 34:0; DGDG 16:0-18:0; [M+NH$_4$]$^{+}$	$C_{49}H_{92}O_{15}$	5.95	938.6730	938.6780	-	-	✓
DGDG 34:1; DGDG 16:0-18:1; [M+NH$_4$]$^{+}$	$C_{49}H_{90}O_{15}$	5.35	936.6611	936.6623	-	-	✓
DGDG 34:2; DGDG 16:0-18:2; [M+NH$_4$]$^{+}$	$C_{49}H_{88}O_{15}$	4.87	934.6502	934.6467	-	-	✓
DGDG 35:1; DGDG 17:0-18:1; [M+NH$_4$]$^{+}$	$C_{50}H_{92}O_{15}$	5.67	950.6761	950.6780	-	-	-
DGDG 36:0; [M+HCOO]$^{-}$	$C_{51}H_{96}O_{15}$	6.62	993.6779	993.6731	-	-	-
DGDG 36:1; DGDG 16:0-20:1; [M+NH$_4$]$^{+}$	$C_{51}H_{94}O_{15}$	5.99	964.7014	964.6936	-	-	✓
DGDG 36:2; DGDG 18:1-18:1; [M+HCOO]$^{-}$	$C_{51}H_{92}O_{15}$	5.44	989.6401	989.6418	-	-	-
DGDG 38:1; DGDG 18:0-20:1; [M+HCOO]$^{-}$	$C_{53}H_{98}O_{15}$	6.65	1019.6896	1019.6888	-	-	-
DGDG 38:2; [M+HCOO]$^{-}$	$C_{53}H_{96}O_{15}$	6.05	1017.6682	1017.6731	-	-	-
FA 20:0; [M-H]$^{-}$	$C_{20}H_{40}O_2$	4.18	311.2922	311.2956	-	-	-
FA 20:1; [M-H]$^{-}$	$C_{20}H_{38}O_2$	3.63	309.2770	309.2799	-	-	-
FA 22:0; [M-H]$^{-}$	$C_{22}H_{44}O_2$	4.87	339.3256	339.3269	-	-	-
FA 22:1; [M-H]$^{-}$	$C_{22}H_{42}O_2$	4.21	337.3081	337.3112	-	-	-
FA 24:0; [M-H]$^{-}$	$C_{24}H_{48}O_2$	5.61	367.3553	367.3582	-	-	-
FA 24:1; [M-H]$^{-}$	$C_{24}H_{46}O_2$	4.87	365.3359	365.3425	-	-	-
FA 26:0; [M-H]$^{-}$	$C_{26}H_{52}O_2$	6.38	395.3857	395.3895	-	-	-
HBMP 52:1; HBMP 16:0-16:0-20:1; [M-H]$^{-}$	$C_{58}H_{111}O_{11}P$	9.47	1013.7886	1013.7791	-	-	-
HBMP 52:2; HBMP 16:0-18:1-18:1; [M-H]$^{-}$	$C_{58}H_{109}O_{11}P$	9.02	1011.7692	1011.7635	-	-	-
HBMP 48:1; HBMP 16:0/16:0-16:1; [M+NH$_4$]$^{+}$	$C_{54}H_{103}O_{11}P$	7.60	976.7543	976.7581	-	-	-
HexCer-NDS d34:0; [M+HCOO]$^{-}$	$C_{40}H_{79}NO_8$	5.07	746.5775	746.5788	-	-	-
HexCer-NDS d40:0; [M+H]$^{+}$	$C_{46}H_{91}NO_8$	7.08	786.6792	786.6823	-	-	-
HexCer-NDS d41:0; [M+HCOO]$^{-}$	$C_{47}H_{93}NO_8$	7.38	844.6857	844.6883	-	-	
HexCer-NDS d42:0; HexCer-NDS d18:0/24:0; [M+H]$^{+}$	$C_{48}H_{95}NO_8$	7.72	814.7120	814.7136	-	-	✓
HexCer-NDS d44:0; [M+H]$^{+}$	$C_{50}H_{99}NO_8$	8.34	842.7402	842.7449	-	-	-
HexCer-NDS d46:0; HexCer-NDS d20:0/26:0; [M+H]$^{+}$	$C_{52}H_{103}NO_8$	8.91	870.7714	870.7762	-	-	-
HexCer-NS d34:1; HexCer-NS d18:1/16:0; [M+H]$^{+}$	$C_{40}H_{77}NO_8$	4.83	700.5728	700.5727	-	-	✓
HexCer-NS d40:1; HexCer-NS d18:1/22:0; [M+H]$^{+}$	$C_{46}H_{89}NO_8$	6.85	784.6682	784.6666	-	-	-
HexCer-NS d42:2; HexCer-NS d18:1/24:1; [M+H]$^{+}$	$C_{48}H_{91}NO_8$	6.82	810.6832	810.6823	-	-	✓
HexHexCer d34:1; HexHexCer d18:1/16:0; [M+H]$^{+}$	$C_{46}H_{87}NO_{13}$	4.61	862.6236	862.6255	-	-	-
HexHexCer d42:1; HexHexCer d18:1/24:0; [M+H]$^{+}$	$C_{54}H_{103}NO_{13}$	7.20	974.7457	974.7507	-	-	-
LPC 18:1; [M+H]$^{+}$	$C_{26}H_{52}NO_7P$	1.41	522.3507	522.3559	0.19 ± 0.15	-	-
LPE 12:0; [M+H]$^{+}$	$C_{17}H_{36}NO_7P$	0.66	398.2345	398.2308	0.34 ± 0.19	0.34 ± 0.24	✓
LPE 13:0; [M+H]$^{+}$	$C_{18}H_{38}NO_7P$	0.76	412.2460	412.2464	1.39 ± 0.67	1.54 ± 0.76	✓
LPE 14:0; [M+H]$^{+}$	$C_{19}H_{40}NO_7P$	0.90	426.2610	426.2621	2.15 ± 1.19	2.35 ± 1.27	✓

LPE 14:1e; [M-H]$^-$	$C_{19}H_{40}NO_6P$	1.09	408.2481	408.2521	-	0.57 ± 0.64	-
LPE 15:0; [M-H]$^-$	$C_{20}H_{42}NO_7P$	1.13	438.2596	438.2626	-	3.64 ± 1.36	-
LPE 15:1; [M+H]$^+$	$C_{20}H_{40}NO_7P$	0.84	438.2628	438.2621	0.35 ± 0.16	0.37 ± 0.12	✓
LPE 15:1e; [M+H]$^+$	$C_{20}H_{42}NO_6P$	1.30	424.2815	424.2828	4.88 ± 2.75	4.70 ± 2.89	✓
LPE 16:0; [M+H]$^+$	$C_{21}H_{44}NO_7P$	1.42	454.2922	454.2933	6.86 ± 3.93	7.57 ± 4.07	✓
LPE 16:1; [M+H]$^+$	$C_{21}H_{42}NO_7P$	1.01	452.2746	452.2777	4.03 ± 2.1	3.76 ± 1.82	✓
LPE 16:1e; [M+H]$^+$	$C_{21}H_{44}NO_6P$	1.63	438.2986	438.2984	11.1 ± 10.0	10.8 ± 10.0	✓
LPE 16:2e; [M+H]$^+$	$C_{21}H_{42}NO_6P$	1.21	436.2815	436.2828	2.65 ± 3.33	2.57 ± 3.14	✓
LPE 17:0; [M-H]$^-$	$C_{22}H_{46}NO_7P$	1.72	466.2907	466.2939	-	2.40 ± 1.17	-
LPE 17:1; [M+H]$^+$	$C_{22}H_{44}NO_7P$	1.19	466.2939	466.2933	6.72 ± 3.04	6.98 ± 2.81	✓
LPE 17:1e; [M+H]$^+$	$C_{22}H_{46}NO_6P$	1.98	452.3100	452.3141	1.89 ± 1.16	-	-
LPE 17:2e; [M-H]$^-$	$C_{22}H_{44}NO_6P$	1.48	448.2799	448.2833	-	1.77 ± 1.13	-
LPE 18:0; [M-H]$^-$	$C_{23}H_{48}NO_7P$	2.18	480.3080	480.3096	-	1.14 ± 0.43	-
LPE 18:1; [M+H]$^+$	$C_{23}H_{46}NO_7P$	1.45	480.3073	480.3090	3.02 ± 1.48	3.85 ± 2.48	✓
LPE 18:1e; [M-H]$^-$	$C_{23}H_{48}NO_6P$	2.48	464.3127	464.3147		0.67 ± 0.54	-
LPE 18:2e; [M+H]$^+$	$C_{23}H_{46}NO_6P$	1.78	464.3127	464.3141	1.81 ± 1.84	1.82 ± 1.81	✓
MGDG 26:0; MGDG 12:0-14:0; [M+HCOO]$^-$	$C_{35}H_{66}O_{10}$	4.05	691.4645	691.4638	-	-	-
MGDG 28:0; MGDG 12:0-16:0; [M+NH$_4$]$^+$	$C_{37}H_{70}O_{10}$	4.52	692.5338	692.5312	-	-	✓
MGDG 28:1e; MGDG 16:1e/12:0; [M+HCOO]$^-$	$C_{37}H_{70}O_9$	4.93	703.4965	703.5002	-	-	-
MGDG 29:1e; MGDG 16:1e/13:0; [M+HCOO]$^-$	$C_{38}H_{72}O_9$	5.23	717.5109	717.5158	-	-	-
MGDG 30:0; MGDG 14:0-16:0; [M+NH$_4$]$^+$	$C_{39}H_{74}O_{10}$	5.15	720.5605	720.5625	-	-	✓
MGDG 30:1; MGDG 12:0-18:1; [M+NH$_4$]$^+$	$C_{39}H_{72}O_{10}$	4.67	718.5435	718.5469	-	-	-
MGDG 30:1e; MGDG 16:1e/14:0; [M+HCOO]$^-$	$C_{39}H_{74}O_9$	5.60	731.5281	731.5315	-	-	-
MGDG 31:0; [M+NH$_4$]$^+$	$C_{40}H_{76}O_{10}$	5.45	734.5720	734.5782	-	-	✓
MGDG 31:2e; MGDG 17:2e/14:0; [M+HCOO]$^-$	$C_{40}H_{74}O_9$	5.32	743.5311	743.5315	-	-	-
MGDG 32:0; MGDG 16:0-16:0; [M+NH$_4$]$^+$	$C_{41}H_{78}O_{10}$	5.79	748.5918	748.5938	-	-	✓
MGDG 32:1; MGDG 14:0-18:1; [M+NH$_4$]$^+$	$C_{41}H_{76}O_{10}$	5.25	746.5783	746.5782	-	-	✓
MGDG 32:1e; MGDG 16:1e/16:0; [M+HCOO]$^-$	$C_{41}H_{78}O_9$	6.25	759.5630	759.5628	-	-	-
MGDG 32:2e; MGDG 18:2e/14:0; [M+HCOO]$^-$	$C_{41}H_{76}O_9$	5.65	757.5476	757.5471	-	-	-
MGDG 33:0; MGDG 16:0-17:0; [M+NH$_4$]$^+$	$C_{42}H_{80}O_{10}$	6.11	762.6077	762.6095	-	-	✓
MGDG 33:1; MGDG 15:0-18:1; [M+NH$_4$]$^+$	$C_{42}H_{78}O_{10}$	5.53	760.5908	760.5938	-	-	✓
MGDG 33:1e; MGDG 17:1e/16:0; [M+HCOO]$^-$	$C_{42}H_{80}O_9$	6.55	773.5760	773.5784	-	-	-
MGDG 33:2e; MGDG 18:2e/15:0; [M+HCOO]$^-$	$C_{42}H_{78}O_9$	6.00	771.5538	771.5628	-	-	-
MGDG 34:0; MGDG 16:0-18:0; [M+NH$_4$]$^+$	$C_{43}H_{82}O_{10}$	6.46	776.6257	776.6251	-	-	✓

MGDG 34:1; MGDG 16:0-18:1; $[M+NH_4]^+$	$C_{43}H_{80}O_{10}$	5.84	774.6078	774.6095	-	-	✓
MGDG 34:2; MGDG 16:1-18:1; $[M+NH_4]^+$	$C_{43}H_{78}O_{10}$	5.33	772.5945	772.5938	-	-	-
MGDG 34:2e; MGDG 18:2e/16:0; $[M+HCOO]^-$	$C_{43}H_{80}O_9$	6.30	785.5767	785.5784	-	-	-
MGDG 35:1; MGDG 17:0-18:1; $[M+NH_4]^+$	$C_{44}H_{82}O_{10}$	6.20	788.6209	788.6251	-	-	✓
MGDG 35:2; MGDG 17:1-18:1; $[M+HCOO]^-$	$C_{44}H_{80}O_{10}$	5.64	813.5728	813.5734	-	-	-
MGDG 35:2e; MGDG 19:2e/16:0; $[M+HCOO]^-$	$C_{44}H_{82}O_9$	6.69	799.5913	799.5941	-	-	-
MGDG 36:0; MGDG 18:0-18:0; $[M+NH_4]^+$	$C_{45}H_{86}O_{10}$	7.11	804.6561	804.6564	-	-	✓
MGDG 36:1; MGDG 18:0-18:1; $[M+NH_4]^+$	$C_{45}H_{84}O_{10}$	6.49	802.6369	802.6408	-	-	✓
MGDG 36:2; MGDG 18:1-18:1; $[M+NH_4]^+$	$C_{45}H_{82}O_{10}$	5.91	800.6244	800.6251	-	-	✓
MGDG 37:1; MGDG 17:0-20:1; $[M+NH_4]^+$	$C_{46}H_{86}O_{10}$	6.82	816.6509	816.6564	-	-	-
MGDG 38:1; MGDG 18:0-20:1; $[M+NH_4]^+$	$C_{47}H_{88}O_{10}$	7.14	830.6732	830.6721	-	-	✓
MGDG 38:2; MGDG 18:1-20:1; $[M+NH_4]^+$	$C_{47}H_{86}O_{10}$	6.52	828.6534	828.6564	-	-	-
PC 30:1; $[M+H]^+$	$C_{38}H_{74}NO_8P$	4.18	704.5214	704.5230	0.24 ± 0.36	-	-
PC 32:0; PC 16:0-16:0; $[M+H]^+$	$C_{40}H_{80}NO_8P$	5.28	734.5716	734.5699	1.62 ± 0.97	-	-
PC 32:1e; $[M+H]^+$	$C_{40}H_{80}NO_7P$	5.58	718.5761	718.5750	6.55 ± 3.71	6.08 ± 3.14	✓
PC 32:2e; $[M+H]^+$	$C_{40}H_{78}NO_7P$	5.04	716.5741	716.5594	5.16 ± 3.29	-	-
PC 34:0e; $[M+H]^+$	$C_{42}H_{86}NO_7P$	6.34	748.6245	748.6220	0.42 ± 0.42	-	-
PC 34:1; PC 16:0-18:1; $[M+H]^+$	$C_{42}H_{82}NO_8P$	5.35	760.5900	760.5856	5.79 ± 5.59	4.78 ± 4.23	✓
PC 34:1e; PC 18:0e/16:1; $[M+HCOO]^-$	$C_{42}H_{84}NO_7P$	5.72	790.5916	790.5968		2.77 ± 2.95	-
PC 34:2; $[M+H]^+$	$C_{42}H_{80}NO_8P$	4.86	758.5725	758.5699	3.27 ± 4.55	3.15 ± 3.98	✓
PC 34:3e; $[M+H]^+$	$C_{42}H_{80}NO_7P$	5.14	742.5790	742.5750	1.90 ± 1.88	2.20 ± 1.72	✓
PC 35:7e; $[M+H]^+$	$C_{43}H_{74}NO_7P$	4.85	748.5309	748.5281	0.37 ± 0.10	-	-
PC 36:1; $[M+H]^+$	$C_{44}H_{86}NO_8P$	6.00	788.6179	788.6169	2.19 ± 2.02	2.32 ± 1.88	✓
PC 36:2; $[M+H]^+$	$C_{44}H_{84}NO_8P$	5.50	786.5988	786.6012	1.93 ± 2.22	-	-
PC 36:2e; $[M+H]^+$	$C_{44}H_{86}NO_7P$	6.29	772.6243	772.6220	1.08 ± 0.79	-	-
PC 36:3; PC 18:1-18:2; $[M+H]^+$	$C_{44}H_{82}NO_8P$	4.93	784.5826	784.5856	0.97 ± 1.13	-	-
PC 36:3e; $[M+H]^+$	$C_{44}H_{84}NO_7P$	5.70	770.6071	770.6063	0.85 ± 0.81	-	-
PC 36:4; $[M+H]^+$	$C_{44}H_{80}NO_8P$	4.48	782.5653	782.5699	0.25 ± 0.31	-	-
PC 36:5e; $[M+H]^+$	$C_{44}H_{80}NO_7P$	5.62	766.5673	766.5750	0.90 ± 0.58	-	-
PC 36:7e; $[M+H]^+$	$C_{44}H_{76}NO_7P$	5.16	762.5517	762.5437	0.39 ± 0.16	-	-
PC 37:7e; $[M+H]^+$	$C_{45}H_{78}NO_7P$	5.46	776.5631	776.5594	1.83 ± 0.63	-	-
PC 38:4; $[M+H]^+$	$C_{46}H_{84}NO_8P$	5.46	810.6085	810.6012	0.19 ± 0.26	-	-
PC 39:8e; $[M+H]^+$	$C_{47}H_{80}NO_7P$	5.54	802.5850	802.5750	0.53 ± 0.16	-	-
PC 42:2e; $[M+H]^+$	$C_{50}H_{98}NO_7P$	7.73	856.7180	856.7159	0.10 ± 0.10	-	-
PC 42:4e; $[M+H]^+$	$C_{50}H_{94}NO_7P$	7.18	852.6864	852.6846	0.04 ± 0.04	-	-

PE 24:0; PE 12:0-12:0; $[M+H]^+$	$C_{29}H_{58}NO_8P$	3.23	580.3985	580.3978	2.68 ± 0.86	-	✓
PE 25:0; $[M+H]^+$	$C_{30}H_{60}NO_8P$	3.45	594.4138	594.4135	4.62 ± 1.84	-	✓
PE 26:0; PE 13:0-13:0; $[M+H]^+$	$C_{31}H_{62}NO_8P$	3.68	608.4328	608.4291	25.0 ± 9.7	-	✓
PE 26:1; $[M+H]^+$	$C_{31}H_{60}NO_8P$	3.33	606.4132	606.4135	1.42 ± 0.53	-	-
PE 26:1e; PE 15:1e/11:0; $[M+H]^+$	$C_{31}H_{62}NO_7P$	3.92	592.4348	592.4342	21.7 ± 5.1	-	✓
PE 27:0; PE 13:0-14:0; $[M+H]^+$	$C_{32}H_{64}NO_8P$	3.93	622.4463	622.4448	26.3 ± 11.9	-	✓
PE 27:1; $[M+H]^+$	$C_{32}H_{62}NO_8P$	3.57	620.4280	620.4291	3.17 ± 1.6	-	✓
PE 27:1e; PE 15:1e/12:0; $[M+H]^+$	$C_{32}H_{64}NO_7P$	4.19	606.4514	606.4498	27.3 ± 13.7	-	✓
PE 28:0; PE 13:0-15:0; $[M+H]^+$	$C_{33}H_{66}NO_8P$	4.21	636.4627	636.4604	94.0 ± 47.5	-	✓
PE 28:1; PE 12:0-16:1; $[M+H]^+$	$C_{33}H_{64}NO_8P$	3.78	634.4467	634.4448	14.4 ± 6.0	-	✓
PE 28:1e; PE 15:1e/13:0; $[M+H]^+$	$C_{33}H_{66}NO_7P$	4.49	620.4638	620.4655	179.4 ± 61.4	-	✓
PE 28:2e; PE 16:2e/12:0; $[M+H]^+$	$C_{33}H_{64}NO_7P$	4.03	618.4517	618.4498	24.2 ± 5.0	-	✓
PE 29:0; PE 14:0-15:0; $[M+H]^+$	$C_{34}H_{68}NO_8P$	4.50	650.4805	650.4761	49.2 ± 21.2	-	✓
PE 29:1; PE 13:0-16:1; $[M+H]^+$	$C_{34}H_{66}NO_8P$	4.04	648.4604	648.4604	15.7 ± 5.4	-	✓
PE 29:1e; PE 15:1e/14:0; $[M+H]^+$	$C_{34}H_{68}NO_7P$	4.78	634.4822	634.4811	61.1 ± 41.7	-	✓
PE 29:2e; PE 16:2e/13:0; $[M+H]^+$	$C_{34}H_{66}NO_7P$	4.31	632.4659	632.4655	31.9 ± 13.5	-	✓
PE 30:0; PE 14:0-16:0; $[M+H]^+$	$C_{35}H_{70}NO_8P$	4.80	664.4946	664.4917	84.5 ± 37.9	-	✓
PE 30:1; PE 14:0-16:1; $[M+H]^+$	$C_{35}H_{68}NO_8P$	4.31	662.4819	662.4761	68.9 ± 31.9	-	✓
PE 30:1e; PE 16:1e/14:0; $[M+H]^+$	$C_{35}H_{70}NO_7P$	5.12	648.4958	648.4968	115.7 ± 25.3	-	✓
PE 30:2; PE 15:1-15:1; $[M+H]^+$	$C_{35}H_{66}NO_8P$	3.95	660.4614	660.4604	4.95 ± 1.56	-	✓
PE 30:2e; PE 16:2e/14:0; $[M+H]^+$	$C_{35}H_{68}NO_7P$	4.61	646.4829	646.4811	197.4 ± 46.1	-	✓
PE 30:3e; PE 16:2e/14:1; $[M+H]^+$	$C_{35}H_{66}NO_7P$	4.22	644.4726	644.4655	8.78 ± 2.78	-	-
PE 31:0; PE 15:0-16:0; $[M+H]^+$	$C_{36}H_{72}NO_8P$	5.03	678.5112	678.5074	38.3 ± 25.8	-	✓
PE 31:1; PE 15:0-16:1; $[M+H]^+$	$C_{36}H_{70}NO_8P$	4.61	676.4957	676.4917	85.1 ± 26.9	-	✓
PE 31:1e; PE 16:1e/15:0; $[M-H]^-$	$C_{36}H_{72}NO_7P$	5.43	660.4968	660.4974		-	-
PE 31:2; $[M+H]^+$	$C_{36}H_{68}NO_8P$	4.20	674.4795	674.4761	8.81 ± 3.08	-	✓
PE 31:2e; PE 15:1e/16:1; $[M+H]^+$	$C_{36}H_{70}NO_7P$	4.91	660.4976	660.4968	154.2 ± 68.3	-	✓
PE 31:3e; PE 16:2e/15:1; $[M+H]^+$	$C_{36}H_{68}NO_7P$	4.45	658.4838	658.4811	15.7 ± 5.6	-	✓
PE 32:0; PE 16:0-16:0; $[M+H]^+$	$C_{37}H_{74}NO_8P$	5.45	692.5227	692.5230	36.3 ± 16.1	-	✓
PE 32:1; PE 15:0-17:1; $[M+H]^+$	$C_{37}H_{72}NO_8P$	4.89	690.5115	690.5074	338.1 ± 211.6	-	✓
PE 32:1e; PE 16:1e/16:0; $[M+H]^+$	$C_{37}H_{74}NO_7P$	5.79	676.5284	676.5281	52.1 ± 16.0	-	✓
PE 32:2; PE 16:1-16:1; $[M+H]^+$	$C_{37}H_{70}NO_8P$	4.44	688.4930	688.4917	63.9 ± 13.3	-	✓
PE 32:2e; PE 15:1e/17:1; $[M+H]^+$	$C_{37}H_{72}NO_7P$	5.20	674.5161	674.5124	656.1 ± 166.8	-	✓
PE 32:3e; PE 16:2e/16:1; $[M+H]^+$	$C_{37}H_{70}NO_7P$	4.72	672.4976	672.4968	215.4 ± 73.1	-	✓

PE 33:0; $[M+H]^+$	$C_{38}H_{76}NO_8P$	5.69	706.5314	706.5387	8.57 ± 6.89	-	✓
PE 33:1; PE 15:0-18:1; $[M+H]^+$	$C_{38}H_{74}NO_8P$	5.22	704.5222	704.5230	182.5 ± 63.8	-	✓
PE 33:1e; PE 17:1e/16:0; $[M+H]^+$	$C_{38}H_{76}NO_7P$	6.11	690.5411	690.5437	10.8 ± 8.8	-	✓
PE 33:2; PE 16:1-17:1; $[M+H]^+$	$C_{38}H_{72}NO_8P$	4.70	702.5046	702.5074	120.4 ± 42	-	✓
PE 33:2e; PE 16:1e/17:1; $[M+H]^+$	$C_{38}H_{74}NO_7P$	5.52	688.5303	688.5281	249.5 ± 141.5	-	✓
PE 33:3e; PE 16:2e/17:1; $[M+H]^+$	$C_{38}H_{72}NO_7P$	5.00	686.5108	686.5124	158.4 ± 51.2	-	-
PE 34:0; $[M+H]^+$	$C_{39}H_{78}NO_8P$	6.12	720.5465	720.5543	6.44 ± 1.67	-	✓
PE 34:1; PE 16:0-18:1; $[M+H]^+$	$C_{39}H_{76}NO_8P$	5.52	718.5433	718.5387	224.2 ± 53.3	-	✓
PE 34:2; PE 17:1-17:1; $[M+H]^+$	$C_{39}H_{74}NO_8P$	4.99	716.5295	716.5230	213.4 ± 114.8	-	✓
PE 34:2e; PE 16:1e/18:1; $[M+H]^+$	$C_{39}H_{76}NO_7P$	5.86	702.5455	702.5437	305.4 ± 95.8	-	✓
PE 34:3; $[M+H]^+$	$C_{39}H_{72}NO_8P$	4.64	714.5111	714.5074	4.06 ± 5.21	-	-
PE 34:3e; PE 17:2e/17:1; $[M+H]^+$	$C_{39}H_{74}NO_7P$	5.30	700.5325	700.5281	446.9 ± 143.4	-	✓
PE 35:1; PE 17:0-18:1; $[M+H]^+$	$C_{40}H_{78}NO_8P$	5.82	732.5508	732.5543	46.5 ± 32.4	-	✓
PE 35:2; PE 17:1-18:1; $[M+H]^+$	$C_{40}H_{76}NO_8P$	5.28	730.5375	730.5387	77.2 ± 30.9	-	-
PE 35:2e; PE 17:1e/18:1; $[M+H]^+$	$C_{40}H_{78}NO_7P$	6.19	716.5585	716.5594	29.0 ± 25.3	-	✓
PE 35:3e; PE 17:2e/18:1; $[M+H]^+$	$C_{40}H_{76}NO_7P$	5.60	714.5439	714.5437	81.7 ± 40.3	-	✓
PE 36:0; $[M+H]^+$	$C_{41}H_{82}NO_8P$	6.79	748.5908	748.5856	0.79 ± 0.83	-	-
PE 36:1; PE 18:0-18:1; $[M+H]^+$	$C_{41}H_{80}NO_8P$	6.19	746.5701	746.5699	20.8 ± 7.1	-	✓
PE 36:2; PE 18:1-18:1; $[M+H]^+$	$C_{41}H_{78}NO_8P$	5.58	744.5559	744.5543	65.1 ± 19.1	-	✓
PE 36:2e; PE 18:1e/18:1; $[M+H]^+$	$C_{41}H_{80}NO_7P$	6.52	730.5729	730.5750	12.7 ± 6.4	-	-
PE 36:3e; PE 18:2e/18:1; $[M+H]^+$	$C_{41}H_{78}NO_7P$	5.91	728.5577	728.5594	62.4 ± 23.3	-	✓
PE 36:5e; PE 16:1e/20:4; $[M-H]^-$	$C_{41}H_{74}NO_7P$	5.22	722.5035	722.5130	-	-	-
PE 37:2; $[M+H]^+$	$C_{42}H_{80}NO_8P$	5.90	758.5695	758.5699	5.34 ± 5.87	-	-
PE 38:1; $[M+H]^+$	$C_{43}H_{84}NO_8P$	6.81	774.6108	774.6012	1.15 ± 0.74	-	-
PE-Cer t33:1; PE-Cer t18:1/15:0; $[M-H]^-$	$C_{35}H_{71}N_2O_7P$	3.93	661.4885	661.4926	-	-	-
PE-Cer t34:0; PE-Cer t18:0/16:0; $[M-H]^-$	$C_{36}H_{75}N_2O_7P$	4.28	677.5208	677.5239	-	-	-
PE-Cer t34:1; PE-Cer t19:1/15:0; $[M-H]^-$	$C_{36}H_{73}N_2O_7P$	4.18	675.5044	675.5083	-	-	-
PE-Cer t35:0; PE-Cer t18:0/17:0; $[M-H]^-$	$C_{37}H_{77}N_2O_7P$	4.56	691.5377	691.5396	-	-	-
PE-Cer t35:1; PE-Cer t21:0/14:1; $[M-H]^-$	$C_{37}H_{75}N_2O_7P$	4.43	689.5190	689.5239	-	-	-
PE-Cer t36:0; PE-Cer t19:0/17:0; $[M-H]^-$	$C_{38}H_{79}N_2O_7P$	4.82	705.5521	705.5552	-	-	-
PE-Cer t36:1; PE-Cer t20:1/16:0; $[M-H]^-$	$C_{38}H_{77}N_2O_7P$	4.70	703.5391	703.5396	-	-	-
PG 27:0; PG 12:0-15:0; $[M-H]^-$	$C_{33}H_{65}O_{10}P$	3.46	651.4179	651.4243	-	0.86 ± 0.50	-
PG 28:0; PG 14:0-14:0; $[M+NH_4]^+$	$C_{34}H_{67}O_{10}P$	3.65	684.4782	684.4815	2.89 ± 1.06	3.04 ± 0.99	✓
PG 29:0; $[M+NH_4]^+$	$C_{35}H_{69}O_{10}P$	3.83	698.4899	698.4972	4.00 ± 2.46	5.64 ± 4.00	✓

PG 30:0; PG 14:0-16:0; $[M+NH_4]^+$	$C_{36}H_{71}O_{10}P$	4.12	712.5117	712.5128	6.18 ± 4.17	-	-
PG 30:1; $[M+NH_4]^+$	$C_{36}H_{69}O_{10}P$	3.73	710.4949	710.4972	3.17 ± 1.82	-	-
PG 31:0; PG 15:0-16:0; $[M+NH_4]^+$	$C_{37}H_{73}O_{10}P$	4.33	726.5245	726.5285	11.9 ± 7.8	13.7 ± 10.6	✓
PG 31:1; $[M+NH_4]^+$	$C_{37}H_{71}O_{10}P$	3.93	724.5107	724.5128	6.86 ± 3.53	-	-
PG 32:0; PG 14:0-18:0; $[M+NH_4]^+$	$C_{38}H_{75}O_{10}P$	4.67	740.5414	740.5441	19.2 ± 11.1	13.4 ± 5.7	✓
PG 32:1; PG 16:0-16:1; $[M+NH_4]^+$	$C_{38}H_{73}O_{10}P$	4.20	738.5335	738.5285	29.2 ± 16.5	32.6 ± 17.4	✓
PG 32:2; PG 16:1-16:1; $[M+NH_4]^+$	$C_{38}H_{71}O_{10}P$	3.80	736.5156	736.5128	2.72 ± 1.41	2.67 ± 1.22	✓
PG 33:1; PG 16:0-17:1; $[M+NH_4]^+$	$C_{39}H_{75}O_{10}P$	4.43	752.5438	752.5441	24.2 ± 9.0	22.4 ± 9.8	✓
PG 33:2; $[M+NH_4]^+$	$C_{39}H_{73}O_{10}P$	4.04	750.5264	750.5285	2.42 ± 2.01	-	-
PG 34:1; PG 16:0-18:1; $[M+NH_4]^+$	$C_{40}H_{77}O_{10}P$	4.72	766.5612	766.5598	86.4 ± 43.3	44.0 ± 18.3	✓
PG 34:2; PG 17:1-17:1; $[M+NH_4]^+$	$C_{40}H_{75}O_{10}P$	4.28	764.5390	764.5441	8.88 ± 2.74	11.1 ± 3.2	✓
PG 35:1; PG 18:0-17:1; $[M+NH_4]^+$	$C_{41}H_{79}O_{10}P$	4.96	780.5741	780.5754	13.5 ± 8.3	9.78 ± 3.43	✓
PG 35:2; PG 17:1-18:1; $[M+NH_4]^+$	$C_{41}H_{77}O_{10}P$	4.53	778.5579	778.5598	4.54 ± 2.26	6.79 ± 3.77	✓
PG 36:1; PG 18:0-18:1; $[M+NH_4]^+$	$C_{42}H_{81}O_{10}P$	5.30	794.5896	794.5911	9.49 ± 6.37	10.2 ± 3.6	✓
PG 36:2; PG 18:1-18:1; $[M+NH_4]^+$	$C_{42}H_{79}O_{10}P$	4.78	792.5756	792.5754	18.5 ± 6.8	15 ± 4.74	✓
SM d32:1; $[M+H]^+$	$C_{37}H_{75}N_2O_6P$	3.98	675.5415	675.5441	1.03 ± 0.83	-	-
SM d33:1; $[M+H]^+$	$C_{38}H_{77}N_2O_6P$	4.27	689.5552	689.5597	0.80 ± 0.68	-	-
SM d34:0;$[M+H]^+$	$C_{39}H_{81}N_2O_6P$	4.80	705.5915	705.5910	19.8 ± 15.6	30.5 ± 27.6	✓
SM d34:1; $[M+H]^+$	$C_{39}H_{79}N_2O_6P$	4.57	703.5752	703.5754	35.9 ± 27.9	72.2 ± 64.9	✓
SM d34:2; $[M+H]^+$	$C_{39}H_{77}N_2O_6P$	4.08	701.5607	701.5597	0.40 ± 0.31	-	-
SM d36:0; $[M+H]^+$	$C_{41}H_{85}N_2O_6P$	5.46	733.6223	733.6223	4.70 ± 3.70	-	-
SM d36:1; $[M+H]^+$	$C_{41}H_{83}N_2O_6P$	5.22	731.6050	731.6067	4.37 ± 3.59	-	-
SM d38:0; $[M+H]^+$	$C_{43}H_{89}N_2O_6P$	6.15	761.6520	761.6536	2.16 ± 1.78	-	-
SM d38:1; $[M+H]^+$	$C_{43}H_{87}N_2O_6P$	5.90	759.6325	759.6380	1.96 ± 1.5ß	-	-
SM d40:0; $[M+H]^+$	$C_{45}H_{93}N_2O_6P$	6.83	789.6822	789.6849	2.13 ± 1.78	-	-
SM d40:1; $[M+H]^+$	$C_{45}H_{91}N_2O_6P$	6.59	787.6657	787.6693	2.18 ± 1.64	-	-
SM d41:1; $[M+H]^+$	$C_{46}H_{93}N_2O_6P$	6.92	801.6807	801.6849	0.44 ± 0.34	-	-
SM d41:2; $[M+H]^+$	$C_{46}H_{91}N_2O_6P$	6.23	799.6604	799.6693	0.08 ± 0.08	-	-
SM d42:0; $[M+H]^+$	$C_{47}H_{97}N_2O_6P$	7.49	817.7119	817.7162	2.59 ± 2.16	5.0 ± 4.8	✓
SM d42:1; $[M+H]^+$	$C_{47}H_{95}N_2O_6P$	7.26	815.6998	815.7006	3.63 ± 2.74	-	-
SM d42:2; $[M+H]^+$	$C_{47}H_{93}N_2O_6P$	6.56	813.6884	813.6849	3.61 ± 3.82	-	-
SM d44:0; $[M+H]^+$	$C_{49}H_{101}N_2O_6P$	8.13	845.7396	845.7475	0.64 ± 0.58	-	-
SM d44:1; $[M+H]^+$	$C_{49}H_{99}N_2O_6P$	7.90	843.7310	843.7319	1.06 ± 0.86	-	-
SM d44:2; $[M+H]^+$	$C_{49}H_{97}N_2O_6P$	7.23	841.7175	841.7162	0.28 ± 0.27	-	-

TAG 24:0; TAG 8:0-8:0-8:0; $[M+NH_4]^+$	$C_{27}H_{50}O_6$	3.83	488.3966	488.3951	1.30 ± 1.29	-	-
TAG 26:0; TAG 8:0-8:0-10:0; $[M+NH_4]^+$	$C_{29}H_{54}O_6$	4.32	516.4265	516.4264	4.51 ± 4.74	-	-
TAG 28:0; TAG 8:0-10:0-10:0; $[M+NH_4]^+$	$C_{31}H_{58}O_6$	4.91	544.4582	544.4577	13.4 ± 20.2	-	-
TAG 30:0; TAG 10:0-10:0-10:0; $[M+NH_4]^+$	$C_{33}H_{62}O_6$	5.64	572.4913	572.4890	30.3 ± 58.7	-	-
TAG 32:0; TAG 8:0-12:0-12:0; $[M+NH_4]^+$	$C_{35}H_{66}O_6$	6.37	600.5229	600.5203	78.6 ± 180.0	-	-
TAG 34:0; TAG 8:0-10:0-16:0; $[M+NH_4]^+$	$C_{37}H_{70}O_6$	7.01	628.5524	628.5516	108.2 ± 224.2	-	-
TAG 36:0; TAG 10:0-12:0-14:0; $[M+NH_4]^+$	$C_{39}H_{74}O_6$	7.67	656.5850	656.5829	109.8 ± 237.7	-	-
TAG 38:0; TAG 10:0-12:0-16:0; $[M+NH_4]^+$	$C_{41}H_{78}O_6$	8.24	684.6200	684.6142	105.2 ± 186.0	-	-
TAG 38:1; TAG 8:0-12:0-18:1; $[M+NH_4]^+$	$C_{41}H_{76}O_6$	7.73	682.6028	682.5985	47.2 ± 92.9	-	-
TAG 39:0; TAG 8:0-15:0-16:0; $[M+NH_4]^+$	$C_{42}H_{80}O_6$	8.53	698.6299	698.6298	4.77 ± 8.33	-	-
TAG 40:0; TAG 10:0-14:0-16:0; $[M+NH_4]^+$	$C_{43}H_{82}O_6$	8.80	712.6498	712.6455	65.1 ± 111	-	-
TAG 40:1; TAG 10:0-12:0-18:1; $[M+NH_4]^+$	$C_{43}H_{80}O_6$	8.30	710.6331	710.6298	37.0 ± 58.4	-	-
TAG 40:2; TAG 8:0-14:0-18:2; $[M+NH_4]^+$	$C_{43}H_{78}O_6$	7.84	708.6154	708.6142	15.3 ± 26.7	-	-
TAG 41:0; TAG 10:0-15:0-16:0; $[M+NH_4]^+$	$C_{44}H_{84}O_6$	9.05	726.6646	726.6611	4.71 ± 7.29	-	-
TAG 41:1; TAG 8:0-16:0-17:1; $[M+NH_4]^+$	$C_{44}H_{82}O_6$	8.59	724.6471	724.6455	2.54 ± 4.37	-	-
TAG 42:0; TAG 12:0-14:0-16:0; $[M+NH_4]^+$	$C_{45}H_{86}O_6$	9.34	740.6777	740.6768	44.3 ± 64.8	-	-
TAG 42:1; TAG 8:0-16:0-18:1; $[M+NH_4]^+$	$C_{45}H_{84}O_6$	8.85	738.6676	738.6611	30.0 ± 40.8	-	-
TAG 42:2; TAG 8:0-16:1-18:1; $[M+NH_4]^+$	$C_{45}H_{82}O_6$	8.37	736.6488	736.6455	9.19 ± 13.49	-	-
TAG 42:3; TAG 10:0-14:0-18:3; $[M+NH_4]^+$	$C_{45}H_{80}O_6$	7.89	734.6289	734.6298	1.69 ± 2.88	-	-
TAG 43:0; TAG 10:0-15:0-18:0; $[M+NH_4]^+$	$C_{46}H_{88}O_6$	9.57	754.6967	754.6924	4.14 ± 5.45	-	-
TAG 43:1; TAG 10:0-15:0-18:1; $[M+NH_4]^+$	$C_{46}H_{86}O_6$	9.09	752.6831	752.6768	3.41 ± 4.57	-	-
TAG 44:0; TAG 12:0-14:0-18:0; $[M+NH_4]^+$	$C_{47}H_{90}O_6$	9.84	768.7141	768.7081	29.5 ± 38.6	-	-
TAG 44:1; TAG 10:0-16:0-18:1; $[M+NH_4]^+$	$C_{47}H_{88}O_6$	9.38	766.6976	766.6924	29.3 ± 37.0	-	-
TAG 44:2; TAG 10:0-16:0-18:2; $[M+NH_4]^+$	$C_{47}H_{86}O_6$	8.94	764.6759	764.6768	10.4 ± 12.9	-	-
TAG 44:3; TAG 8:0-16:1-20:2; $[M+NH_4]^+$	$C_{47}H_{84}O_6$	8.47	762.6647	762.6611	1.82 ± 2.36	-	-
TAG 45:0; TAG 14:0-15:0-16:0; $[M+NH_4]^+$	$C_{48}H_{92}O_6$	10.04	782.7264	782.7237	5.74 ± 6.08	-	-
TAG 45:1; TAG 10:0-17:0-18:1; $[M+NH_4]^+$	$C_{48}H_{90}O_6$	9.61	780.7152	780.7081	4.67 ± 4.40	-	-
TAG 45:2; TAG 13:0-15:1-17:1; $[M+NH_4]^+$	$C_{48}H_{88}O_6$	9.17	778.6987	778.6924	1.38 ± 1.29	-	-
TAG 46:0; TAG 14:0-16:0-16:0; $[M+NH_4]^+$	$C_{49}H_{94}O_6$	10.31	796.7457	796.7394	26.8 ± 35.6	-	-
TAG 46:1; TAG 14:0-16:0-16:1; $[M+NH_4]^+$	$C_{49}H_{92}O_6$	9.88	794.7286	794.7237	31.1 ± 37.3	-	-
TAG 46:2; TAG 12:0-16:0-18:2; $[M+NH_4]^+$	$C_{49}H_{90}O_6$	9.45	792.7139	792.7081	13.2 ± 14.3	-	-
TAG 46:3; TAG 10:0-18:1-18:2; $[M+NH_4]^+$	$C_{49}H_{88}O_6$	9.01	790.6979	790.6924	5.04 ± 4.24	-	-
TAG 47:0; TAG 15:0-16:0-16:0; $[M+NH_4]^+$	$C_{50}H_{96}O_6$	10.50	810.7570	810.7550	6.70 ± 5.77	-	-
TAG 47:1; TAG 14:0-16:0-17:1; $[M+NH_4]^+$	$C_{50}H_{94}O_6$	10.10	808.7329	808.7394	7.47 ± 6.25	-	-
TAG 47:2; TAG 16:0-14:1-17:1; $[M+NH_4]^+$	$C_{50}H_{92}O_6$	9.70	806.7259	806.7237	3.47 ± 2.19	-	-

TAG 48:0; TAG 14:0-16:0-18:0; $[M+NH_4]^+$	$C_{51}H_{98}O_6$	10.74	824.7770	824.7707	24.6 ± 26.1	-	-
TAG 48:1; TAG 14:0-16:0-18:1; $[M+NH_4]^+$	$C_{51}H_{96}O_6$	10.34	822.7611	822.7550	43.9 ± 54.4	-	-
TAG 48:3; TAG 12:0-18:1-18:2; $[M+NH_4]^+$	$C_{51}H_{92}O_6$	9.52	818.7244	818.7237	6.21 ± 6.28	-	-
TAG 49:0; TAG 14:0-17:0-18:0; $[M+NH_4]^+$	$C_{52}H_{100}O_6$	10.91	838.7947	838.7863	4.84 ± 4.42	-	-
TAG 49:1; TAG 15:0-16:0-18:1; $[M+NH_4]^+$	$C_{52}H_{98}O_6$	10.53	836.7739	836.7707	9.93 ± 8.57	-	-
TAG 49:2; TAG 16:0-16:1-17:1; $[M+NH_4]^+$	$C_{52}H_{96}O_6$	10.15	834.7492	834.7550	6.87 ± 2.90	-	-
TAG 49:3; TAG 16:0-15:1-18:2; $[M+NH_4]^+$	$C_{52}H_{94}O_6$	9.75	832.7369	832.7394	2.14 ± 1.95	-	-
TAG 50:0; TAG 16:0-16:0-18:0; $[M+NH_4]^+$	$C_{53}H_{102}O_6$	11.14	852.8027	852.8020	24.9 ± 41.8	-	-
TAG 50:1; TAG 14:0-18:0-18:1; $[M+NH_4]^+$	$C_{53}H_{100}O_6$	10.75	850.7939	850.7863	60.9 ± 61.0	-	-
TAG 50:2; TAG 16:0-16:1-18:1; $[M+NH_4]^+$	$C_{53}H_{98}O_6$	10.36	848.7751	848.7707	56.4 ± 56.4	-	-
TAG 50:3; TAG 14:0-18:1-18:2; $[M+NH_4]^+$	$C_{53}H_{96}O_6$	9.96	846.7587	846.7550	23.6 ± 29.0	-	-
TAG 50:4; TAG 16:1-16:1-18:2; $[M+NH_4]^+$	$C_{53}H_{94}O_6$	9.55	844.7406	844.7394	5.88 ± 10.44	-	-
TAG 51:2; TAG 15:0-18:1-18:1; $[M+NH_4]^+$	$C_{54}H_{100}O_6$	10.56	862.7872	862.7863	11.5 ± 9.6	-	-
TAG 51:3; TAG 15:0-18:1-18:2; $[M+NH_4]^+$	$C_{54}H_{98}O_6$	10.18	860.7748	860.7707	7.55 ± 9.17	-	-
TAG 51:4; TAG 15:1-18:1-18:2; $[M+NH_4]^+$	$C_{54}H_{96}O_6$	9.77	858.7567	858.7550	2.67 ± 5.40	-	-
TAG 52:0; TAG 16:0-18:0-18:0; $[M+NH_4]^+$	$C_{55}H_{106}O_6$	11.52	880.8361	880.8333	24.5 ± 61.3	-	-
TAG 52:1; TAG 16:0-18:0-18:1; $[M+NH_4]^+$	$C_{55}H_{104}O_6$	11.15	878.8196	878.8176	38.2 ± 49.8	-	-
TAG 52:2; TAG 16:0-18:1-18:1; $[M+NH_4]^+$	$C_{55}H_{102}O_6$	10.77	876.8054	876.8020	171.2 ± 281.1	-	-
TAG 52:3; TAG 16:0-18:1-18:2; $[M+NH_4]^+$	$C_{55}H_{100}O_6$	10.39	874.7935	874.7863	145.7 ± 301	-	-
TAG 52:4; TAG 16:0-18:2-18:2; $[M+NH_4]^+$	$C_{55}H_{98}O_6$	10.01	872.7756	872.7707	112.6 ± 288.3	-	-
TAG 52:5; TAG 16:1-18:2-18:2; $[M+NH_4]^+$	$C_{55}H_{96}O_6$	9.57	870.7535	870.7550	38.4 ± 112.4	-	-
TAG 52:6; TAG 18:1-18:2-16:3; $[M+NH_4]^+$	$C_{55}H_{94}O_6$	9.23	868.7467	868.7394	11.7 ± 36.7	-	-
TAG 52:8; TAG 18:2-16:3-18:3; $[M+NH_4]^+$	$C_{55}H_{90}O_6$	8.33	864.7147	864.7081	0.57 ± 1.15	-	-
TAG 53:0; TAG 14:0-18:0-21:0; $[M+NH_4]^+$	$C_{56}H_{108}O_6$	11.66	894.8477	894.8489	0.93 ± 0.88	-	-
TAG 53:1; TAG 17:0-18:0-18:1; $[M+NH_4]^+$	$C_{56}H_{106}O_6$	11.31	892.8362	892.8333	2.31 ± 1.94	-	-
TAG 53:2; TAG 17:0-18:1-18:1; $[M+NH_4]^+$	$C_{56}H_{104}O_6$	10.96	890.8187	890.8176	8.47 ± 10.37	-	-
TAG 53:3; TAG 17:1-18:1-18:1; $[M+NH_4]^+$	$C_{56}H_{102}O_6$	10.59	888.8076	888.8020	10.9 ± 18.9	-	-
TAG 53:4; TAG 17:1-18:1-18:2; $[M+NH_4]^+$	$C_{56}H_{100}O_6$	10.21	886.7903	886.7863	6.05 ± 11.7	-	-
TAG 54:0; TAG 16:0-18:0-20:0; $[M+NH_4]^+$	$C_{57}H_{110}O_6$	11.78	908.8720	908.8646	37.7 ± 107.3	-	-
TAG 54:1; TAG 18:0-18:0-18:1; $[M+NH_4]^+$	$C_{57}H_{108}O_6$	11.51	906.8425	906.8489	9.82 ± 15.25	-	-
TAG 54:2; TAG 18:0-18:1-18:1; $[M+NH_4]^+$	$C_{57}H_{106}O_6$	11.16	904.8370	904.8333	98.5 ± 234.7	-	-
TAG 54:3; TAG 18:0-18:1-18:2; $[M+NH_4]^+$	$C_{57}H_{104}O_6$	10.78	902.8228	902.8176	564.2 ± 1365.6	-	-
TAG 54:4; TAG 18:1-18:1-18:2; $[M+NH_4]^+$	$C_{57}H_{102}O_6$	10.42	900.8067	900.8020	452.7 ± 1241.9	-	-
TAG 54:5; TAG 18:1-18:2-18:2; $[M+NH_4]^+$	$C_{57}H_{100}O_6$	10.04	898.7928	898.7863	436.9 ± 1301.7	-	-
TAG 54:6; TAG 18:1-18:2-18:3; $[M+NH_4]^+$	$C_{57}H_{98}O_6$	9.65	896.7769	896.7707	277.8 ± 849.3	-	-

TAG 54:7; TAG 18:2-18:2-18:3; $[M+NH_4]^+$	$C_{57}H_{96}O_6$	9.28	894.7591	894.7550	90.5 ± 282.3	-	-
TAG 54:8; TAG 18:2-18:3-18:3; $[M+NH_4]^+$	$C_{57}H_{94}O_6$	8.90	892.7435	892.7394	11.4 ± 35.0	-	-
TAG 55:1; TAG 15:0-24:0-16:1; $[M+NH_4]^+$	$C_{58}H_{110}O_6$	11.68	920.8684	920.8646	0.95 ± 0.66	-	-
TAG 56:0; TAG 14:0-16:0-26:0; $[M+NH_4]^+$	$C_{59}H_{114}O_6$	11.92	936.9025	936.8959	3.46 ± 6.64	-	-
TAG 56:1; TAG 16:0-24:0-16:1; $[M+NH_4]^+$	$C_{59}H_{112}O_6$	11.79	934.8874	934.8802	3.32 ± 5.73	-	-
TAG 56:2; TAG 20:0-16:1-20:1; $[M+NH_4]^+$	$C_{59}H_{110}O_6$	11.53	932.8702	932.8646	25.9 ± 76.2	-	-
TAG 56:6; TAG 18:1-18:2-20:3; $[M+NH_4]^+$	$C_{59}H_{102}O_6$	10.16	924.8018	924.8020	6.70 ± 17.38	-	-
TAG 56:7; TAG 18:1-18:2-20:4; $[M+NH_4]^+$	$C_{59}H_{100}O_6$	9.90	922.7830	922.7863	2.24 ± 5.38	-	-
TAG 57:0; TAG 15:0-16:0-26:0; $[M+NH_4]^+$	$C_{60}H_{116}O_6$	11.96	950.9074	950.9115	1.08 ± 0.40	-	-
TAG 57:1; TAG 16:0-23:0-18:1; $[M+NH_4]^+$	$C_{60}H_{114}O_6$	11.86	948.8994	948.8959	1.17 ± 0.90	-	-
TAG 57:2; TAG 21:0-18:1-18:1; $[M+NH_4]^+$	$C_{60}H_{112}O_6$	11.70	946.8887	946.8802	1.29 ± 2.32	-	-
TAG 58:0; TAG 16:0-17:0-25:0; $[M+NH_4]^+$	$C_{61}H_{118}O_6$	12.00	964.9335	964.9272	1.63 ± 1.81	-	-
TAG 58:1; TAG 16:0-24:0-18:1; $[M+NH_4]^+$	$C_{61}H_{116}O_6$	11.92	962.9148	962.9115	3.89 ± 8.11	-	-
TAG 58:2; TAG 24:0-16:1-18:1; $[M+NH_4]^+$	$C_{61}H_{114}O_6$	11.79	960.9026	960.8959	13.6 ± 38.0	-	-
TAG 58:3; TAG 22:0-18:1-18:2; $[M+NH_4]^+$	$C_{61}H_{112}O_6$	11.55	958.8837	958.8802	11.3 ± 33.6	-	-
TAG 58:4; TAG 22:0-18:1-18:3; $[M+NH_4]^+$	$C_{61}H_{110}O_6$	11.24	956.8669	956.8646	7.27 ± 22.04	-	-
TAG 58:7; TAG 18:1-18:2-22:4; $[M+NH_4]^+$	$C_{61}H_{104}O_6$	10.26	950.8250	950.8176	0.35 ± 0.66	-	-
TAG 59:0; TAG 16:0-17:0-26:0; $[M+NH_4]^+$	$C_{62}H_{120}O_6$	12.03	978.9429	978.9428	0.82 ± 0.52	-	-
TAG 60:0; TAG 18:0-18:0-24:0; $[M+NH_4]^+$	$C_{63}H_{122}O_6$	12.07	992.9644	992.9585	1.20 ± 1.60	-	-
TAG 60:1; TAG 16:0-26:0-18:1; $[M+NH_4]^+$	$C_{63}H_{120}O_6$	12.00	990.9418	990.9428	1.94 ± 3.43	-	-
TAG 60:2; TAG 24:0-18:1-18:1; $[M+NH_4]^+$	$C_{63}H_{118}O_6$	11.92	988.9329	988.9272	5.77 ± 13.79	-	-
TAG 60:3; TAG 20:0-18:1-22:2; $[M+NH_4]^+$	$C_{63}H_{116}O_6$	11.79	986.9158	986.9115	8.14 ± 22.8	-	-
TAG 62:1; TAG 16:0-28:0-18:1; $[M+NH_4]^+$	$C_{65}H_{124}O_6$	12.06	1018.9819	1018.9741	0.97 ± 1.14	-	-
TAG 62:3; TAG 18:1-22:1-22:1; $[M+NH_4]^+$	$C_{65}H_{120}O_6$	11.92	1014.9473	1014.9428	2.50 ± 5.66	-	-

The method does not chromatographically resolve lipid species with identical total number of side chain carbons. The primary species that was identified by MS-DIAL is given if sufficient MS/MS information was present to reach the set identification threshold. Concentration values are given in ng per mg of dried sample. Although PE species were detected in negative mode, no quantification was conducted due to insufficient sensitivity of the corresponding surrogate calibrant in negative mode. 65.7% of all features that could be identified in negative mode were also detected in positive mode

Table S7 Comparison of *in vitro* biofilm versus *in vivo* plaque samples (BF vs. PL, foldchange <0.5, positive mode)

Identified lipid	Adduct type	t_R[min]	Measured m/z	*p* value	SGoF *p* value	Foldchange
CE 20:4	$[M+NH_4]^+$	10.05	690.6177	6.24E-03	1.27E-02	0.19
HBMP 46:2 (HBMP 14:0/16:1-16:1)	$[M+NH_4]^+$	6.57	946.7133	1.73E-03	3.52E-03	0.17
HexHexCer d34:1 (HexHexCer d18:1/16:0)	$[M+H]^+$	4.62	862.6298	3.63E-05	6.93E-05	0.06
HexHexCer d42:1 (HexHexCer d18:1/24:0)	$[M+H]^+$	7.24	974.7528	2.40E-03	4.97E-03	0.21
HexHexCer d42:2 (HexHexCer d18:1/24:1)	$[M+H]^+$	6.55	972.7360	1.37E-04	2.68E-04	0.08
LPC 16:0-sn1	$[M+H]^+$	1.39	496.3423	1.92E-06	4.30E-06	0.06
LPC 16:1e	$[M+H]^+$	1.58	480.3440	6.46E-03	1.39E-02	0.19
LPC 18:0	$[M+H]^+$	2.09	524.3699	1.90E-08	5.78E-08	0.03
LPC 18:1	$[M+H]^+$	1.40	522.3560	1.59E-05	2.91E-05	0.07
LPC 18:1e	$[M+H]^+$	1.77	508.3738	1.66E-05	2.96E-05	0.02
LPC 18:2	$[M+H]^+$	1.09	520.3403	9.98E-05	1.69E-04	0.07
LPC 20:0e	$[M+H]^+$	3.12	538.4218	7.82E-03	1.75E-02	0.09
LPC 22:1e	$[M+H]^+$	3.17	564.4395	1.67E-03	3.40E-03	0.04
LPE 18:1e	$[M+H]^+$	2.45	466.3304	2.60E-03	5.57E-03	0.14
LPE 22:2e	$[M+H]^+$	3.17	520.3790	6.92E-03	1.52E-02	0.18
PC 32:0 (PC 16:0-16:0)	$[M+H]^+$	5.27	734.5690	9.92E-04	1.92E-03	0.21
PC 32:0e	$[M+H]^+$	5.67	720.5899	6.96E-03	1.53E-02	0.30
PC 32:1	$[M+H]^+$	4.72	732.5584	1.13E-02	4.85E-02	0.25
PC 34:0	$[M+H]^+$	5.94	762.6024	1.04E-02	3.63E-02	0.38
PC 34:0e	$[M+H]^+$	6.35	748.6245	4.43E-03	8.82E-03	0.25
PC 34:1 (PC 16:0-18:1)	$[M+H]^+$	5.35	760.5861	7.94E-03	1.75E-02	0.33
PC 36:1	$[M+H]^+$	6.00	788.6210	3.70E-03	7.46E-03	0.32
PC 36:1e	$[M+H]^+$	6.41	774.6364	4.53E-03	9.57E-03	0.30
PC 36:2 (PC 18:1-18:1)	$[M+H]^+$	5.43	786.6022	8.29E-03	1.97E-02	0.28
PC 38:1e	$[M+H]^+$	7.03	802.6706	6.02E-03	1.25E-02	0.24
PC 38:2	$[M+H]^+$	6.03	814.6332	6.74E-04	1.25E-03	0.37
PC 38:2e	$[M+H]^+$	6.43	800.6498	7.65E-03	1.67E-02	0.30
PC 38:3	$[M+H]^+$	5.65	812.6176	9.85E-04	1.91E-03	0.23
PC 40:2e	$[M+H]^+$	7.07	828.6881	1.49E-03	2.98E-03	0.20
PC 40:5e	$[M+H]^+$	5.78	822.6379	6.43E-04	1.22E-03	0.20
PC 42:2e	$[M+H]^+$	7.69	856.7180	5.30E-03	1.08E-02	0.27

PC 42:3e	$[M+H]^+$	7.19	854.7003	1.56E-03	3.27E-03	0.23
PC 42:5e	$[M+H]^+$	6.44	850.6746	4.94E-04	9.56E-04	0.13
PC 44:5e	$[M+H]^+$	7.08	878.7053	8.93E-04	1.67E-03	0.14
PE 38:5e (PE 18:1e/20:4)	$[M+H]^+$	5.89	752.5632	6.42E-03	1.37E-02	0.33
SM d42:2	$[M+H]^+$	6.56	813.6884	8.78E-03	2.18E-02	0.23
SM d42:3	$[M+H]^+$	5.99	811.6671	5.93E-03	1.23E-02	0.29
TAG 50:1 (TAG 16:0-16:0-18:1)	$[M+Na]^+$	10.74	855.7423	7.43E-03	1.64E-02	0.47
TAG 52:1 (TAG 16:0-18:0-18:1)	$[M+Na]^+$	11.14	883.7736	7.81E-03	1.74E-02	0.31

Only identified lipids that show an SGoF adjusted p value (Mann-Whitney U test) below the significance level of $\alpha = 0.05$ are listed. Foldchanges are calculated based on median intensity values between experimental groups. Information about side chain composition is given in parentheses, if available. Here, a foldchange of <0.5 means more than factor 2 higher in PL samples

Table S8 Comparison of *in vitro* biofilm versus *in vivo* plaque samples (BF vs. PL, foldchange >2, positive mode)

Identified lipid	Adduct type	t_R[min]	Measured m/z	*p* value	SGoF *p* value	Foldchange
Cer-NS d35:2 (Cer-NS d18:1/17:1)	$[M+H]^+$	5.53	550.5154	2.78E-04	5.45E-04	9.18
Cer-NS d36:2 (Cer-NS d18:1/18:1)	$[M+H]^+$	5.86	564.5346	3.95E-03	7.98E-03	2.79
DAG 20:0 (DAG 8:0-12:0)	$[M+NH_4]^+$	3.14	418.3568	1.69E-04	3.49E-04	9.52
DAG 25:0 (DAG 10:0-15:0)	$[M+NH_4]^+$	4.39	488.4340	2.88E-05	5.32E-05	9.61
DAG 26:0 (DAG 12:0-14:0)	$[M+NH_4]^+$	4.68	502.4481	2.24E-03	4.70E-03	5.76
DAG 26:1 (DAG 8:0-18:1)	$[M+NH_4]^+$	4.20	500.4299	5.86E-03	1.23E-02	3.12
DAG 26:1e (DAG 13:1e/13:0)	$[M+NH_4]^+$	5.20	486.4505	3.91E-09	1.42E-08	12.8
DAG 27:0 (DAG 13:0-14:0)	$[M+NH_4]^+$	4.99	516.4616	2.11E-10	8.42E-10	57.3
DAG 27:1e (DAG 15:1e/12:0)	$[M+NH_4]^+$	5.54	500.4648	9.73E-09	3.02E-08	21.4
DAG 28:0e (DAG 14:0e/14:0)	$[M+NH_4]^+$	5.99	516.4966	6.86E-06	1.50E-05	19.1
DAG 28:1 (DAG 10:0-18:1)	$[M+NH_4]^+$	4.77	528.4675	1.31E-03	2.45E-03	7.10
DAG 28:1e (DAG 15:1e/13:0)	$[M+NH_4]^+$	5.89	514.4822	2.14E-08	6.19E-08	71.1
DAG 28:2e (DAG 17:2e/11:0)	$[M+NH_4]^+$	5.30	512.4689	8.13E-11	3.85E-10	48.8
DAG 29:0 (DAG 13:0-16:0)	$[M+NH_4]^+$	5.66	544.4944	3.08E-09	1.13E-08	33.6
DAG 29:1 (DAG 13:0-16:1)	$[M+NH_4]^+$	5.10	542.4778	4.50E-11	2.79E-10	37.9
DAG 29:1e (DAG 16:1e/13:0)	$[M+NH_4]^+$	6.24	528.4965	6.00E-09	1.94E-08	31.2
DAG 29:2e (DAG 16:2e/13:0)	$[M+NH_4]^+$	5.64	526.4820	2.48E-10	1.03E-09	54.5
DAG 30:0 (DAG 14:0-16:0)	$[M+NH_4]^+$	6.00	558.5118	2.52E-06	5.52E-06	14.9
DAG 30:1 (DAG 14:0-16:1)	$[M+NH_4]^+$	5.41	556.4970	5.64E-07	1.28E-06	20.6
DAG 30:2e (DAG 13:1e/17:1)	$[M+NH_4]^+$	5.98	540.4990	6.01E-14	2.01E-12	259.2
DAG 31:0 (DAG 15:0-16:0)	$[M+NH_4]^+$	6.24	572.5247	5.44E-08	1.94E-07	15.8
DAG 31:1 (DAG 14:0-17:1)	$[M+NH_4]^+$	5.75	570.5121	1.42E-09	5.45E-09	49.4
DAG 31:2 (DAG 15:1-16:1)	$[M+NH_4]^+$	5.26	568.4959	4.12E-06	8.45E-06	7.66
DAG 31:2e (DAG 15:1e/16:1)	$[M+NH_4]^+$	6.33	554.5126	3.18E-14	8.35E-13	261.9
DAG 31:3e (DAG 16:3e/15:0)	$[M+NH_4]^+$	5.77	552.5003	8.49E-11	3.88E-10	38.2
DAG 32:0 (DAG 16:0-16:0)	$[M+NH_4]^+$	6.70	586.5413	3.18E-06	7.11E-06	7.19
DAG 32:1 (DAG 16:0-16:1)	$[M+NH_4]^+$	6.08	584.5276	2.59E-08	8.40E-08	26.7
DAG 32:2 (DAG 15:1-17:1)	$[M+NH_4]^+$	5.54	582.5140	7.03E-09	2.26E-08	41.1
DAG 32:2e (DAG 16:2e/16:0)	$[M+NH_4]^+$	6.67	568.5280	3.94E-14	1.31E-12	929.4
DAG 32:3e (DAG 16:3e/16:0)	$[M+NH_4]^+$	6.10	566.5123	8.37E-14	2.78E-12	328.5
DAG 33:0 (DAG 16:0-17:0)	$[M+NH_4]^+$	7.04	600.5575	1.22E-07	4.30E-07	8.10

DAG 33:1 (DAG 16:0-17:1)	$[M+NH_4]^+$	6.41	598.5387	2.73E-11	1.83E-10	44.5
DAG 33:2 (DAG 16:1-17:1)	$[M+NH_4]^+$	5.85	596.5227	2.78E-12	3.17E-11	111.2
DAG 33:3e (DAG 16:2e/17:1)	$[M+NH_4]^+$	6.41	580.5305	3.87E-14	1.23E-12	459.8
DAG 34:0 (DAG 16:0-18:0)	$[M+NH_4]^+$	7.37	614.5714	2.91E-05	5.42E-05	4.54
DAG 34:1 (DAG 16:0-18:1)	$[M+NH_4]^+$	6.74	612.5538	1.14E-07	3.91E-07	11.1
DAG 34:2 (DAG 17:1-17:1)	$[M+NH_4]^+$	6.19	610.5465	2.12E-05	3.92E-05	10.4
DAG 34:3e (DAG 18:3e/16:0)	$[M+NH_4]^+$	6.74	594.5430	6.78E-14	2.53E-12	393.5
DAG 35:0 (DAG 17:0-18:0)	$[M+NH_4]^+$	7.69	628.5878	4.87E-07	1.12E-06	7.06
DAG 35:1 (DAG 17:0-18:1)	$[M+NH_4]^+$	7.08	626.5710	6.52E-09	2.05E-08	14.5
DAG 35:1e (DAG 17:0e/18:1)	$[M+NH_4]^+$	7.20	612.5916	8.26E-06	1.80E-05	12.0
DAG 35:2 (DAG 17:1-18:1)	$[M+NH_4]^+$	6.48	624.5538	2.01E-12	2.73E-11	37.1
DAG 35:3e (DAG 17:2e/18:1)	$[M+NH_4]^+$	7.06	608.5609	4.40E-14	1.48E-12	151.0
DAG 36:1 (DAG 16:0-20:1)	$[M+NH_4]^+$	7.41	640.5872	4.68E-05	8.92E-05	18.1
DAG 36:2 (DAG 18:1-18:1)	$[M+NH_4]^+$	6.81	638.5745	4.12E-03	8.28E-03	6.83
DAG 37:1 (DAG 17:0-20:1)	$[M+NH_4]^+$	7.71	654.6033	1.92E-06	4.30E-06	12.9
DAG 38:0 (DAG 18:0-20:0)	$[M+NH_4]^+$	8.63	670.6351	2.91E-04	5.72E-04	5.38
DAG 38:1 (DAG 18:0-20:1)	$[M+NH_4]^+$	8.04	668.6241	1.96E-06	4.44E-06	82.4
DAG 38:2 (DAG 18:1-20:1)	$[M+NH_4]^+$	7.43	666.5981	8.66E-07	1.93E-06	26.7
DAG 40:2 (DAG 20:1-20:1)	$[M+NH_4]^+$	8.04	694.6325	1.02E-06	2.28E-06	42.1
DGDG 28:0 (DGDG 12:0-16:0)	$[M+NH_4]^+$	4.11	854.5868	8.16E-10	3.28E-09	30.8
DGDG 29:0 (DGDG 13:0-16:0)	$[M+NH_4]^+$	4.39	868.6011	6.13E-10	2.33E-09	36.8
DGDG 30:0 (DGDG 14:0-16:0)	$[M+NH_4]^+$	4.67	882.6160	9.39E-12	8.44E-11	44.7
DGDG 30:1 (DGDG 16:0-14:1)	$[M+NH_4]^+$	4.22	880.5978	1.05E-09	3.83E-09	43.2
DGDG 31:0 (DGDG 15:0-16:0)	$[M+NH_4]^+$	4.98	896.6327	9.12E-12	8.13E-11	65.4
DGDG 31:1 (DGDG 15:0-16:1)	$[M+NH_4]^+$	4.47	894.6148	1.27E-10	5.46E-10	26.3
DGDG 32:0 (DGDG 16:0-16:0)	$[M+NH_4]^+$	5.30	910.6454	5.98E-14	2.00E-12	135.9
DGDG 32:1 (DGDG 16:0-16:1)	$[M+NH_4]^+$	4.73	908.6305	2.34E-07	6.70E-07	23.3
DGDG 32:2 (DGDG 14:0-18:2)	$[M+NH_4]^+$	4.30	906.6155	3.54E-05	6.85E-05	25.0
DGDG 33:0 (DGDG 16:0-17:0)	$[M+NH_4]^+$	5.62	924.6664	1.00E-09	3.71E-09	41.5
DGDG 34:0 (DGDG 16:0-18:0)	$[M+NH_4]^+$	5.95	938.6730	7.63E-10	2.85E-09	104.5
DGDG 34:1 (DGDG 16:0-18:1)	$[M+NH_4]^+$	5.35	936.6611	2.89E-14	7.68E-13	135.1
DGDG 34:2 (DGDG 16:0-18:2)	$[M+NH_4]^+$	4.88	934.6502	2.69E-06	5.94E-06	35.5
DGDG 35:1 (DGDG 17:0-18:1)	$[M+NH_4]^+$	5.67	950.6761	3.28E-09	1.17E-08	57.1
DGDG 35:2 (DGDG 17:1-18:1)	$[M+NH_4]^+$	5.12	948.6644	2.62E-04	5.07E-04	11.8
DGDG 36:1 (DGDG 16:0-20:1)	$[M+NH_4]^+$	5.98	964.7014	2.21E-09	7.94E-09	151.2

DGDG 38:1 (DGDG 18:0-20:1)	$[M+NH_4]^+$	6.64	992.7289	6.95E-06	1.50E-05	36.8
HBMP 48:1 (HBMP 16:0/14:0-18:1)	$[M+NH_4]^+$	7.60	976.7543	1.17E-08	3.51E-08	8.67
HexCer-NDS d46:0 (HexCer-NDS d20:0/26:0)	$[M+H]^+$	8.91	870.7714	6.80E-03	1.48E-02	2.76
LPC 28:1	$[M+H]^+$	4.61	662.5189	3.89E-09	1.41E-08	34.2
LPE 13:0	$[M+H]^+$	0.76	412.2460	2.80E-05	5.20E-05	44.5
LPE 14:0	$[M+H]^+$	0.89	426.2610	1.23E-06	2.79E-06	22.5
LPE 15:1e	$[M+H]^+$	1.31	424.2815	4.47E-11	2.75E-10	41.5
LPE 16:0	$[M+H]^+$	1.42	454.2922	1.25E-04	2.48E-04	8.64
LPE 16:1	$[M+H]^+$	1.00	452.2746	4.19E-04	8.23E-04	11.7
LPE 16:1e	$[M+H]^+$	1.63	438.2986	1.51E-03	3.01E-03	3.20
LPE 16:2e	$[M+H]^+$	1.21	436.2815	1.54E-08	4.53E-08	51.4
LPE 17:0	$[M+H]^+$	1.67	468.3086	3.03E-09	1.13E-08	17.5
LPE 17:1	$[M+H]^+$	1.19	466.2939	2.66E-11	1.81E-10	113.7
LPE 17:1e	$[M+H]^+$	2.00	452.3100	3.41E-06	7.42E-06	8.03
LPE 18:1	$[M+H]^+$	1.45	480.3073	8.41E-06	1.84E-05	4.35
MGDG 28:0 (MGDG 12:0-16:0)	$[M+NH_4]^+$	4.52	692.5338	3.69E-14	9.54E-13	50.9
MGDG 30:0 (MGDG 14:0-16:0)	$[M+NH_4]^+$	5.14	720.5605	5.83E-14	1.70E-12	87.1
MGDG 30:1 (MGDG 14:0-16:1)	$[M+NH_4]^+$	4.67	718.5435	3.38E-09	1.22E-08	20.2
MGDG 31:0 (MGDG 15:0-16:0)	$[M+NH_4]^+$	5.45	734.5798	8.81E-06	1.91E-05	11.6
MGDG 32:0 (MGDG 16:0-16:0)	$[M+NH_4]^+$	5.79	748.5918	3.81E-09	1.34E-08	37.9
MGDG 32:1 (MGDG 16:0-16:1)	$[M+NH_4]^+$	5.25	746.5783	6.67E-12	6.65E-11	37.6
MGDG 33:0 (MGDG 16:0-17:0)	$[M+NH_4]^+$	6.10	762.6077	3.75E-08	1.24E-07	17.0
MGDG 33:1 (MGDG 15:0-18:1)	$[M+NH_4]^+$	5.52	760.5908	8.60E-05	1.53E-04	3.51
MGDG 34:0 (MGDG 16:0-18:0)	$[M+NH_4]^+$	6.46	776.6257	1.90E-08	5.82E-08	20.1
MGDG 34:1 (MGDG 16:0-18:1)	$[M+NH_4]^+$	5.84	774.6078	2.35E-08	7.04E-08	32.8
MGDG 34:2 (MGDG 16:1-18:1)	$[M+NH_4]^+$	5.32	772.5945	5.37E-09	1.77E-08	11.8
MGDG 35:1 (MGDG 17:0-18:1)	$[M+NH_4]^+$	6.17	788.6209	6.19E-05	1.14E-04	4.24
MGDG 36:0 (MGDG 16:0-20:0)	$[M+NH_4]^+$	7.12	804.6601	7.61E-06	1.71E-05	9.12
MGDG 36:1 (MGDG 18:0-18:1)	$[M+NH_4]^+$	6.48	802.6369	5.56E-09	1.84E-08	60.1
MGDG 36:2 (MGDG 18:1-18:1)	$[M+NH_4]^+$	5.91	800.6268	2.85E-10	1.16E-09	16.4
MGDG 38:1 (MGDG 18:0-20:1)	$[M+NH_4]^+$	7.13	830.6688	6.00E-07	1.43E-06	28.9
MGDG 38:2 (MGDG 18:1-20:1)	$[M+NH_4]^+$	6.51	828.6534	2.14E-07	6.28E-07	12.4
PC 34:6e	$[M+H]^+$	4.68	736.5444	5.34E-07	1.21E-06	21.8
PE 23:0	$[M+H]^+$	3.04	566.3818	2.26E-08	6.99E-08	24.4

PE 24:0 (PE 12:0-12:0)	$[M+H]^+$	3.23	580.3985	3.00E-10	1.24E-09	69.5
PE 25:0	$[M+H]^+$	3.45	594.4138	1.21E-12	1.71E-11	161.4
PE 26:0 (PE 13:0-13:0)	$[M+H]^+$	3.68	608.4328	8.42E-10	3.30E-09	280.8
PE 26:1	$[M+H]^+$	3.33	606.4132	6.35E-07	1.55E-06	14.5
PE 26:1e (PE 15:1e/11:0)	$[M+H]^+$	3.92	592.4348	6.51E-12	6.55E-11	221.2
PE 27:0 (PE 13:0-14:0)	$[M+H]^+$	3.93	622.4463	4.53E-08	1.65E-07	317.2
PE 27:1	$[M+H]^+$	3.57	620.4280	1.43E-10	6.58E-10	22.0
PE 27:1e (PE 15:1e/12:0)	$[M+H]^+$	4.19	606.4514	6.68E-15	4.21E-13	239.9
PE 28:0 (PE 14:0-14:0)	$[M+H]^+$	4.21	636.4627	1.27E-08	3.78E-08	103.4
PE 28:1 (PE 12:0-16:1)	$[M+H]^+$	3.78	634.4467	3.91E-13	7.34E-12	67.6
PE 28:1e (PE 15:1e/13:0)	$[M+H]^+$	4.49	620.4638	3.32E-11	2.18E-10	237.0
PE 28:2e (PE 16:2e/12:0)	$[M+H]^+$	4.03	618.4517	1.68E-09	6.48E-09	285.9
PE 29:0 (PE 13:0-16:0)	$[M+H]^+$	4.49	650.4805	5.48E-06	1.16E-05	127.2
PE 29:1 (PE 13:0-16:1)	$[M+H]^+$	4.04	648.4604	6.76E-09	2.18E-08	124.1
PE 29:1e (PE 16:1e/13:0)	$[M+H]^+$	4.80	634.4822	1.12E-08	3.39E-08	250.8
PE 29:2e (PE 16:2e/13:0)	$[M+H]^+$	4.31	632.4659	1.37E-10	6.13E-10	231.7
PE 30:0 (PE 14:0-16:0)	$[M+H]^+$	4.80	664.4946	6.97E-06	1.51E-05	40.1
PE 30:1 (PE 14:0-16:1)	$[M+H]^+$	4.31	662.4819	1.20E-06	2.75E-06	58.3
PE 30:1e (PE 16:1e/14:0)	$[M+H]^+$	5.12	648.4958	2.75E-09	9.76E-09	191.1
PE 30:2	$[M+H]^+$	3.95	660.4614	4.82E-09	1.65E-08	63.2
PE 30:2e (PE 16:2e/14:0)	$[M+H]^+$	4.62	646.4829	1.60E-10	7.10E-10	297.2
PE 30:3e (PE 16:2e/14:1)	$[M+H]^+$	4.22	644.4726	1.70E-12	2.55E-11	53.4
PE 31:1 (PE 15:0-16:1)	$[M+H]^+$	4.60	676.4819	4.55E-07	1.11E-06	177.7
PE 31:1e (PE 16:1e/15:0)	$[M+H]^+$	5.35	662.5106	2.98E-10	1.23E-09	182.6
PE 31:2	$[M+H]^+$	4.20	674.4795	8.44E-11	3.86E-10	80.2
PE 31:2e (PE 15:1e/16:1)	$[M+H]^+$	4.91	660.4976	2.65E-16	1.38E-13	332.9
PE 31:3e (PE 16:2e/15:1)	$[M+H]^+$	4.45	658.4838	4.76E-10	2.00E-09	50.6
PE 32:0 (PE 16:0-16:0)	$[M+H]^+$	5.45	692.5227	5.18E-07	1.18E-06	47.6
PE 32:1 (PE 16:0-16:1)	$[M+H]^+$	4.89	690.5115	2.58E-06	5.67E-06	34.5
PE 32:1e (PE 16:1e/16:0)	$[M+H]^+$	5.79	676.5284	7.80E-13	1.24E-11	113.9
PE 32:2 (PE 16:1-16:1)	$[M+H]^+$	4.44	688.4930	6.00E-06	1.31E-05	143.2
PE 32:2e (PE 15:1e/17:1)	$[M+H]^+$	5.20	674.5161	2.54E-15	2.04E-13	336.9
PE 32:3e (PE 16:2e/16:1)	$[M+H]^+$	4.72	672.4976	1.48E-12	2.02E-11	291.7
PE 33:0	$[M+H]^+$	5.78	706.5369	1.14E-07	3.92E-07	52.9
PE 33:1 (PE 15:0-18:1)	$[M+H]^+$	5.20	704.5251	2.75E-09	9.97E-09	150.8

PE 33:1e (PE 17:1e/16:0)	$[M+H]^+$	6.12	690.5411	6.67E-11	3.44E-10	56.8
PE 33:2 (PE 16:1-17:1)	$[M+H]^+$	4.69	702.5046	2.60E-08	8.41E-08	82.4
PE 33:2e (PE 16:1e/17:1)	$[M+H]^+$	5.52	688.5303	3.56E-15	2.80E-13	183.1
PE 33:3e (PE 16:2e/17:1)	$[M+H]^+$	5.00	686.5108	7.40E-11	3.69E-10	535.7
PE 34:0	$[M+H]^+$	6.12	720.5485	5.72E-07	1.32E-06	45.8
PE 34:1 (PE 16:0-18:1)	$[M+H]^+$	5.52	718.5433	2.15E-08	6.33E-08	54.5
PE 34:1e (PE 16:1e/18:0)	$[M+H]^+$	6.46	704.5559	6.63E-12	6.59E-11	15.9
PE 34:2 (PE 17:1-17:1)	$[M+H]^+$	5.00	716.5295	8.95E-09	2.60E-08	63.0
PE 34:2e (PE 16:1e/18:1)	$[M+H]^+$	5.85	702.5455	1.45E-10	6.58E-10	26.9
PE 34:3	$[M+H]^+$	4.65	714.5111	5.11E-09	1.70E-08	18.3
PE 34:3e (PE 16:2e/18:1)	$[M+H]^+$	5.31	700.5325	3.11E-12	3.37E-11	152.3
PE 35:1 (PE 17:0-18:1)	$[M+H]^+$	5.84	732.5546	5.27E-12	4.97E-11	54.4
PE 35:2 (PE 17:1-18:1)	$[M+H]^+$	5.28	730.5375	4.17E-14	1.46E-12	117.1
PE 35:2e (PE 17:1e/18:1)	$[M+H]^+$	6.19	716.5585	1.67E-11	1.36E-10	32.9
PE 35:3e (PE 17:2e/18:1)	$[M+H]^+$	5.59	714.5439	2.31E-13	5.38E-12	198.9
PE 36:0	$[M+H]^+$	6.80	748.5908	2.37E-06	5.40E-06	9.39
PE 36:1 (PE 18:0-18:1)	$[M+H]^+$	6.18	746.5701	6.91E-04	1.28E-03	3.68
PE 36:2 (PE 18:1-18:1)	$[M+H]^+$	5.58	744.5559	6.40E-08	2.37E-07	29.3
PE 36:3e (PE 18:2e/18:1)	$[M+H]^+$	5.93	728.5577	9.22E-09	2.60E-08	20.4
PE 37:2	$[M+H]^+$	5.89	758.5725	7.98E-10	3.03E-09	21.0
PE 38:1	$[M+H]^+$	6.82	774.6108	4.15E-04	8.16E-04	5.64
PG 28:0 (PG 14:0-14:0)	$[M+NH_4]^+$	3.65	684.4782	3.99E-06	8.38E-06	21.0
PG 30:0 (PG 14:0-16:0)	$[M+NH_4]^+$	4.13	712.5117	1.81E-06	3.97E-06	17.8
PG 30:1	$[M+NH_4]^+$	3.73	710.4949	2.01E-05	3.66E-05	12.1
PG 31:0 (PG 15:0-16:0)	$[M+NH_4]^+$	4.33	726.5245	7.23E-09	2.36E-08	27.1
PG 32:0 (PG 16:0-16:0)	$[M+NH_4]^+$	4.67	740.5414	2.37E-05	4.11E-05	5.28
PG 32:1 (PG 16:0-16:1)	$[M+NH_4]^+$	4.20	738.5335	3.73E-05	7.19E-05	9.99
PG 32:2 (PG 16:1-16:1)	$[M+NH_4]^+$	3.80	736.5156	1.53E-05	2.83E-05	21.1
PG 33:1 (PG 16:0-17:1)	$[M+NH_4]^+$	4.40	752.5438	3.61E-08	1.20E-07	58.1
PG 33:2	$[M+NH_4]^+$	4.05	750.5264	3.16E-08	1.06E-07	18.3
PG 34:1 (PG 16:0-18:1)	$[M+NH_4]^+$	4.71	766.5612	4.93E-04	9.51E-04	5.27
PG 34:2 (PG 16:0-18:2)	$[M+NH_4]^+$	4.26	764.5390	2.36E-06	5.40E-06	14.2
PG 35:1 (PG 18:0-17:1)	$[M+NH_4]^+$	4.95	780.5741	5.31E-06	1.10E-05	27.7
PG 35:2 (PG 17:1-18:1)	$[M+NH_4]^+$	4.53	778.5579	5.62E-10	2.28E-09	30.2
PG 36:1 (PG 18:0-18:1)	$[M+NH_4]^+$	5.30	794.5896	1.78E-06	3.86E-06	10.3

PG 36:2 (PG 18:1-18:1)	$[M+NH_4]^+$	4.77	792.5756	1.82E-04	3.66E-04	7.26
SM d36:0	$[M+H]^+$	5.45	733.6223	1.03E-02	3.26E-02	3.34

Only identified lipids that show an SGoF adjusted *p* value (Mann-Whitney *U* test) below the significance level of $\alpha = 0.05$ are listed. Foldchanges are calculated based on median intensity values between experimental groups. Information about side chain composition is given in parentheses, if available. Here, a foldchange of >2 means more than factor 2 higher in BF samples

Table S9 Comparison of *in vitro* biofilm versus *in vivo* plaque samples (BF vs. PL, foldchange <0.5, negative mode)

**Identified lipid**	**Adduct type**	**t_R [min]**	**Measured *m/z***	***p* value**	**SGoF *p* value**	**Fold-change**
Cholesterol-sulfate	$[M-H]^-$	2.96	465.3035	3.80E-05	8.93E-05	0.11
PC 32:0 (PC 16:0-16:0)	$[M+HCOO]^-$	5.30	778.5602	7.77E-06	1.99E-05	0.13
PC 32:0e (PC 16:0e/16:0)	$[M+HCOO]^-$	5.70	764.5800	3.14E-03	1.11E-02	0.30
PC 34:1 (PC 16:0-18:1)	$[M+HCOO]^-$	5.38	804.5743	2.04E-04	5.58E-04	0.16
PC 34:1e (PC 16:0e/18:1)	$[M+HCOO]^-$	5.76	790.5953	6.61E-03	2.76E-02	0.35
PC 36:1 (PC 18:0-18:1)	$[M+HCOO]^-$	6.03	832.6066	9.31E-04	2.03E-03	0.24
PC 36:2 (PC 18:1-18:1)	$[M+HCOO]^-$	5.47	830.5930	9.80E-04	2.12E-03	0.25
PE 36:5e (PE 16:1e/20:4)	$[M-H]^-$	5.24	722.5116	1.62E-03	3.14E-03	0.17

Only identified lipids that show an SGoF adjusted *p* value (Mann-Whitney *U* test) below the significance level of $\alpha = 0.05$ are listed. Foldchanges are calculated based on median intensity values between experimental groups. Information about side chain composition is given in parentheses, if available. Here, a foldchange of <0.5 means more than factor 2 higher in PL samples

Table S10 Comparison of *in vitro* biofilm versus *in vivo* plaque samples (BF vs. PL, foldchange >2, negative mode)

Identified lipid	Adduct type	t_R[min]	Measured m/z	*p* value	SGoF *p* value	Foldchange
Cer-AP t34:0+O (Cer-AP t18:0/16:0+O)	$[M+HCOO]^-$	4.84	616.5122	9.95E-04	2.12E-03	6.52
Cer-BDS d33:0+O (Cer-BDS d16:0/17:0+O)	$[M+HCOO]^-$	4.63	586.5012	3.72E-07	9.67E-07	235.5
Cer-BDS d34:0+O (Cer-BDS d17:0/17:0+O)	$[M+HCOO]^-$	4.97	600.5175	2.67E-06	6.61E-06	177.2
Cer-BDS d35:0+O (Cer-BDS d18:0/17:0+O)	$[M+HCOO]^-$	5.27	614.5360	4.42E-07	1.08E-06	97.6
Cer-BDS d36:0+O (Cer-BDS d19:0/17:0+O)	$[M+HCOO]^-$	5.52	628.5465	1.65E-06	4.12E-06	124.3
Cer-BS d36:1+O (Cer-BS d18:1/18:0+O)	$[M-H]^-$	5.42	580.5223	1.79E-09	9.02E-09	511.5
DGDG 28:0 (DGDG 12:0-16:0)	$[M+HCOO]^-$	4.14	881.5456	1.04E-05	2.77E-05	65.3
DGDG 29:0	$[M+HCOO]^-$	4.40	895.5602	1.28E-09	6.02E-09	165.0
DGDG 30:0 (DGDG 14:0-16:0)	$[M+HCOO]^-$	4.70	909.5790	2.38E-07	6.38E-07	154.4
DGDG 30:1	$[M+HCOO]^-$	4.25	907.5594	1.57E-06	3.83E-06	91.5
DGDG 31:0 (DGDG 15:0-16:0)	$[M+HCOO]^-$	5.00	923.5910	2.63E-09	1.17E-08	48.6
DGDG 32:0 (DGDG 16:0-16:0)	$[M+HCOO]^-$	5.33	937.6107	2.71E-05	5.69E-05	73.9
DGDG 32:1 (DGDG 16:0-16:1)	$[M+HCOO]^-$	4.79	935.5946	3.06E-07	8.59E-07	89.6
DGDG 33:1	$[M+HCOO]^-$	5.08	949.6091	1.51E-05	3.73E-05	77.4
DGDG 34:0 (DGDG 16:0-18:0)	$[M+HCOO]^-$	5.99	965.6425	2.41E-06	5.91E-06	103.5
DGDG 34:1 (DGDG 16:0-18:1)	$[M+HCOO]^-$	5.38	963.6271	3.14E-12	2.81E-11	57.6
DGDG 34:2 (DGDG 16:1-18:1)	$[M+HCOO]^-$	4.90	961.6096	2.61E-05	5.47E-05	22.8
DGDG 36:1 (DGDG 18:0-18:1)	$[M+HCOO]^-$	6.03	991.6603	4.58E-10	2.25E-09	133.9
DGDG 36:2 (DGDG 18:1-18:1)	$[M+HCOO]^-$	5.45	989.6401	5.31E-05	1.39E-04	22.5
DGDG 38:1 (DGDG 18:0-20:1)	$[M+HCOO]^-$	6.63	1019.6854	2.61E-06	6.40E-06	42.6
DGDG 38:2 (DGDG 18:1-20:1)	$[M+HCOO]^-$	6.06	1017.6682	4.90E-08	1.75E-07	84.5
FA 20:0	$[M-H]^-$	4.19	311.2922	2.88E-07	8.41E-07	110.8
FA 20:1	$[M-H]^-$	3.64	309.2770	9.52E-13	1.51E-11	225.9
FA 22:0	$[M-H]^-$	4.88	339.3256	9.63E-05	2.23E-04	6.10
FA 22:1	$[M-H]^-$	4.22	337.3081	1.52E-06	3.52E-06	49.3
FA 24:0	$[M-H]^-$	5.63	367.3553	4.33E-05	1.14E-04	5.91
FA 26:0	$[M-H]^-$	6.40	395.3857	7.48E-04	1.78E-03	5.49
HBMP 54:2 (HBMP 18:0-18:1-18:1)	$[M-H]^-$	9.56	1039.8059	3.69E-05	8.78E-05	18.6
LPE 12:0	$[M-H]^-$	0.67	396.2105	1.37E-10	7.02E-10	34.4
LPE 13:0	$[M-H]^-$	0.77	410.2293	1.31E-09	6.54E-09	96.4
LPE 14:0	$[M-H]^-$	0.91	424.2460	9.49E-07	2.19E-06	50.3

LPE 14:1e	$[M-H]^-$	1.09	408.2481	3.42E-06	7.89E-06	10.0
LPE 15:0	$[M-H]^-$	1.13	438.2623	7.19E-04	1.70E-03	32.0
LPE 15:1	$[M-H]^-$	0.86	436.2426	3.92E-10	1.80E-09	79.1
LPE 15:1e	$[M-H]^-$	1.32	422.2666	1.81E-07	4.99E-07	177.8
LPE 16:0	$[M-H]^-$	1.44	452.2759	1.24E-04	3.15E-04	12.8
LPE 16:1	$[M-H]^-$	1.02	450.2593	5.58E-04	1.39E-03	17.0
LPE 16:1e	$[M-H]^-$	1.66	436.2774	1.54E-03	2.92E-03	3.44
LPE 16:2e	$[M-H]^-$	1.23	434.2644	8.73E-10	4.28E-09	216.9
LPE 17:0	$[M-H]^-$	1.71	466.2907	7.35E-08	2.43E-07	152.6
LPE 17:1	$[M-H]^-$	1.21	464.2730	4.15E-12	3.35E-11	354.7
LPE 17:1e	$[M-H]^-$	2.03	450.2922	9.12E-06	2.57E-05	11.3
LPE 17:2e	$[M-H]^-$	1.49	448.2799	1.36E-09	7.56E-09	81.3
LPE 18:1	$[M-H]^-$	1.58	478.2883	4.55E-04	1.04E-03	12.9
MGDG 26:0 (MGDG 12:0-14:0)	$[M+HCOO]^-$	4.05	691.4645	1.05E-07	2.96E-07	182.4
MGDG 28:0 (MGDG 12:0-16:0)	$[M+HCOO]^-$	4.61	719.4913	3.50E-08	1.21E-07	190.7
MGDG 28:1e (MGDG 16:1e/12:0)	$[M+HCOO]^-$	4.94	703.4975	3.73E-05	8.81E-05	180.4
MGDG 29:1e (MGDG 15:1e/14:0)	$[M+HCOO]^-$	5.18	717.5109	6.89E-10	3.62E-09	714.0
MGDG 29:2e (MGDG 16:2e/13:0)	$[M+HCOO]^-$	4.72	715.4974	1.89E-11	1.48E-10	320.3
MGDG 30:0 (MGDG 14:0-16:0)	$[M+HCOO]^-$	5.16	747.5242	3.18E-09	1.40E-08	84.4
MGDG 30:1e (MGDG 16:1e/14:0)	$[M+HCOO]^-$	5.60	731.5291	2.27E-06	5.69E-06	431.9
MGDG 31:0 (MGDG 15:0-16:0)	$[M+HCOO]^-$	5.47	761.5392	2.22E-04	5.88E-04	154.2
MGDG 31:1e (MGDG 15:1e/16:0)	$[M+HCOO]^-$	5.84	745.5432	7.41E-09	2.64E-08	365.9
MGDG 31:2e (MGDG 17:2e/14:0)	$[M+HCOO]^-$	5.33	743.5311	5.27E-11	3.48E-10	551.8
MGDG 32:0 (MGDG 16:0-16:0)	$[M+HCOO]^-$	5.82	775.5586	2.15E-08	6.37E-08	70.0
MGDG 32:1 (MGDG 14:0-18:1)	$[M+HCOO]^-$	5.29	773.5416	6.45E-07	1.64E-06	55.9
MGDG 32:1e (MGDG 16:1e/16:0)	$[M+HCOO]^-$	6.26	759.5630	1.45E-07	4.18E-07	1003
MGDG 32:2e (MGDG 18:2e/14:0)	$[M+HCOO]^-$	5.66	757.5476	4.28E-09	1.71E-08	988.2
MGDG 33:0 (MGDG 16:0-17:0)	$[M+HCOO]^-$	6.15	789.5720	9.41E-07	2.17E-06	199.4
MGDG 33:1 (MGDG 15:0-18:1)	$[M+HCOO]^-$	5.60	787.5548	2.76E-06	6.89E-06	101.6
MGDG 33:2e (MGDG 18:2e/15:0)	$[M+HCOO]^-$	6.00	771.5538	3.33E-10	1.58E-09	952.8
MGDG 34:0 (MGDG 16:0-18:0)	$[M+HCOO]^-$	6.49	803.5891	8.33E-08	2.67E-07	135.6
MGDG 34:1 (MGDG 16:0-18:1)	$[M+HCOO]^-$	5.88	801.5709	5.45E-06	1.42E-05	17.2
MGDG 34:2 (MGDG 16:1-18:1)	$[M+HCOO]^-$	5.38	799.5554	2.69E-04	6.95E-04	16.6
MGDG 34:2e (MGDG 18:2e/16:0)	$[M+HCOO]^-$	6.32	785.5767	3.99E-07	9.99E-07	1107
MGDG 35:1 (MGDG 17:0-18:1)	$[M+HCOO]^-$	6.23	815.5907	2.18E-07	5.93E-07	138.8

MGDG 35:2 (MGDG 17:1-18:1)	$[M+HCOO]^-$	5.65	813.5728	1.72E-07	4.72E-07	84.0
MGDG 35:2e (MGDG 19:2e/16:0)	$[M+HCOO]^-$	6.68	799.5913	1.10E-05	2.83E-05	90.3
MGDG 36:1 (MGDG 16:0-20:1)	$[M+HCOO]^-$	6.52	829.6043	3.19E-08	1.11E-07	65.3
MGDG 36:2 (MGDG 18:1-18:1)	$[M+HCOO]^-$	5.96	827.5902	2.38E-06	5.71E-06	25.1
MGDG 36:3e (MGDG 18:2e/18:1)	$[M+HCOO]^-$	6.38	811.5878	2.27E-08	7.12E-08	155.3
MGDG 38:1 (MGDG 18:0-20:1)	$[M+HCOO]^-$	7.17	857.6360	2.24E-06	5.68E-06	612.3
PC 34:2e	$[M+HCOO]^-$	5.66	788.5779	4.82E-05	1.27E-04	4.38
PE 24:0 (PE 10:0-14:0)	$[M-H]^-$	3.25	578.3771	8.42E-13	1.38E-11	578.9
PE 25:0 (PE 12:0-13:0)	$[M-H]^-$	3.46	592.3937	5.68E-06	1.48E-05	774.5
PE 26:0 (PE 12:0-14:0)	$[M-H]^-$	3.70	606.4115	4.00E-08	1.44E-07	500.3
PE 26:1 (PE 11:0-15:1)	$[M-H]^-$	3.35	604.3950	5.42E-15	1.26E-12	324.0
PE 26:1e (PE 13:1e/13:0)	$[M-H]^-$	3.94	590.4179	3.37E-08	1.13E-07	681.8
PE 27:1e (PE 15:1e/12:0)	$[M-H]^-$	4.21	604.4296	6.10E-08	2.09E-07	828.9
PE 28:0 (PE 14:0-14:0)	$[M-H]^-$	4.23	634.4411	3.26E-07	9.11E-07	89.0
PE 28:1 (PE 12:0-16:1)	$[M-H]^-$	3.81	632.4281	1.06E-06	2.54E-06	503.2
PE 28:1e (PE 15:1e/13:0)	$[M-H]^-$	4.51	618.4460	6.36E-11	4.21E-10	180.3
PE 28:2e (PE 16:2e/12:0)	$[M-H]^-$	4.05	616.4324	6.46E-12	4.96E-11	1642
PE 29:0 (PE 13:0-16:0)	$[M-H]^-$	4.51	648.4599	5.88E-04	1.49E-03	53.1
PE 29:1 (PE 13:0-16:1)	$[M-H]^-$	4.06	646.4427	7.34E-07	1.75E-06	798.0
PE 29:1e (PE 16:1e/13:0)	$[M-H]^-$	4.82	632.4639	4.51E-06	1.17E-05	262.1
PE 29:2e (PE 16:2e/13:0)	$[M-H]^-$	4.33	630.4467	1.65E-08	5.34E-08	1664
PE 30:1 (PE 14:0-16:1)	$[M-H]^-$	4.32	660.4586	5.06E-05	1.33E-04	66.0
PE 30:1e (PE 16:1e/14:0)	$[M-H]^-$	5.15	646.4804	2.08E-07	5.58E-07	145.7
PE 30:2 (PE 13:1-17:1)	$[M-H]^-$	3.98	658.4445	8.29E-15	1.79E-12	137.3
PE 30:2e (PE 16:2e/14:0)	$[M-H]^-$	4.62	644.4628	8.10E-07	1.86E-06	548.9
PE 31:0 (PE 15:0-16:0)	$[M-H]^-$	5.06	676.4894	2.75E-04	7.00E-04	12.3
PE 31:1 (PE 15:0-16:1)	$[M-H]^-$	4.60	674.4746	1.27E-04	3.23E-04	127.7
PE 31:1e (PE 16:1e/15:0)	$[M-H]^-$	5.41	660.4916	1.40E-08	4.28E-08	193.7
PE 31:2 (PE 14:1-17:1)	$[M-H]^-$	4.22	672.4576	4.55E-10	2.23E-09	306.7
PE 31:2e (PE 15:1e/16:1)	$[M-H]^-$	4.94	658.4771	3.13E-08	1.08E-07	408.4
PE 31:3e (PE 16:2e/15:1)	$[M-H]^-$	4.47	656.4625	3.60E-10	1.68E-09	500.9
PE 32:0 (PE 16:0-16:0)	$[M-H]^-$	5.47	690.5032	8.37E-05	1.99E-04	34.5
PE 32:1 (PE 15:0-17:1)	$[M-H]^-$	4.91	688.4897	1.87E-05	4.33E-05	21.8
PE 32:1e (PE 16:1e/16:0)	$[M-H]^-$	5.81	674.5096	4.32E-07	1.07E-06	101.0
PE 32:2 (PE 16:1-16:1)	$[M-H]^-$	4.46	686.4739	4.76E-05	1.23E-04	236.8

PE 32:2e (PE 15:1e/17:1)	$[M-H]^-$	5.22	672.4962	3.62E-09	1.45E-08	138.0
PE 32:3e (PE 16:2e/16:1)	$[M-H]^-$	4.77	670.4800	3.02E-08	1.04E-07	1968
PE 33:0 (PE 16:0-17:0)	$[M-H]^-$	5.73	704.5220	2.18E-05	4.82E-05	34.9
PE 33:1 (PE 16:0-17:1)	$[M-H]^-$	5.24	702.5054	4.61E-06	1.20E-05	85.5
PE 33:1e (PE 17:1e/16:0)	$[M-H]^-$	6.15	688.5234	1.61E-07	4.48E-07	206.2
PE 33:2 (PE 16:1-17:1)	$[M-H]^-$	4.74	700.4893	8.59E-07	1.96E-06	283.7
PE 33:2e (PE 16:1e/17:1)	$[M-H]^-$	5.55	686.5113	7.21E-13	1.06E-11	159.8
PE 33:3e (PE 16:2e/17:1)	$[M-H]^-$	5.03	684.4944	9.07E-08	2.80E-07	1243
PE 34:0 (PE 16:0-18:0)	$[M-H]^-$	6.14	718.5381	4.94E-05	1.30E-04	57.0
PE 34:1 (PE 16:0-18:1)	$[M-H]^-$	5.55	716.5217	6.16E-08	2.10E-07	27.3
PE 34:2 (PE 17:1-17:1)	$[M-H]^-$	5.02	714.5068	2.03E-08	6.21E-08	41.5
PE 34:2e (PE 16:1e/18:1)	$[M-H]^-$	5.88	700.5270	5.84E-08	2.04E-07	12.8
PE 34:3 (PE 16:1-18:2)	$[M-H]^-$	4.75	712.4835	3.04E-09	1.32E-08	58.7
PE 34:3e (PE 17:2e/17:1)	$[M-H]^-$	5.34	698.5120	2.81E-11	1.86E-10	75.2
PE 35:1 (PE 16:0-19:1)	$[M-H]^-$	5.94	730.5365	4.92E-06	1.26E-05	92.6
PE 35:2 (PE 17:1-18:1)	$[M-H]^-$	5.31	728.5223	1.76E-07	4.77E-07	146.2
PE 35:2e (PE 17:1e/18:1)	$[M-H]^-$	6.21	714.5412	1.80E-08	5.73E-08	38.0
PE 35:3e (PE 18:2e/17:1)	$[M-H]^-$	5.64	712.5286	1.76E-10	9.33E-10	174.6
PE 36:2 (PE 18:1-18:1)	$[M-H]^-$	5.62	742.5355	3.41E-07	9.32E-07	21.5
PE 36:3e (PE 18:2e/18:1)	$[M-H]^-$	5.96	726.5404	2.06E-08	6.32E-08	10.5
PE 37:2 (PE 18:1-19:1)	$[M-H]^-$	5.94	756.5530	2.87E-09	1.29E-08	125.5
PE 37:3e (PE 19:2e/18:1)	$[M-H]^-$	6.25	740.5615	2.67E-07	7.23E-07	72.3
PE-Cer t33:1 (PE-Cer t18:1/15:0)	$[M-H]^-$	3.93	661.4885	2.58E-08	8.33E-08	158.5
PE-Cer t34:0 (PE-Cer t18:0/16:0)	$[M-H]^-$	4.30	677.5208	1.07E-04	2.48E-04	39.3
PE-Cer t34:1 (PE-Cer t18:1/16:0)	$[M-H]^-$	4.19	675.5044	7.46E-07	1.77E-06	96.5
PE-Cer t35:0 (PE-Cer t16:0/19:0)	$[M-H]^-$	4.57	691.5377	8.62E-04	1.93E-03	11.6
PE-Cer t35:1 (PE-Cer t18:1/17:0)	$[M-H]^-$	4.43	689.5190	5.65E-05	1.44E-04	46.7
PE-Cer t36:1 (PE-Cer t20:1/16:0)	$[M-H]^-$	4.71	703.5391	2.13E-05	4.77E-05	70.4
PG 28:0 (PG 14:0-14:0)	$[M-H]^-$	3.69	665.4364	4.22E-05	1.04E-04	64.7
PG 29:0 (PG 14:0-15:0)	$[M-H]^-$	3.87	679.4535	2.54E-06	6.14E-06	181.7
PG 30:0 (PG 15:0-15:0)	$[M-H]^-$	4.10	693.4680	3.04E-06	7.77E-06	87.4
PG 31:0 (PG 15:0-16:0)	$[M-H]^-$	4.36	707.4868	3.54E-05	8.11E-05	95.8
PG 31:1 (PG 15:0-16:1)	$[M-H]^-$	3.96	705.4659	4.86E-06	1.25E-05	108.7
PG 32:0 (PG 16:0-16:0)	$[M-H]^-$	4.62	721.4943	2.15E-09	9.69E-09	60.0
PG 32:1 (PG 16:0-16:1)	$[M-H]^-$	4.24	719.4851	2.79E-05	5.98E-05	12.7

PG 32:2 (PG 16:1-16:1)	$[M-H]^-$	3.84	717.4684	1.04E-04	2.40E-04	37.6
PG 33:1 (PG 16:0-17:1)	$[M-H]^-$	4.46	733.4981	3.41E-07	9.26E-07	134.4
PG 33:2 (PG 16:1-17:1)	$[M-H]^-$	4.06	731.4824	3.99E-06	9.31E-06	72.1
PG 34:1 (PG 16:0-18:1)	$[M-H]^-$	4.79	747.5127	1.36E-05	3.35E-05	10.7
PG 34:2 (PG 16:1-18:1)	$[M-H]^-$	4.32	745.5009	4.32E-05	1.14E-04	16.1
PG 35:1 (PG 17:0-18:1)	$[M-H]^-$	5.07	761.5353	1.78E-06	4.32E-06	51.9
PG 35:2 (PG 17:1-18:1)	$[M-H]^-$	4.57	759.5125	1.62E-05	4.10E-05	99.9
PG 36:2 (PG 18:1-18:1)	$[M-H]^-$	4.84	773.5328	8.66E-05	2.01E-04	17.8
SHexCer d38:2	$[M-H]^-$	5.82	832.5696	2.54E-05	5.31E-05	79.2
SHexCer d38:3	$[M-H]^-$	5.29	830.5520	1.31E-08	4.00E-08	68.6

Only identified lipids that show an SGoF adjusted *p* value (Mann-Whitney *U* test) below the significance level of $\alpha = 0.05$ are listed. Foldchanges are calculated based on median intensity values between experimental groups. Information about side chain composition is given in parentheses, if available. Here, a foldchange of >2 means more than factor 2 higher in BF samples

Table S11 Comparison of formation times of *in vivo* plaque PL 24h vs. PL 72h (foldchange <0.5, positive mode)

**Identified lipid**	**Adduct type**	**t_R[min]**	**Measured m/z**	***p* value**	**SGoF *p* value**	**Foldchange**
LPC 18:1	$[M+H]^+$	1.40	522.3560	3.14E-02	4.81E-02	0.40
LPC 20:4	$[M+H]^+$	1.07	544.3384	1.11E-02	2.22E-02	0.49
LPE 17:1	$[M+H]^+$	1.19	466.2935	8.83E-03	1.77E-02	0.45
PE 30:0 (PE 14:0-16:0)	$[M+H]^+$	4.80	664.4874	3.24E-02	4.97E-02	0.36
PE 30:1 (PE 14:0-16:1)	$[M+H]^+$	4.30	662.4818	1.00E-02	1.95E-02	0.37
PE 30:1e (PE 16:1e/14:0)	$[M+H]^+$	5.12	648.4963	1.19E-02	2.38E-02	0.50
PE 32:0 (PE 16:0-16:0)	$[M+H]^+$	5.44	692.5181	1.99E-02	3.51E-02	0.38
PE 32:1 (PE 16:0-16:1)	$[M+H]^+$	4.89	690.5073	1.61E-02	3.00E-02	0.32
PE 33:3e	$[M+H]^+$	5.00	686.5116	7.12E-03	1.46E-02	0.46
PG 32:1 (PG 16:0-16:1)	$[M+NH_4]^+$	4.20	738.5284	6.70E-03	1.30E-02	0.35

Only identified lipids that show an SGoF adjusted *p* value (Wilcoxon signed-rank test) below the significance level of $\alpha = 0.05$ are listed. Foldchanges are calculated based on median intensity values between experimental groups. Information about side chain composition is given in parentheses, if available. Here, a foldchange of <0.5 means more than factor 2 higher in PL 72h samples

Table S12 Comparison of formation times of *in vivo* plaque PL 24h vs. PL 72h (foldchange >2, positive mode)

**Identified lipid**	**Adduct type**	**t_R[min]**	**Measured m/z**	***p* value**	**SGoF *p* value**	**Foldchange**
DAG 34:3 (DAG 16:1-18:2)	$[M+NH_4]^+$	5.63	608.5266	3.60E-04	2.78E-03	2.00
PC 36:2 (PC 18:1-18:1)	$[M+H]^+$	5.42	786.6022	1.50E-02	2.89E-02	2.88
PC 38:4	$[M+H]^+$	5.39	810.6030	2.31E-02	3.77E-02	2.64
PC 38:5	$[M+H]^+$	4.81	808.5938	9.63E-03	1.85E-02	2.41
TAG 52:0 (TAG 16:0-18:0-18:0)	$[M+NH_4]^+$	11.51	880.8319	1.86E-02	3.30E-02	3.96
TAG 52:1 (TAG 16:0-18:0-18:1)	$[M+Na]^+$	11.14	883.7772	3.39E-03	9.94E-03	4.73
TAG 52:2 (TAG 16:0-18:0-18:2)	$[M+Na]^+$	10.75	881.7565	1.25E-02	2.51E-02	2.06
TAG 54:1 (TAG 18:0-18:0-18:1)	$[M+Na]^+$	11.51	911.8041	1.05E-02	1.99E-02	14.9
TAG 54:2 (TAG 18:0-18:1-18:1)	$[M+Na]^+$	11.16	909.7966	1.34E-02	2.67E-02	3.17
TAG 56:0 (TAG 14:0-16:0-26:0)	$[M+NH_4]^+$	11.92	936.8981	1.51E-02	2.91E-02	2.13
TAG 57:1 (TAG 16:0-23:0-18:1)	$[M+NH_4]^+$	11.86	948.8994	7.12E-04	4.17E-03	2.28
TAG 60:1 (TAG 18:0-24:0-18:1)	$[M+NH_4]^+$	12.00	990.9432	2.83E-02	4.29E-02	2.96

Only identified lipids that show an SGoF adjusted *p* value (Wilcoxon signed-rank test) below the significance level of $\alpha = 0.05$ are listed. Foldchanges are calculated based on median intensity values between experimental groups. Information about side chain composition is given in parentheses, if available. Here, a foldchange of >2 means more than factor 2 higher in PL 24h samples

Table S13 Comparison of formation times of *in vivo* plaque samples PL 24h vs. PL 72h (foldchange <0.5, negative mode)

**Identified lipid**	**Adduct type**	**t_R[min]**	**Measured m/z**	***p* value**	**SGoF *p* value**	**Foldchange**
PE 31:2e (PE 15:1e/16:1)	$[M-H]^-$	4.94	658.4791	1.34E-03	3.80E-02	0.24
LPE 15:0	$[M-H]^-$	1.12	438.2596	7.67E-03	5.00E-02	0.15

Only identified lipids that show an SGoF adjusted *p* value (Wilcoxon signed-rank test) below the significance level of $\alpha = 0.05$ are listed. Foldchanges are calculated based on median intensity values between experimental groups. Information about side chain composition is given in parentheses, if available. No identified and significantly changed features with a foldchange >2 were detected in negative mode. Here, a foldchange of <0.5 means more than factor 2 higher in PL 72h samples

Table S14 Exemplary spectra (SWATH with high resolution MS/MS mode) of lipid class representatives in the real samples and comparison to reference spectra (note, some contaminating ions remained after deconvolution because of low concentration of lipids and/or low sensitivity in high-resolution mode and negative effect of high noise)

Cholesterylester (CE)
CE 19:2 $[M+NH_4]^+$

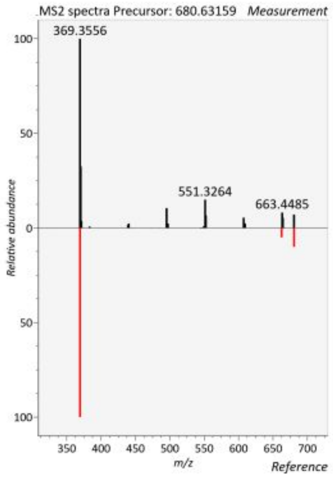

Ceramide alpha-hydroxy fatty acid-phytospingosine (Cer-AP)
Cer-AP t18:0/26:0+O $[M+H]^+$

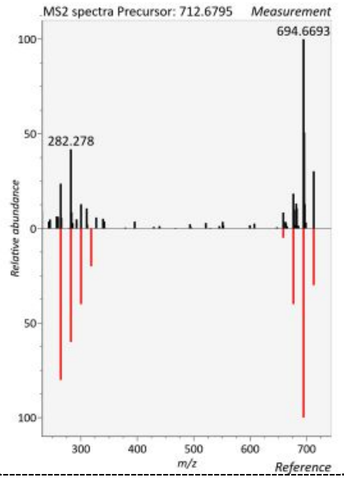

Ceramide beta-hydroxy fatty acid-dihydrosphingosine (Cer-BDS)
Cer-BDS d19:0/17:0+O $[M+HCOO]^-$

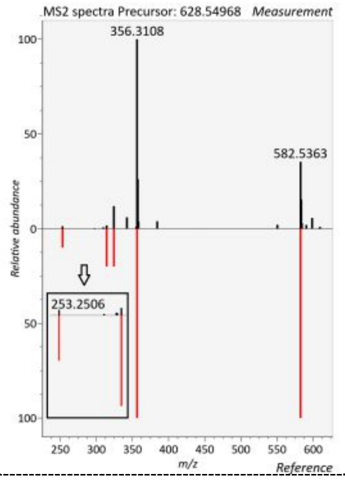

Ceramide non-hydroxy fatty acid-dihydrosphingosine (Cer-NDS)
Cer-NDS d18:0/16:0 $[M+H]^+$

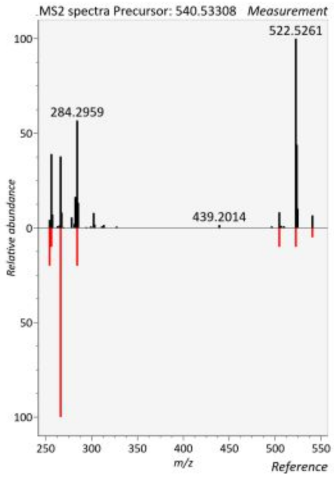

Ceramide non-hydroxy fatty acid-sphingosine (Cer-NS)
Cer-NS d18:1/16:0 $[M+H]^+$

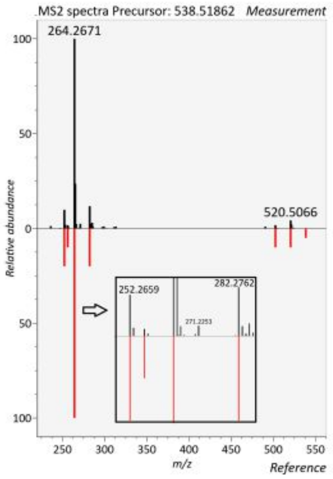

Diacylglycerol (DAG)
DAG 16:0-18:1 $[M+NH_4]^+$

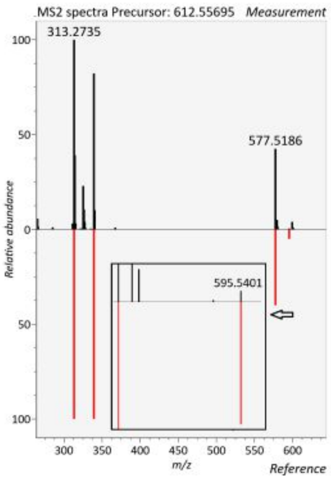

Digalactosyldiacylglycerol (DGDG)
DGDG 14:0-16:1 $[M+NH_4]^+$

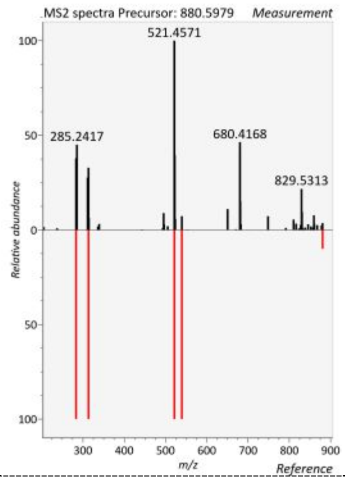

Free fatty acid (FA)
FA 20:0 $[M-H]^-$

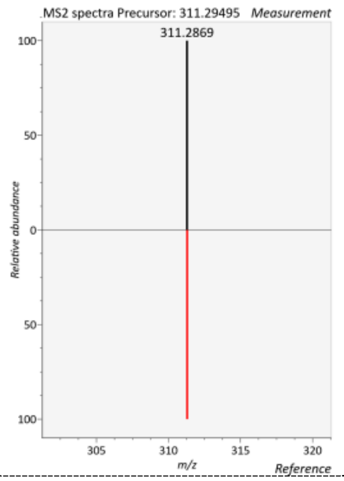

Hemibismonoacylglycerophosphate (HBMP)
HMBP 18:0/18:0-18:1 $[M-H]^-$

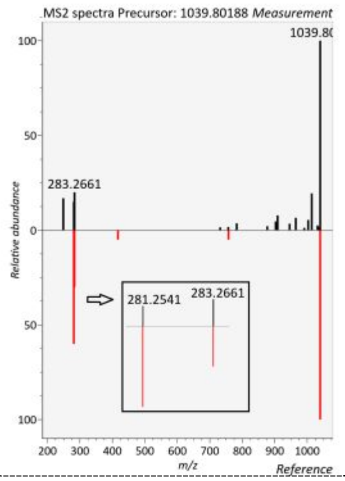

Hexosylceramide non-hydroxy fatty acid-dihydrosphingosine (HexCer-NDS)
HexCer-NDS d18:1/24:0 $[M+H]^+$

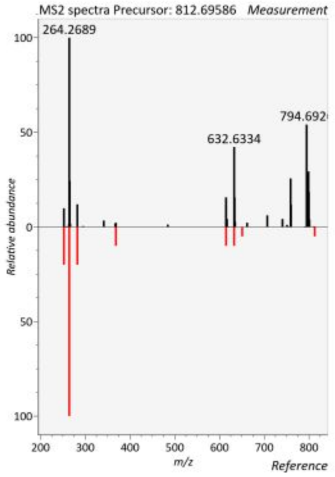

Dihexosylceramide; (HexHexCer)
HexHexCer d18:1/24:1 $[M+H]^+$

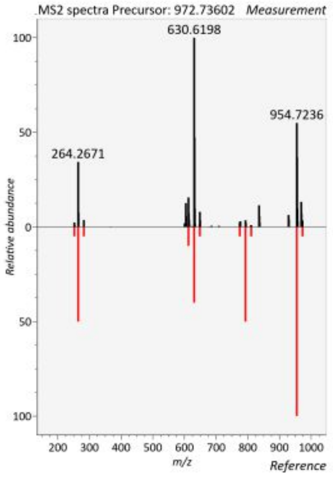

Lysophosphatidylcholine; (LPC)
LPC 18:2 $[M+H]^+$

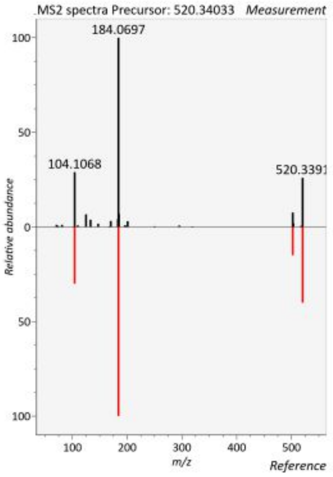

Lysophosphatidylethanolamine; (LPE)
LPE 18:1 $[M-H]^-$

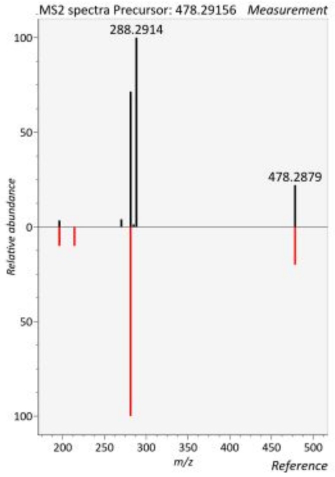

Monogalactosyldiacylglycerol; (MGDG)
MGDG 18:1-20:1 $[M+NH_4]^+$

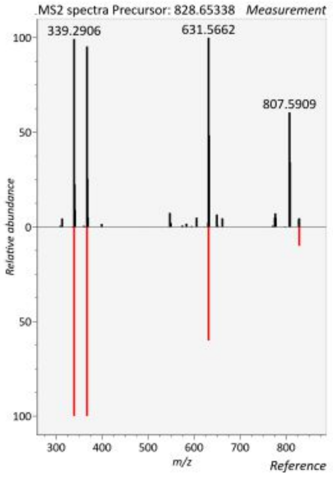

Phosphatidylcholine; (PC)
PC 16:0-18:1 $[M+H]^+$

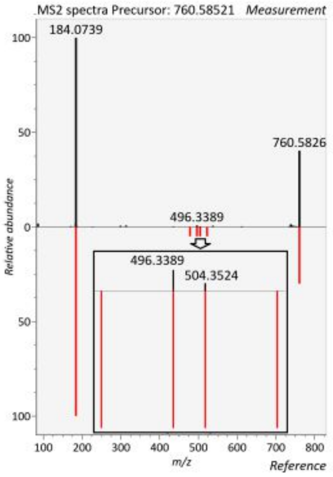

Phosphatidylethanolamine; (PE)
PE 14:0-16:0 $[M-H]^-$

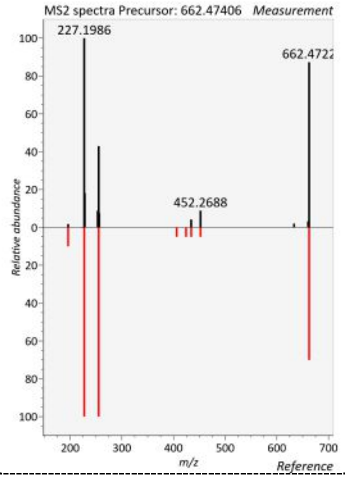

Ceramide phosphoethanolamine (PE-Cer)
PE-Cer t20:1/16:0 $[M-H]^-$

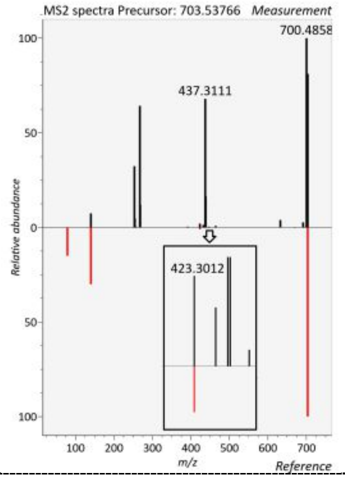

Phosphatidylinositol (PI)
PI 34:2 $[M+NH_4]^+$

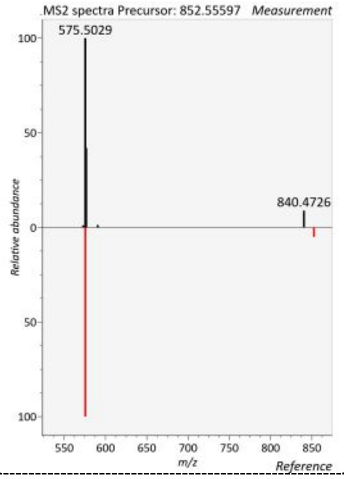

Phosphatidylglycerol (PG)
PG 16:0-16:1 $[M+NH_4]^+$

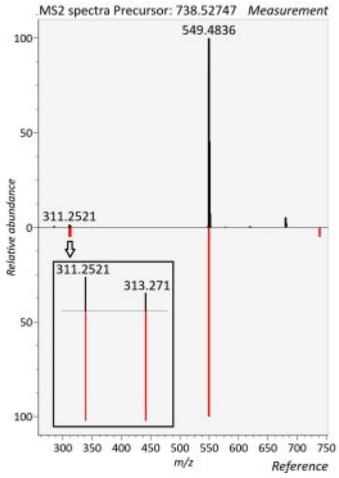

Sulfatide(SHexCer)
SHexCer d38:3 $[M-H]^-$

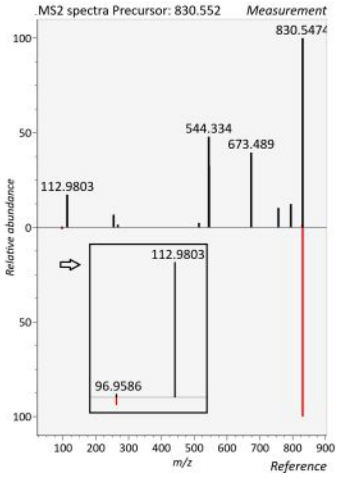

Sphingomyelin (SM)
SM d36:1 $[M+H]^{+*}$

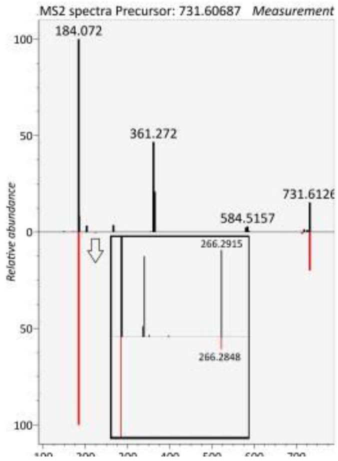

Triacylglycerol (TAG)
TAG 16:0-18:0-18:0 $[M+NH_4]^+$

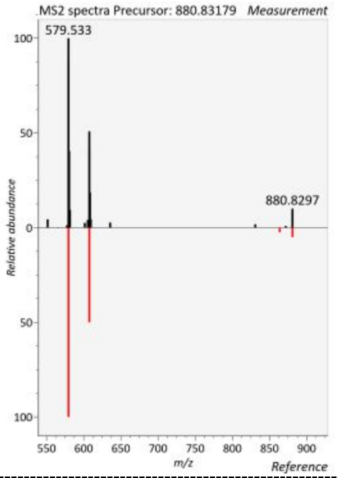

Cer-BDS d34:0+O (Cer-BDS d17:0/17:0+O) $[M+HCOO]^-$

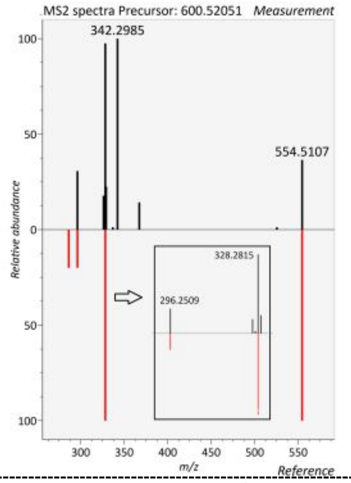

Cer-BS d36:1+O (Cer-BS d18:1/18:0+O) $[M-H]^-$

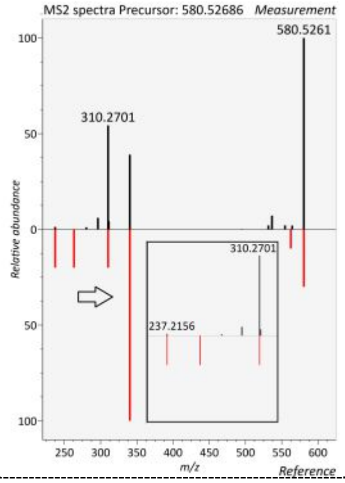

PE 29:2e (PE 16:2e/13:0) $[M+H]^+$

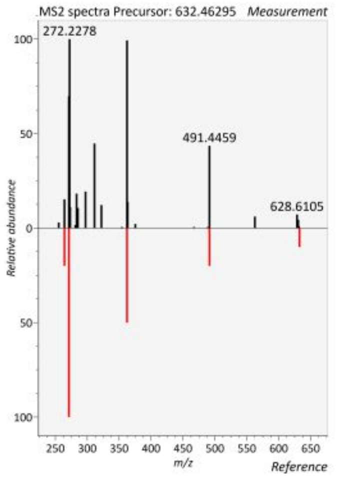

HBMP 48:1 (HBMP 16:0/14:0-18:1) $[M+NH_4]^+$

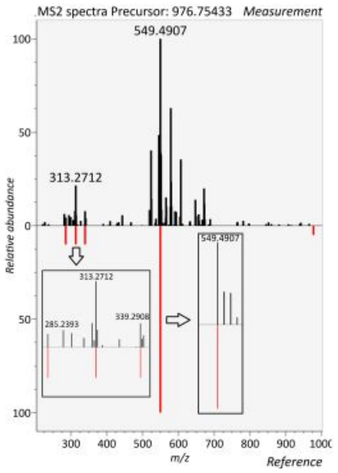

The shown spectra are deconvoluted, measured spectra from the real samples (respective upper spectra in black centroid lines). The respective lower spectra (red centroid lines) represent the reference spectra from MS-DIAL libraries. For the shown SM species also an MLF was detected in MS-DIAL (266.2915), which indicates SM d18:0/18:1. Due to low intensity of the MLF fragments for SMs, species of this class are reported without further information about side chains

Table S15 Exemplary intra-class elution patterns of lipid species. The elution pattern can be used to verify/enhance the confidence of identification. Slight shifts in the regular patterns can derive from chromatographic shifts in the used reference sample

BF samples – positive mode

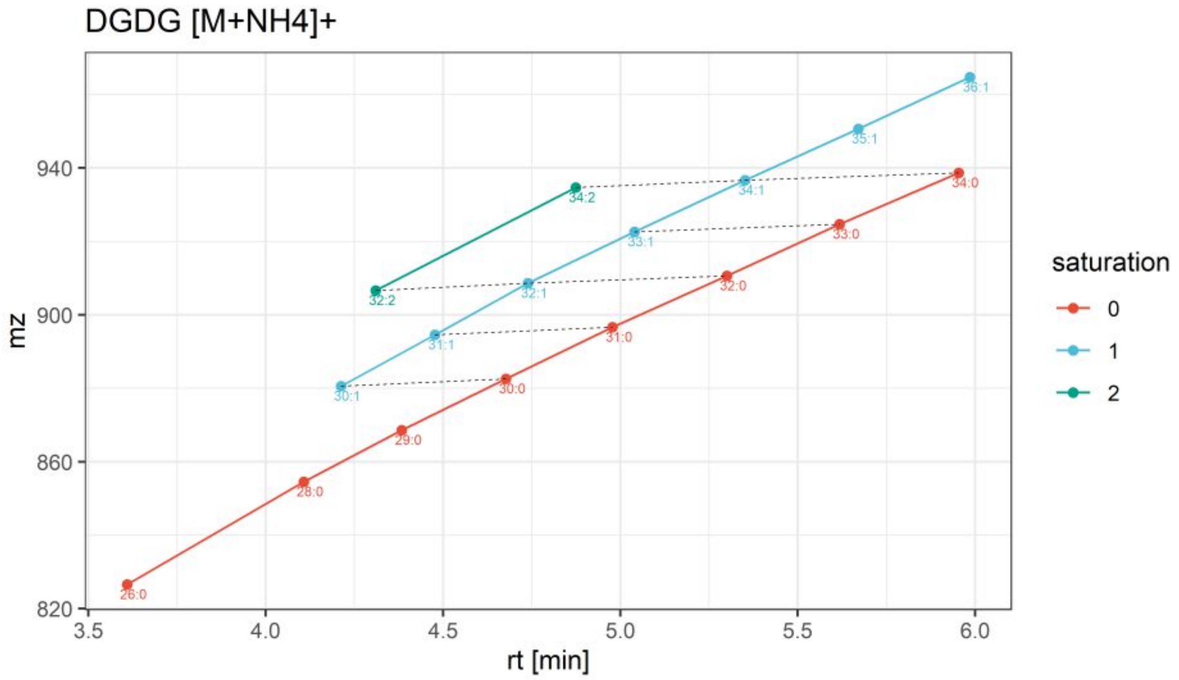

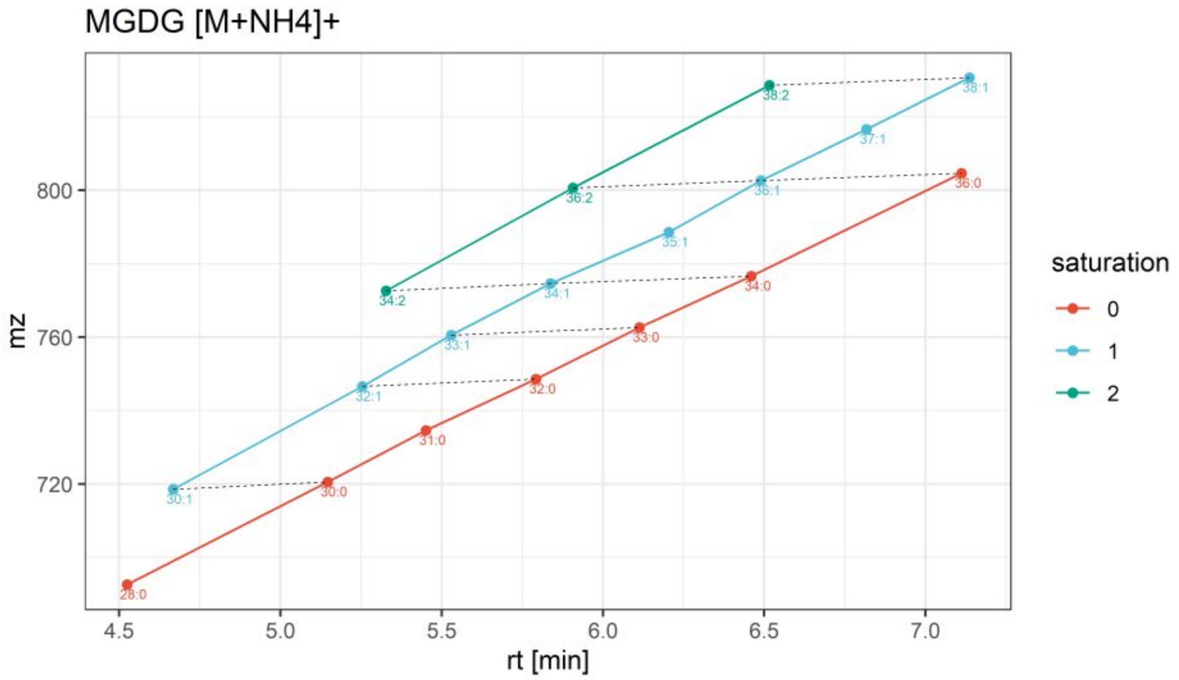

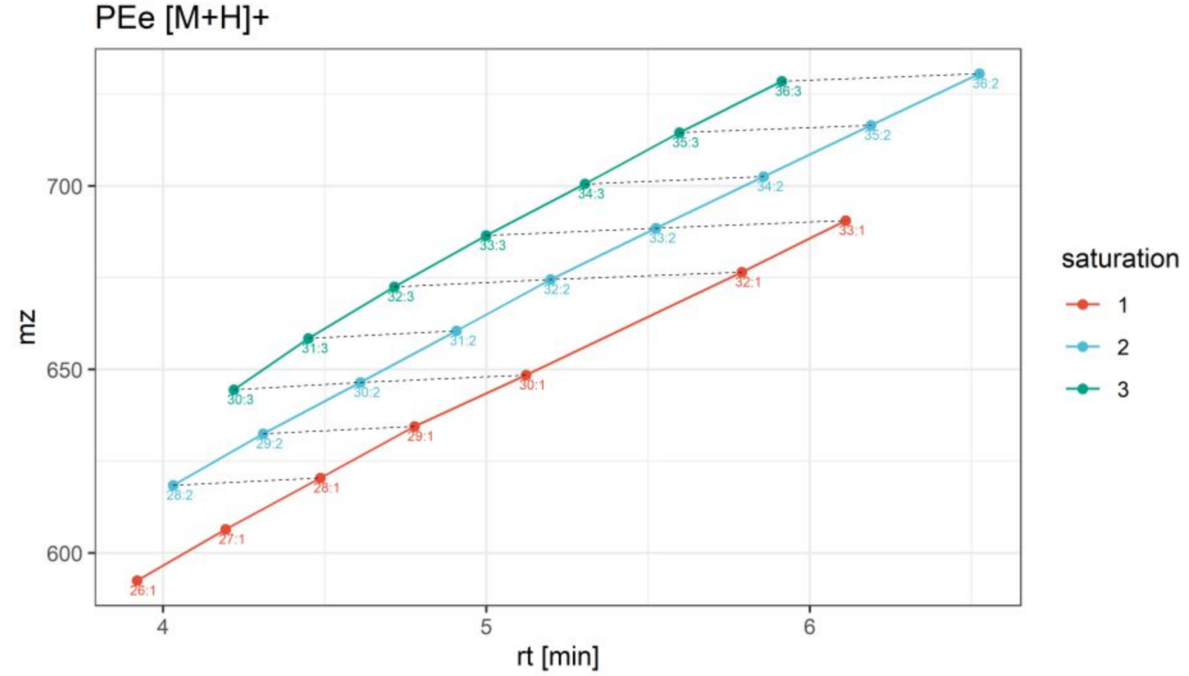

BF samples – negative mode

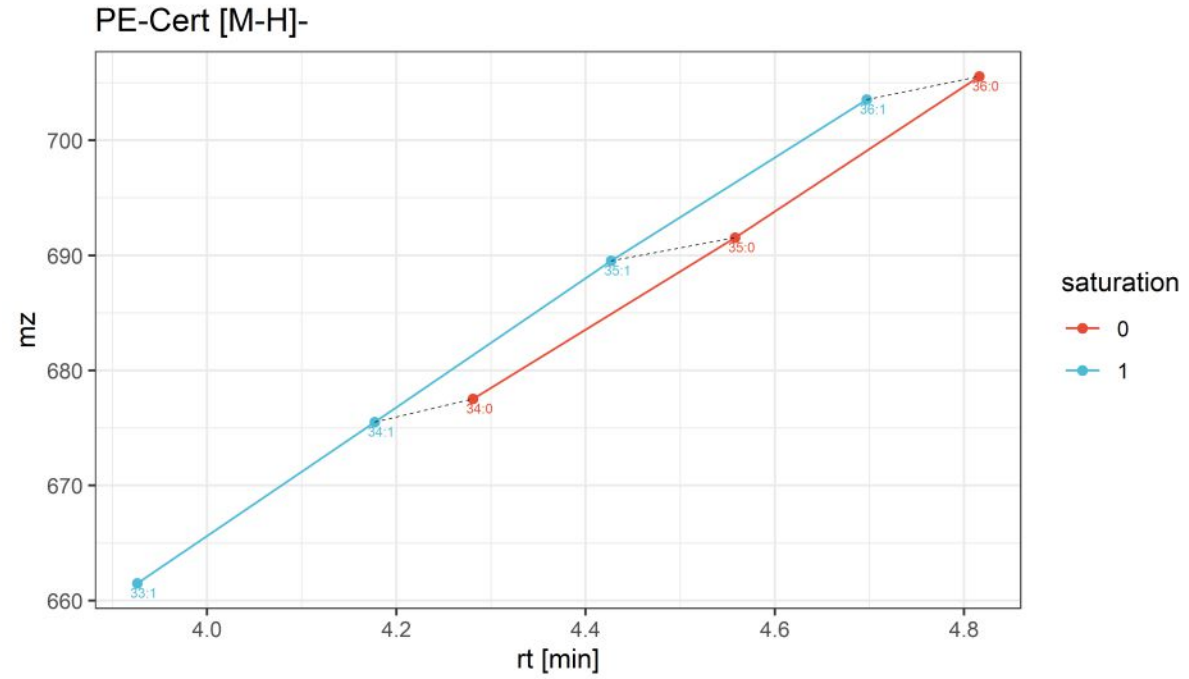

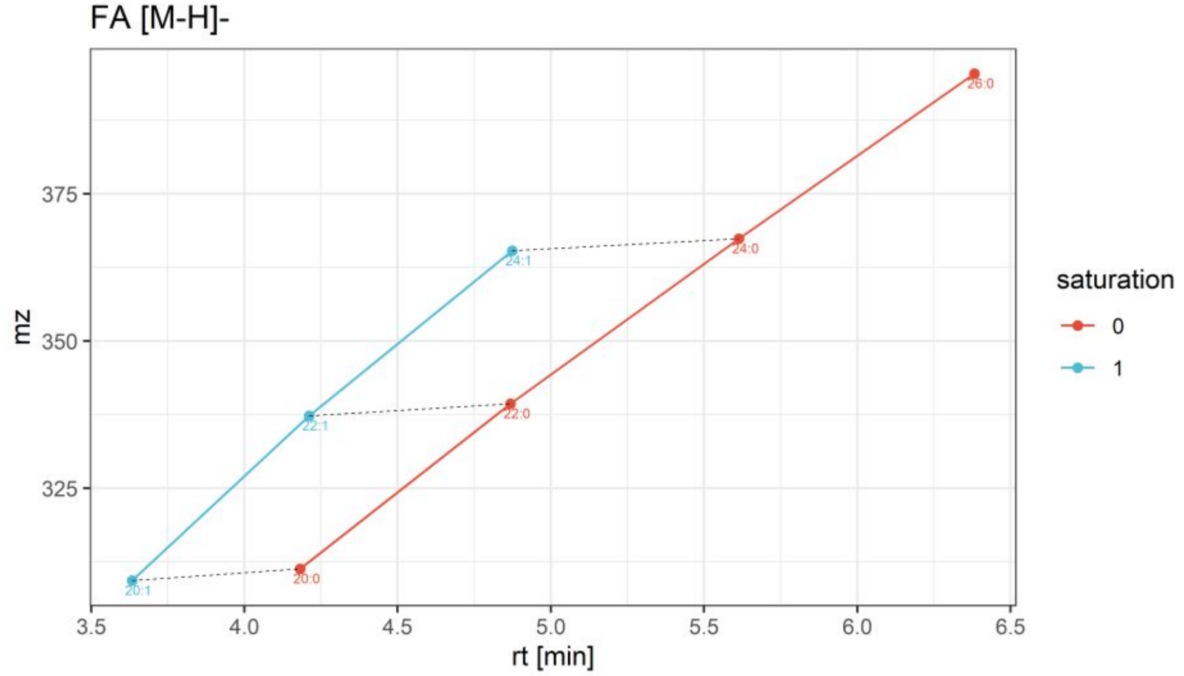

PL samples – positive mode

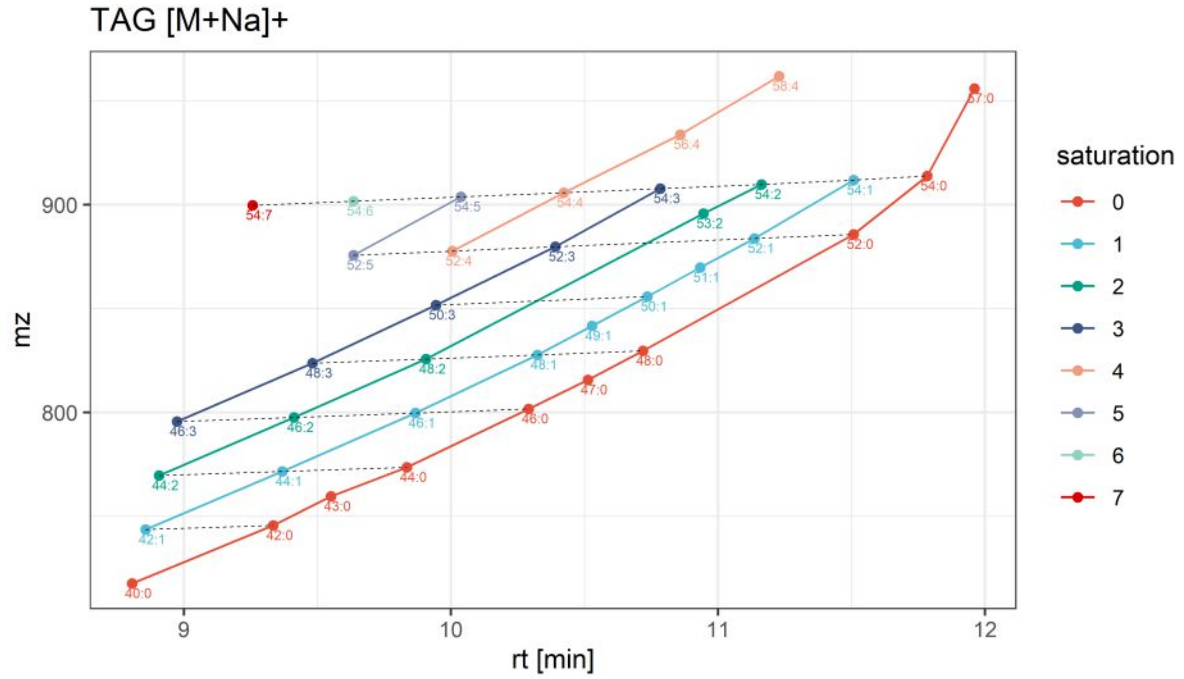

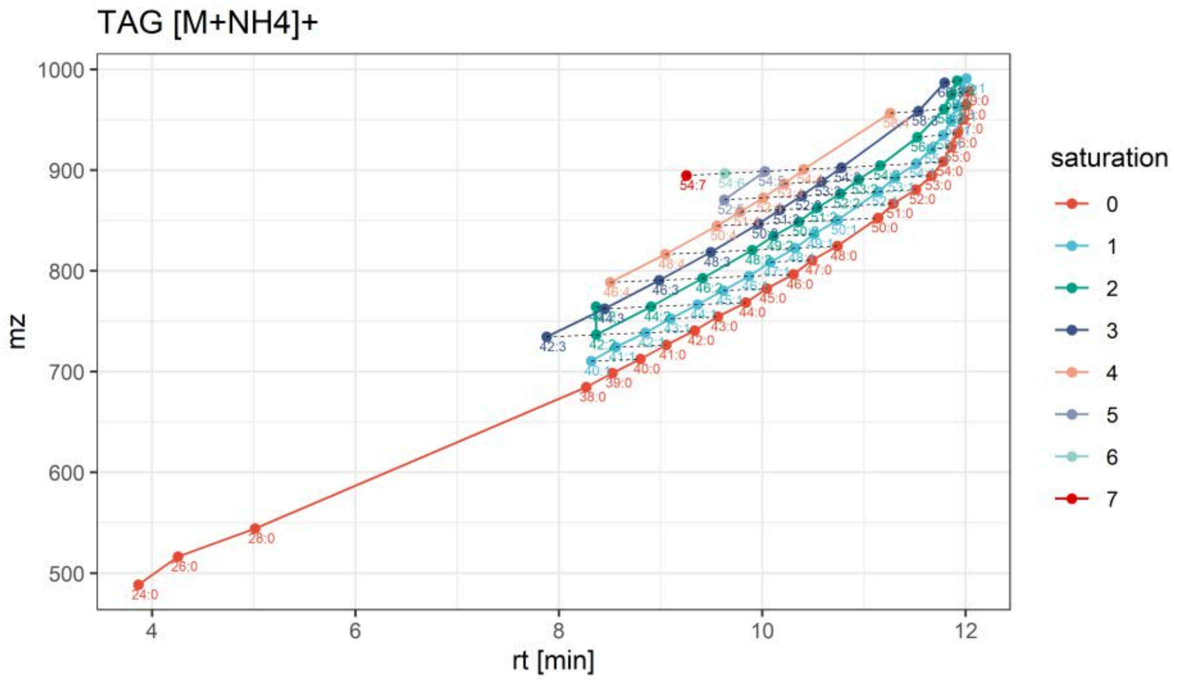

PL samples – negative mode

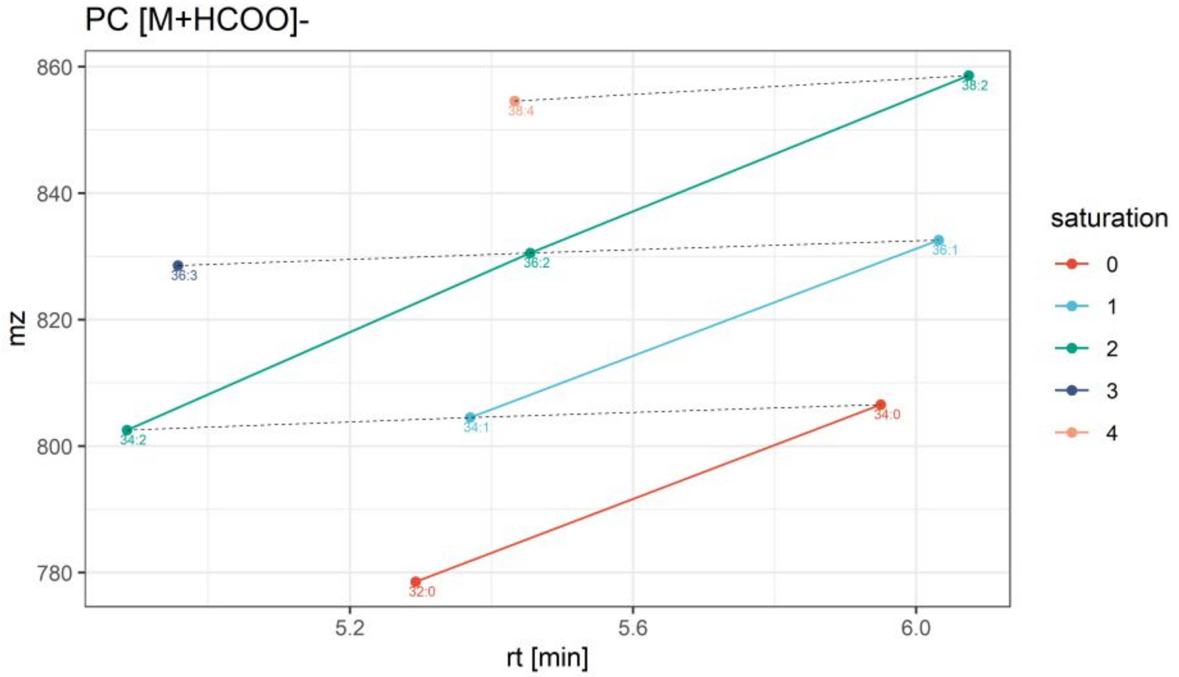

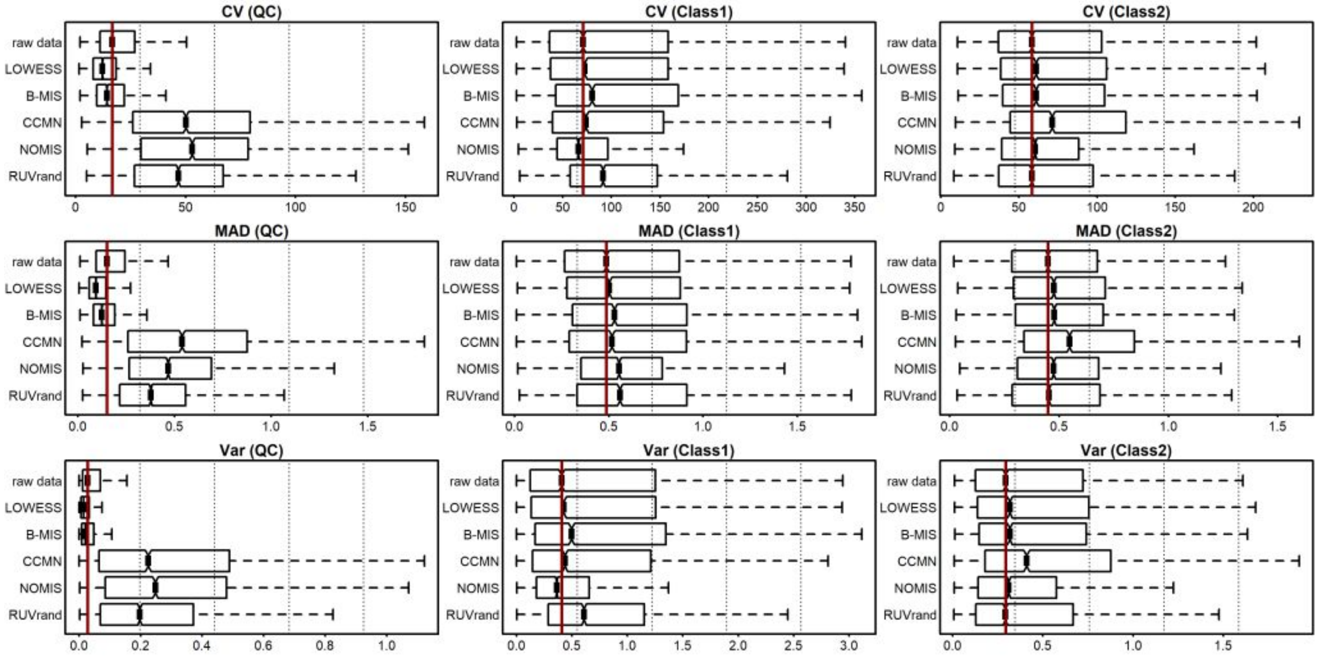

Fig. S1 Comparison of normalization strategies for the comparison of *in vitro* biofilm and *in vivo* plaque samples (BF vs. PL, positive mode) via Box-whisker plots of intragroup metrics of variation. RUVrand was set to $k = 3$. The red line represents the median value in raw height data for a simplified comparison. Raw height data was chosen as the best-performing dataset, since the other normalization methods showed increased metrics of variation in QCs and experimental groups

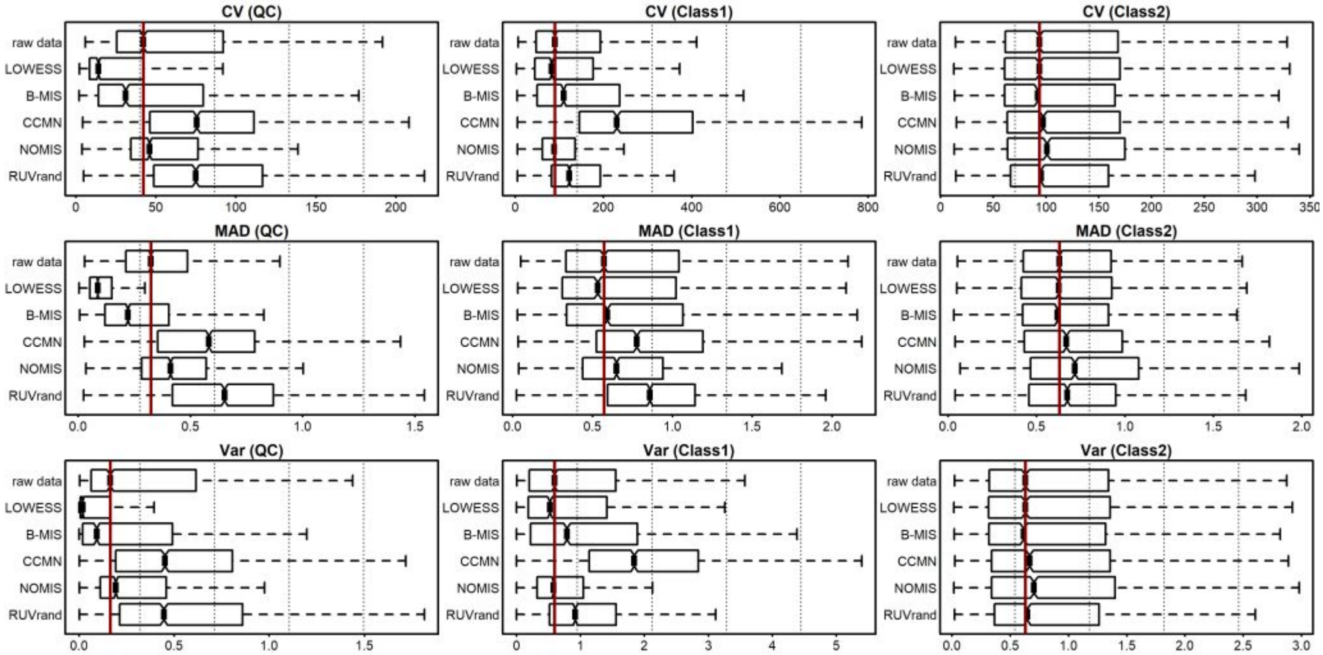

Fig. S2 Comparison of normalization strategies for the comparison of *in vitro* biofilm and *in vivo* plaque samples (BF vs. PL, negative mode) via Box-whisker plots of intragroup metrics of variation. RUVrand was set to $k = 3$. The red line represents the median value in raw height data for a simplified comparison. LOWESS normalized data was chosen as the best-performing dataset, since it showed improved reduction of the metrics of variation in QCs and experimental groups compared to raw data or other normalization methods

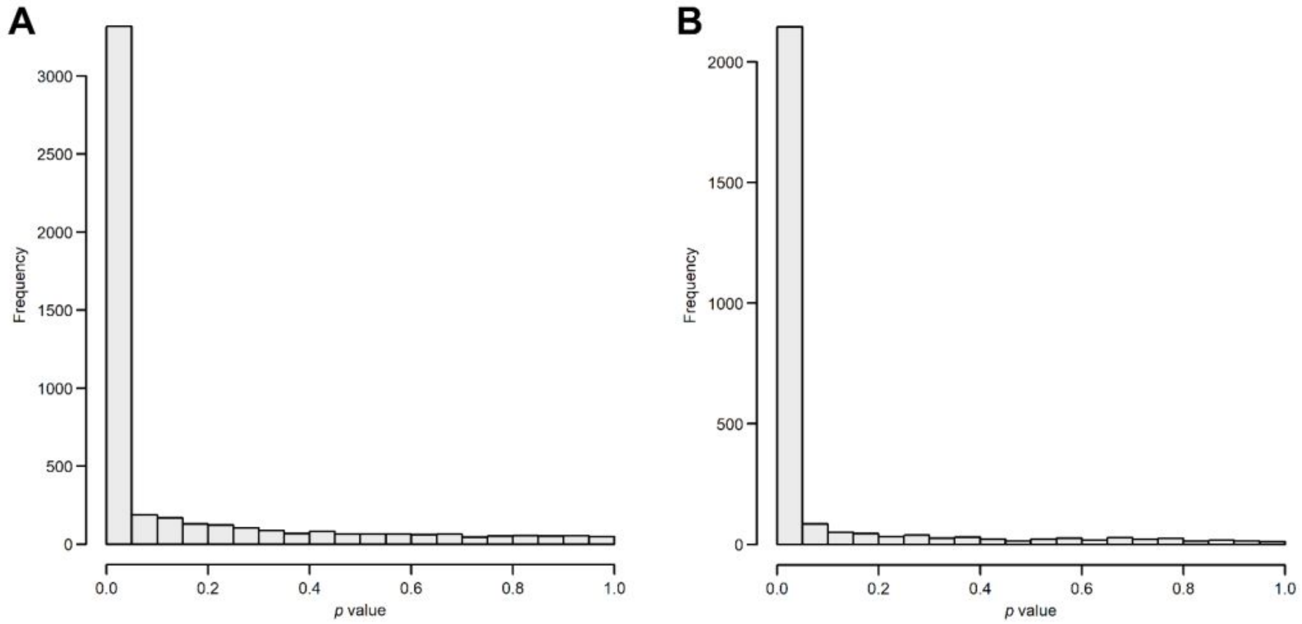

Fig. S3 *p* value distribution (Mann-Whitney *U* test) for the comparison of *in vitro* biofilm and *in vivo* plaque samples (BF vs. PL). A: positive mode (raw height data); B: negative mode (LOWESS normalized data)

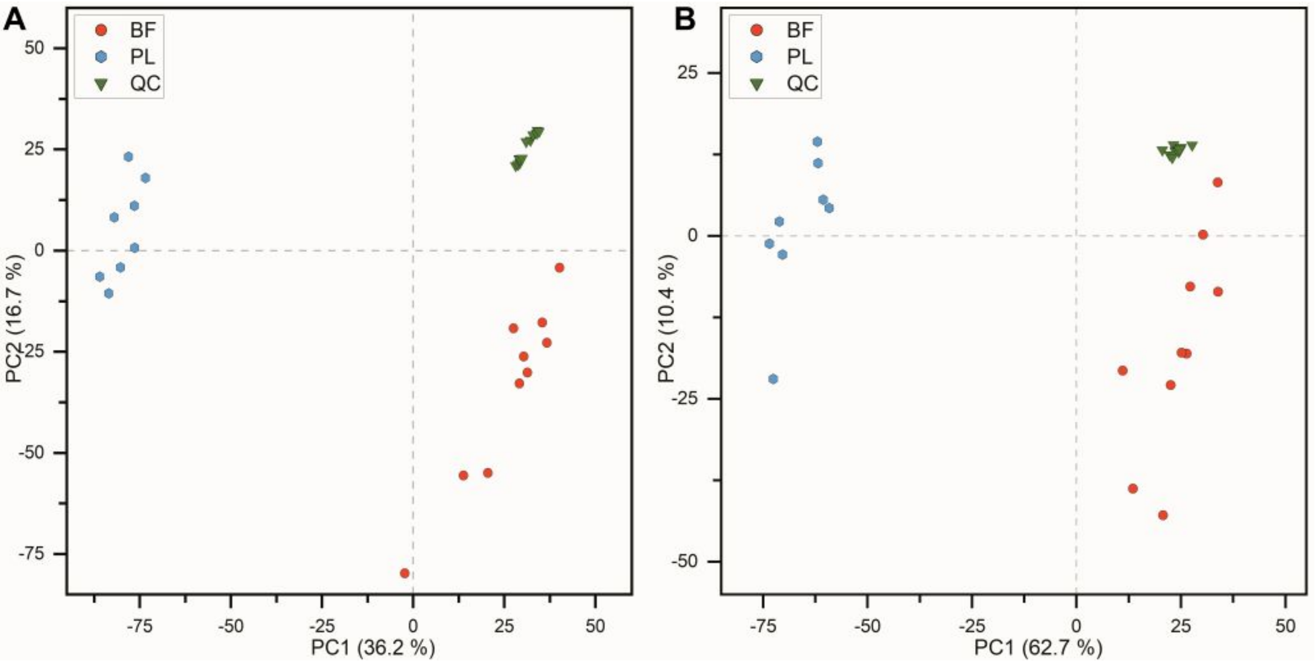

Fig. S4 PCA (scaling: autoscale, weighting: logarithmic) for the comparison of *in vitro* biofilm and *in vivo* plaque samples (BF vs. PL). A: positive mode (raw height data); B: negative mode (LOWESS normalized data)

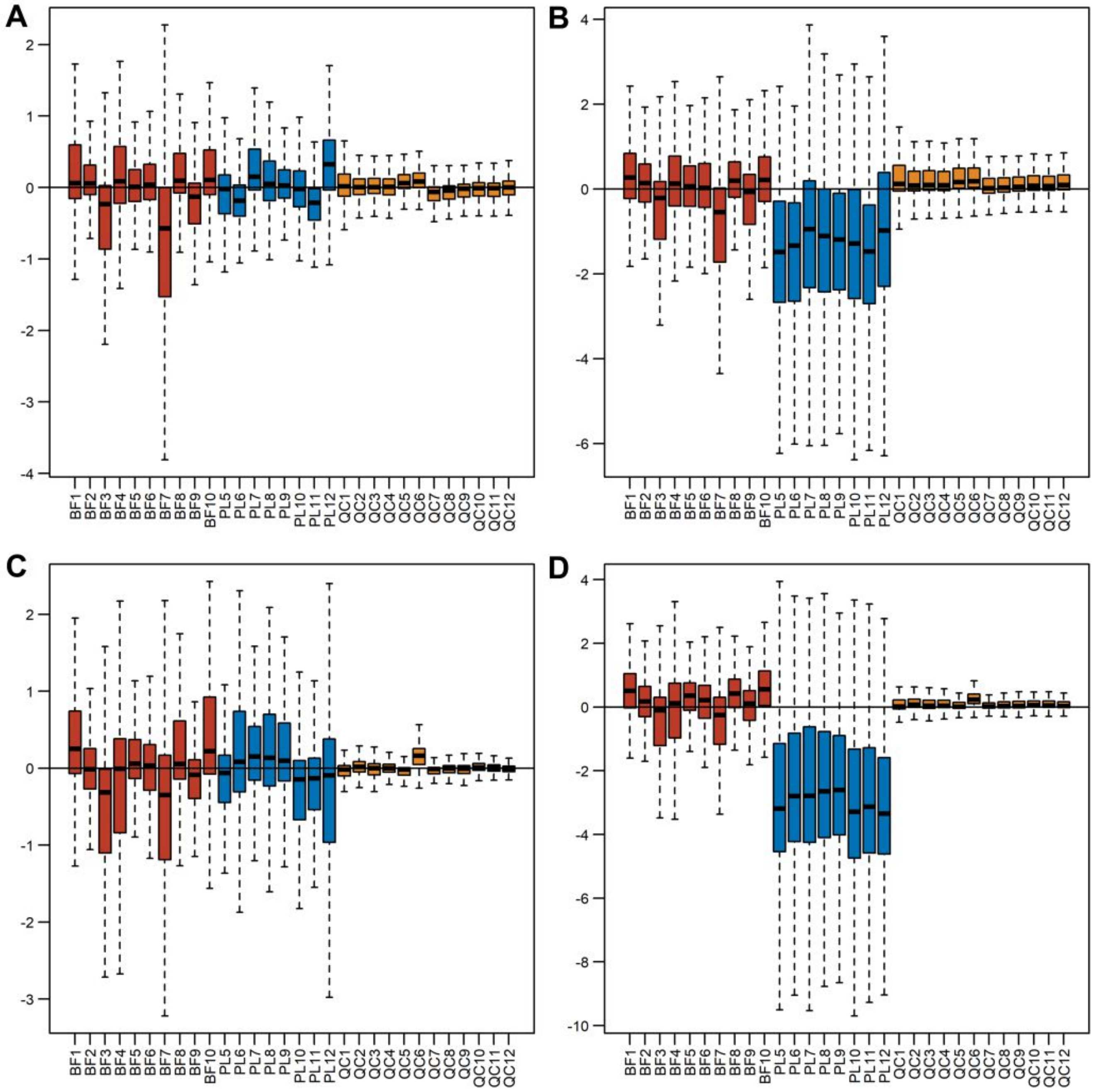

Fig. S5 RLA plots for the comparison of *in vitro* biofilm and *in vivo* plaque samples (BF vs. PL). A: Within-group RLA plot for positive mode (raw height data); B: Within-group negative mode (LOWESS normalized data); C: Across-group RLA plot for positive mode (raw height data); D: Across-group negative mode (LOWESS normalized data)

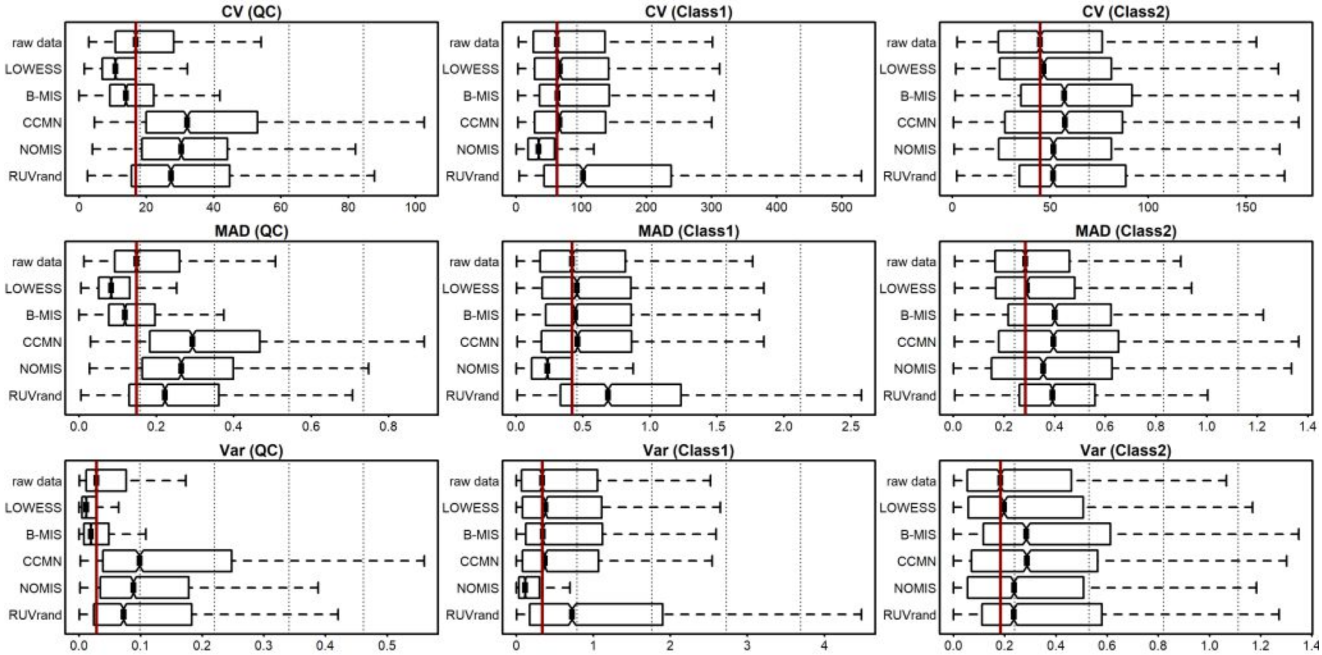

Fig. S6 Comparison of normalization strategies for in vivo plaque samples PL 24h versus PL 72h (positive mode) via Box-whisker plots of intragroup metrics of variation. RUVrand was set to k = 3. The red line represents the median value in raw height data for a simplified comparison. Raw height data was chosen as the best-performing dataset, since the other normalization methods showed increased metrics of variation in QCs and experimental groups

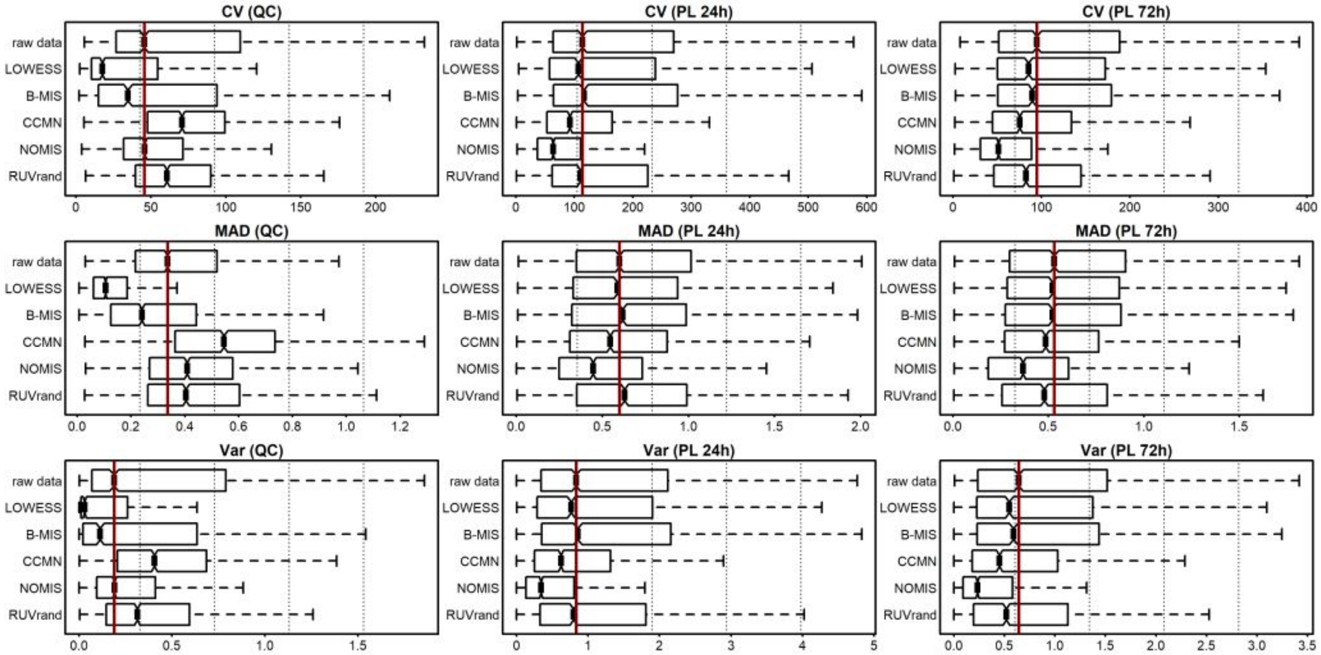

Fig. S7 Comparison of normalization strategies for *in vivo* plaque samples PL 24h versus PL 72h (negative mode) via Box-whisker plots of intragroup metrics of variation. RUVrand was set to $k = 3$. The red line represents the median value in raw height data for a simplified comparison. LOWESS normalized data was chosen as the best-performing dataset, since it showed improved reduction of the metrics of variation in QCs and experimental groups compared to raw data or other normalization methods

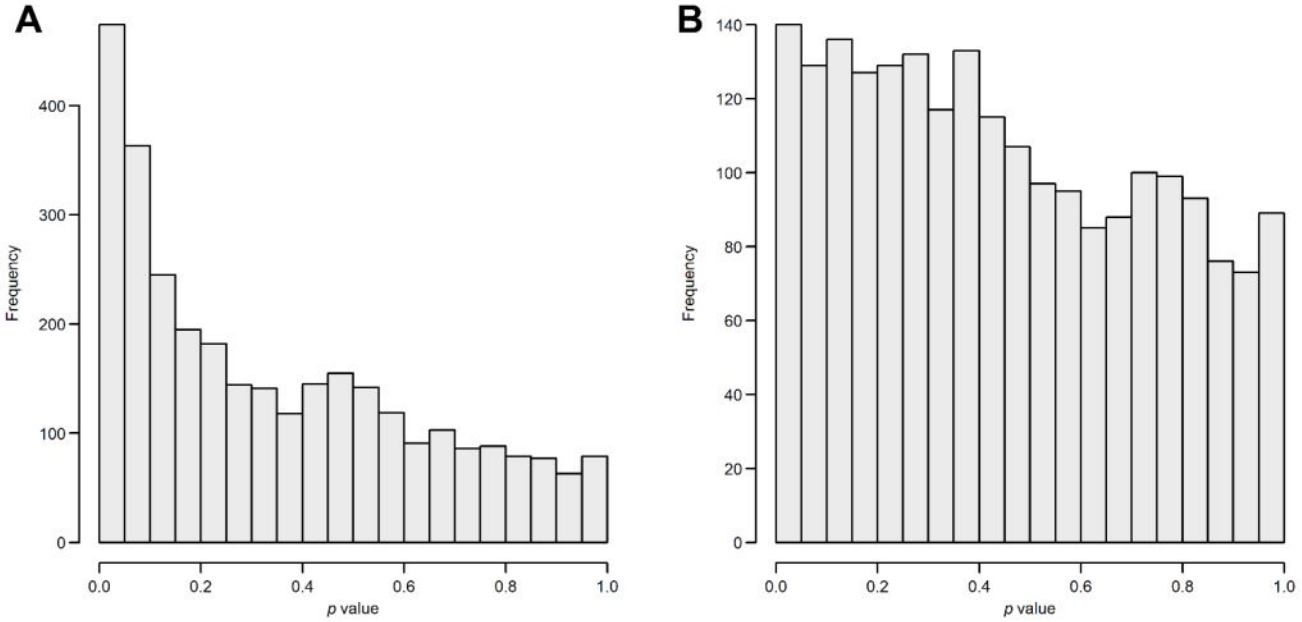

Fig. S8 *p* value distribution (Wilcoxon signed-rank test) for the comparison of different formation times of *in vivo* plaque (PL 24h vs. PL 72h). A: positive mode (raw height data); B: negative mode (LOWESS normalized data)

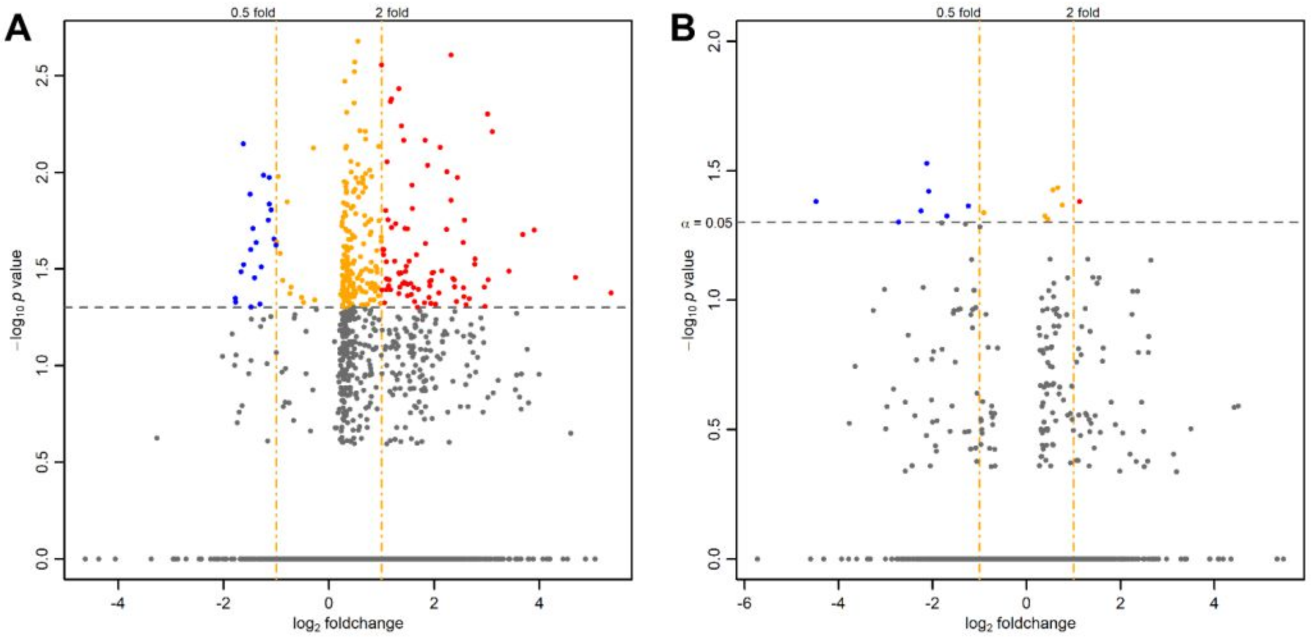

Fig. S9 Volcano plots with SGoF adjusted *p* values (Wilcoxon signed-rank test) and median foldchanges for the comparison of different formation times of *in vivo* plaque (PL 24h vs. PL 72h). In this case SGoF was chosen for both datasets as it was less strict than FDR correction. A: positive mode (raw height data); B: negative mode (LOWESS normalized data)

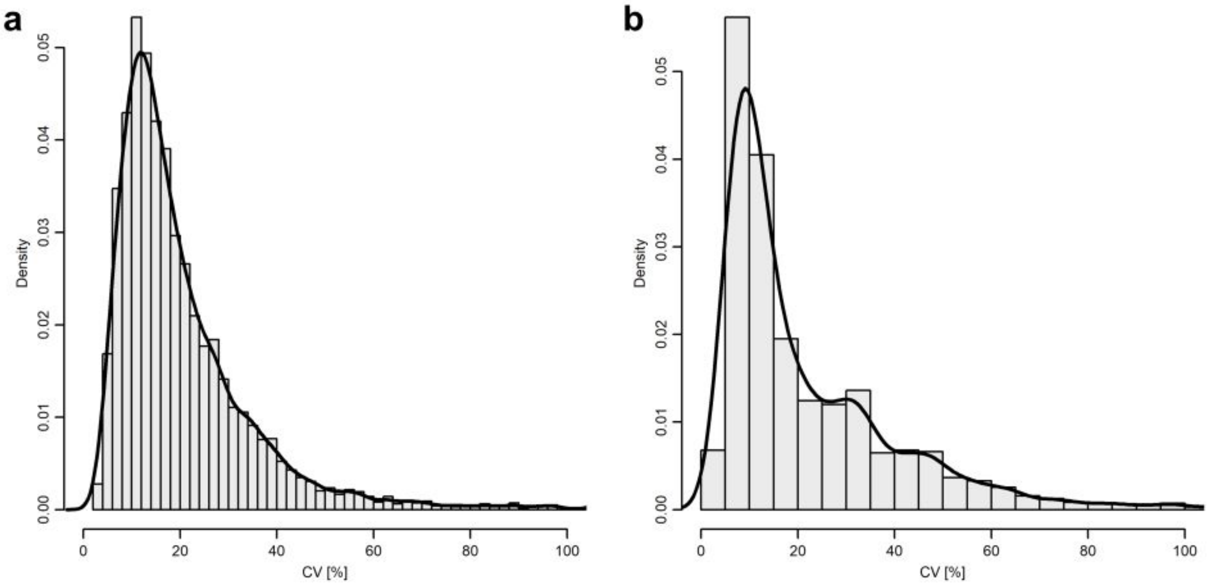

Fig. S10 Distribution of the coefficient of variation (CV) in QC samples. A: positive mode (raw height data), 81.7% of all features showed a CV <30%; B: negative mode (LOWESS-normalized data), 73.7% of all features showed a CV <30%
